# Tolerability and immunogenicity of an intranasally-administered adenovirus-vectored COVID-19 vaccine: An open-label partially-randomised ascending dose phase I trial

**DOI:** 10.1016/j.ebiom.2022.104298

**Published:** 2022-10-10

**Authors:** Meera Madhavan, Adam J. Ritchie, Jeremy Aboagye, Daniel Jenkin, Samuel Provstgaad-Morys, Iona Tarbet, Danielle Woods, Sophie Davies, Megan Baker, Abigail Platt, Amy Flaxman, Holly Smith, Sandra Belij-Rammerstorfer, Deidre Wilkins, Elizabeth J. Kelly, Tonya Villafana, Justin A. Green, Ian Poulton, Teresa Lambe, Adrian V.S. Hill, Katie J. Ewer, Alexander D. Douglas

**Affiliations:** a Jenner Institute, University of Oxford, Old Road Campus Research Building, Oxford OX3 7BN, UK; b Centre for Clinical Vaccinology and Tropical Medicine, University of Oxford, Churchill Hospital, Oxford OX3 7LE, UK; c Translational Medicine, Vaccines & Immune Therapies, BioPharmaceuticals R&D, AstraZeneca, 1 Medimmune Way, Gaithersburg, MD 20878, USA; d Clinical Development, Vaccines & Immune Therapies, BioPharmaceuticals R&D, AstraZeneca, Gaithersburg, MD, USA; e Clinical Development, Vaccines & Immune Therapies, BioPharmaceuticals R&D, AstraZeneca, Cambridge, UK; f Oxford Vaccine Group, Centre for Clinical Vaccinology and Tropical Medicine, University of Oxford, Churchill Hospital, Oxford OX3 7LE, UK; g China Academy of Medical Sciences Oxford Institute, University of Oxford, NDM Research Building, Old Road Campus, Headington, Oxford OX3 7FZ, UK

**Keywords:** Adenovirus vector, Intranasal vaccination, SARS-CoV-2, Mucosal antibody

## Abstract

**Background:**

Intranasal vaccination may induce protective local and systemic immune responses against respiratory pathogens. A number of intranasal SARS-CoV-2 vaccine candidates have achieved protection in pre-clinical challenge models, including ChAdOx1 nCoV-19 (AZD1222, University of Oxford / AstraZeneca).

**Methods:**

We performed a single-centre open-label Phase I clinical trial of intranasal vaccination with ChAdOx1 nCoV-19 in healthy adults, using the existing formulation produced for intramuscular administration.

Thirty SARS-CoV-2 vaccine-naïve participants were allocated to receive 5 × 10^9^ viral particles (VP, *n=*6), 2 × 10^10^ VP (*n=*12), or 5 × 10^10^ VP (*n=*12). Fourteen received second intranasal doses 28 days later. A further 12 received non-study intramuscular mRNA SARS-CoV-2 vaccination between study days 22 and 46.

To investigate intranasal ChAdOx1 nCoV-19 as a booster, six participants who had previously received two intramuscular doses of ChAdOx1 nCoV-19 and six who had received two intramuscular doses of BNT162b2 (Pfizer / BioNTech) were given a single intranasal dose of 5 × 10^10^ VP of ChAdOx1 nCoV-19.

Objectives were to assess safety (primary) and mucosal antibody responses (secondary).

**Findings:**

Reactogenicity was mild or moderate. Antigen-specific mucosal antibody responses to intranasal vaccination were detectable in a minority of participants, rarely exceeding levels seen after SARS-CoV-2 infection. Systemic responses to intranasal vaccination were typically weaker than after intramuscular vaccination with ChAdOx1 nCoV-19. Antigen-specific mucosal antibody was detectable in participants who received an intramuscular mRNA vaccine after intranasal vaccination. Seven participants developed symptomatic SARS-CoV-2 infection.

**Interpretation:**

This formulation of intranasal ChAdOx1 nCoV-19 showed an acceptable tolerability profile but induced neither a consistent mucosal antibody response nor a strong systemic response.

**Funding:**

AstraZeneca.

**Research in context**

**Evidence before this study**

To identify relevant studies a Pubmed search was undertaken on 26 June 2022 using the following search terms: (intranasal OR nasal OR mucosal) AND (coronavirus OR COVID-19 OR SARS-CoV-2) AND (vaccine) AND (clinical trial).

No time or language restrictions were used. The authors’ personal databases were also reviewed for relevant literature.

Only two results reported clinical trials of intranasal COVID-19 vaccines.^1^^,^^2^ One study (NCT04871737) found that two doses of an intranasally-administered live recombinant Newcastle disease virus expressing the SARS-CoV-2 spike protein induced detectable systemic antibody and T-cell responses, but these were weaker than when the same product was administered intramuscularly.^1^ Mucosal responses were not reported. A second report described Phase I and II studies of a live-attenuated influenza virus vector expressing the SARS-CoV-2 spike receptor binding domain (ChiCTR2000037782, ChiCTR2000039715, ChiCTR2100048316): systemic and mucosal immune responses were each detected in a minority of volunteers.^2^

At least ten other intranasal COVID-19 vaccines have been evaluated in as-yet-unpublished clinical trials, including four adenovirus-vectored vaccines other than ChAdOx1 nCoV-19.^3^

Trials of aerosolised administration of a human adenovirus-vectored vaccine (using a nebuliser device, rather than a nasal spray) have reported induction of systemic immune responses, but did not report mucosal immune responses.^4^^,^^5^

**Added value of this study**

We present a first-in-human study of intranasal COVID-19 vaccination with an adenovirus-vectored vaccine. Reactogenicity was acceptable at all doses but immunogenicity was insufficient to warrant further development of the current formulation / device combination.

**Implications of all the available evidence**

There remains a need for clinical development of needle-free vaccines capable of inducing consistent protective mucosal immune responses. Although the vaccine and delivery device combination in this study did not warrant further exploration, optimisation of this vaccine and other candidates for mucosal delivery remains a key opportunity for transmission blocking vaccines.

Alt-text: Unlabelled box

## Introduction

There are unmet needs for COVID-19 vaccines which induce robust and long-lasting protection against mild infection and transmission, especially with antigenically-variant viral strains, and for vaccines which are suitable for needle-free administration.

Upper airway epithelial cells are highly susceptible to SARS-CoV-2 and are believed to be the most likely site of initial infection.^6^^,^^7^ Viral infections of respiratory mucosa can induce, and may be prevented by local mucosal immune responses. Such responses include secretory IgA, mucosal-homing plasmablasts, and resident memory T cells.^8^, ^9^, ^10^ As compared to IgG, the polymeric structure of secreted IgA molecules may contribute to superior virus neutralization potency, and possibly greater breadth of neutralization of antigenically-diverse viruses.^11^, ^12^, ^13^ In mouse models of influenza, passive administration of purified IgA to the respiratory tract can protect against infection, and (unlike serum IgG) appears capable of abrogating nasal virus shedding at levels matching those seen in previously-infected convalescent animals.^12^^,^^14^ After intranasal exposure to influenza haemagglutinin, transgenic mice deficient in polymeric secreted IgA have substantially reduced protection against subsequent infection, as compared to wild-type mice.^15^ Mucosal antibody, including IgA, also appears to contribute to protection against respiratory syncytial virus.^16^, ^17^, ^18^, ^19^

Intranasal live-attenuated influenza vaccines are efficacious and used extensively in some countries – in particular for school-age children, highlighting the practical advantages of this delivery route.^20^ As compared to IM flu vaccination, live-attenuated IN vaccination induces stronger mucosal IgA responses and weaker systemic antibody responses.^21^

In individuals without prior SARS-CoV-2 infection, mucosal IgA responses after intramuscular (IM) SARS-CoV-2 vaccination appear relatively weak and short-lived.^22^, ^23^, ^24^ Intranasal (IN) SARS-CoV-2 vaccination is thus immunologically attractive, and potentially complementary to the effectiveness of intramuscular SARS-CoV-2 vaccination against severe systemic consequences of infection.^25^

Several intranasal SARS-CoV-2 vaccines are in development, and have recently been reviewed.^3^ Many of these are based upon live-attenuated respiratory viruses or replication-incompetent viral vectors with mucosal tropism, including influenza, para-influenza viruses, Newcastle Disease virus, SARS-CoV-2 itself, and adenoviruses. There is increasing evidence that such approaches can achieve robust protection against SARS-CoV-2 infection in animal models, including reducing nasal shedding of the virus,^26^^,^^27^ and also in the context of intranasal boosting following intramuscular priming vaccination.^28^ Although at least 12 of these candidates have entered clinical trials, there is as yet little published data from these clinical studies.^3^ It has been reported that two doses of an intranasally-administered live recombinant Newcastle disease virus expressing the SARS-CoV-2 spike protein induced detectable systemic antibody and T-cell responses in some volunteers.^1^ These were however substantially weaker than when the same product was administered intramuscularly, mucosal immune responses were not reported, and several symptomatic SARS-CoV-2 infections were observed after vaccination. In trials of a live-attenuated influenza virus vector expressing the SARS-CoV-2 spike receptor binding domain, two intranasal doses induced systemic and mucosal immune responses in a minority of volunteers.^2^

At least five adenovirus-vectored candidates are among the mucosally-delivered SARS-CoV-2 vaccines which have entered clinical trials.^3^ In previous clinical trials of intranasal adenovirus-vectored vaccines targeting other pathogens, systemic immune responses have been detectable, although there is little published data on the mucosal responses induced^29^^,^^30^ (also NCT03232567 and NCT00755703).

ChAdOx1 nCoV-19 / AZD1222, the replication-incompetent adenovirus-vectored COVID-19 vaccine developed by the University of Oxford and AstraZeneca, is efficacious after intramuscular use.^31^, ^32^, ^33^, ^34^ More than two billion doses of the product have been distributed.^35^ Intranasal administration of ChAdOx1 nCoV-19 protected against SARS-CoV-2 challenge in hamsters and non-human primates (NHPs).^26^ Rather than formulation/device combinations specifically optimised for IN vaccination, both our NHP study and, to our knowledge, previous clinical trials of other IN adenovirus-vectored vaccines have employed off-the-shelf spray devices produced for other IN drugs, with formulations designed primarily to achieve viral stability in storage, as developed for IM use.^36^

Here, we report a Phase I clinical trial evaluating the safety and immunogenicity of intranasally-administered ChAdOx1 nCoV-19, both in vaccine-naïve participants and in participants who had previously received intramuscularly-administered SARS-CoV-2 vaccines.

## Methods

### Ethics and regulation

The study was approved by the Medicines and Healthcare Products Regulatory Agency (MHRA; reference CTA 21584/0443/001), and the NHS London – Surrey Borders Research Ethics Committee (reference 21/HRA/0699). The trial was registered at clinicaltrials.gov (identifier NCT04816019). Written informed consent was obtained from all participants, and the trial was conducted in accordance with the principles of the Declaration of Helsinki and Good Clinical Practice (GCP).

An independent data safety and monitoring board (DSMB) provided safety oversight of the trial. Enrolment was staggered to allow for interim safety reviews to be performed by the chief investigator 72 h after first vaccinations at the low and high dose levels, and additionally by the by the DSMB 7 days after vaccination of the sixth (final) volunteer in group 1 (low dose) and before the first administration of a second intranasal dose. Comprehensive details of safety reviews and holding rules are provided in the study protocol (see Supplementary Material).

### Study design

COV008 was an open-label phase I clinical trial conducted at a single centre (the Centre for Clinical Vaccinology and Tropical Medicine, University of Oxford), with non-randomised group allocation.

The primary objective was to evaluate the safety and tolerability of IN ChAdOx1 nCoV-19. The secondary objective was to assess the mucosal immune response to IN ChAdOx1 nCoV-19, and specifically the induction of anti-spike (anti-S) antibody in nasal mucosal lining fluid (NMLF).

The study recruited healthy adults under the age of 55. Full details on inclusion and exclusion criteria can be found in the study protocol (see Supplementary Materials), and details on eligible age ranges at different stages of the study are provided below. History of previous COVID-19 infection was not an exclusion criterion. Approved advertising targeted the Thames Valley region, UK. Prospective participants were required to complete an online questionnaire covering key exclusion criteria and were then invited for a screening visit if potentially eligible. Following informed consent, they were assessed for full eligibility at this visit where a medical history, physical examination, urinalysis, and clinical blood tests were performed. A summary of medical history was obtained from each volunteer's general practitioner prior to vaccination.

The study used the same formulation of ChAdOx1 nCoV-19 as is licensed for intramuscular use in the UK.^37^ The product had been manufactured in accordance with Good Manufacturing Practice. Vaccine was administered in a semi-recumbent position using a MAD300 intranasal mucosal atomization device (Teleflex Medical, Penn, US) and was equally divided between the two nostrils.

The first phase of the study enrolled COVID-19 vaccine-naïve participants. The first group to be vaccinated received 5 × 10^9^ virus particles (VP, group 1, henceforth ‘low dose’), followed by subsequent groups receiving 5 × 10^10^ VP (group 2, henceforth ‘high dose’) and 2 × 10^10^ VP (group 3, henceforth ‘mid dose’). Planned sample sizes for groups 1–3 were 6, 24 and 24 respectively. Given the descriptive nature of the objectives, the sample size was based upon the investigators’ judgment of the volume of safety and immunogenicity data required to permit an informed decision about expansion to a Phase II study, rather than upon calculation of a sample size to provide power for statistical inference. Allocation to dose levels was non-randomised, with volunteers allocated to the next convenient vaccination appointment after completion of screening.

At enrolment, volunteers in groups 1–3 were randomised 1:1 without blocking to receive only a single IN vaccination, or to receive a second IN vaccination 28 days later (at the same dose level as the first). The randomisation list was generated by the data manager using Sealed Envelope's simple randomiser (www.sealedenvelope.com). It was accessed by a member of the clinical study team using the randomisation module in the REDCap trial database (REDCap software, version 12.0 (Vanderbilt University)).

A second phase of the study enrolled individuals who had previously received two IM doses of ChAdOx1 nCoV-19 (group 4, planned *n=*6) or two IM doses of BNT162b2 (group 5, planned *n=*6), with the second dose of either IM vaccine having been administered at least 12 weeks before enrolment. These individuals received a single IN dose of 5 × 10^10^ VP ChAdOx1 nCoV-19 at enrolment.

The study took place in the context of the UK's national rollout of intramuscular COVID-19 vaccines and widespread community transmission of SARS-CoV-2 in the local area. In accordance with the study's ethical approval, participants were requested to refrain from receiving non-study intramuscular COVID-19 vaccines for 28 days after IN vaccination (including the second IN vaccination, for those who received it).

After the enrolment of the first participant in the study, recruitment was paused due to emerging information about extremely rare incidents of thrombosis with thrombocytopenia syndrome (TTS) after IM vaccination with adenovirus-vectored COVID-19 vaccines.^38^ Recruitment was re-started after discussion with the MHRA, ethics committee, and the DSMB. An amendment with a range of measures designed to minimise risk of TTS was implemented (see study protocol). This included narrowing the eligible age range from 18–40 years to 30–40 years. Following further discussion with regulators regarding the risk – benefit balance of study participation, the protocol was further amended to broaden the eligible age range to 18–55 for vaccine-naïve individuals (groups 1–3), and 30–55 for individuals who had previously received intramuscular COVID-19 vaccination (groups 4–5).

### Follow-up and clinical data collection

Following vaccination, all participants attended follow up at these nominal timepoints: day 7, 14, 28, 56 and 112. Volunteers who received a second intranasal vaccine dose on day 28 attended additional visits on days 35 and 42, and selected volunteers attended an additional visit on day 1 or day 3. Participants were questioned regarding the occurrence of SAEs at all timepoints. Participants were also required to complete an online daily symptom diary for 28 days following each vaccination, including an initial 7 day solicited symptom collection period. The local and systemic solicited symptoms and their grading were defined in the study protocol (See Supplementary Material).

Clinical blood tests, including full blood count, liver function, renal function and electrolytes, were performed for all volunteers at baseline, as well as days 7, 28 and 112. Volunteers receiving a second intranasal vaccination also had clinical blood tests on days 35 and 42. Additional clinical blood tests at days 14 and 56 were introduced for later volunteers by an amendment. Laboratory adverse events and their grading were defined in the trial protocol (Supplementary Appendix 1).

Blood samples for immunology assays were taken at all visits except days 3, 7 and 35. Nasal mucosal lining fluid (NMLF) samples were collected using a synthetic absorptive matrix (SAM) strip, as previously described, at all timepoints except days 1, 3 and 35.^39^

The study took place in the context of the UK's national rollout of intramuscular COVID-19 vaccines and widespread community transmission of SARS-CoV-2 in the local area. We wished to balance the scientific value of the study data with the desire to avoid participants being disadvantaged by delay in receiving a licensed vaccine. We therefore discouraged participants from receiving non-study intramuscular COVID-19 vaccines until at least 28 days after intranasal vaccination (including the second vaccination, for those who received it), adopted a neutral stance from 28 to 56 days after vaccination, and encouraged participants to receive IM vaccination as soon as possible after day 56.

SARS-CoV-2 testing was not carried out within the study, but participants were advised to seek prompt testing in accordance with the guidelines for the free national SARS-CoV-2 testing programme (including in the event of any SARS-CoV-2 symptoms). History of SARS-CoV-2 infection was solicited at all follow-up visits.

### Immunological assays

Immunological methods are fully described in the Supplementary Methods, including details of samples and data from outside the current study which were used as comparators. In brief, multiplex electrochemiluminescence antibody-binding assays were performed (Meso Scale Discovery, Gaithersburg, MD, USA). Anti-S IgA and IgG, and total IgA, were quantified in NMLF samples. Anti-S IgA and IgG and anti-nucleocapsid IgG were quantified in serum samples. *Ex vivo* interferon-γ ELISpot was performed, using freshly isolated peripheral blood mononuclear cells (PBMCs), as previously described.^31^ Purely descriptive analysis, with no statistical inference testing, was specified by the protocol.

### Statistics

Electronic data capture and clinical data management was carried out using REDCap. Microsoft Excel 2016 was used for tabulation and graphical analysis of safety data exported from REDCap.

**Figure 1:**
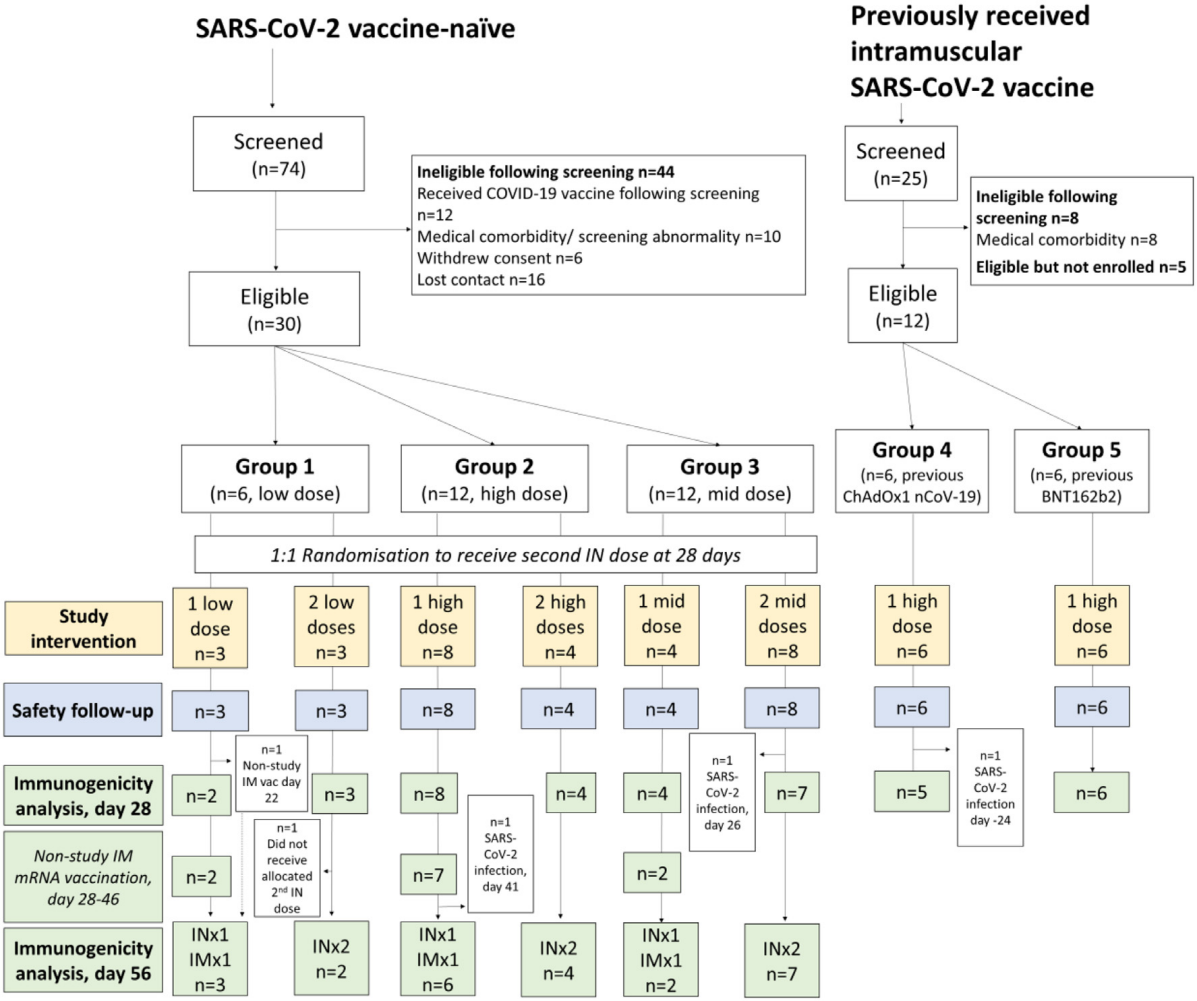
CONSORT flow diagram of study design and volunteer recruitment. CONSORT flow diagram showing recruitment, allocation, and disposition of participants within this trial. Safety follow-up of all enrolled participants was completed, to day 112. After documented SARS-CoV-2 infection, individuals were excluded from categorisation as responders or non-responders to vaccination as indicated, but samples collected after infection are included (denoted by distinct symbols) in graphical representations of immunological data.

Purely descriptive analysis, with no statistical inference testing, was specified by the protocol. Selection of the main timepoints of interest for each immunological parameter, inclusion / exclusion of volunteers from each immunological analysis on the basis of history of SARS-CoV-2 infection or non-study IM vaccination (as detailed in Figure 1), and the definition of detectable mucosal antibody response to vaccination (as tabulated in Supplementary Table 7 i.e. >3-fold-change in the total IgA-normalised value from baseline [FCTIN]) were all *post hoc.*

A planned analysis of geometric mean mucosal antibody concentrations was not performed in view of the substantial number of individuals lacking detectable responses.

### Role of funders

Funded by AstraZeneca and the NIHR Oxford Biomedical Research Centre. ADD, AVSH and KJE are Jenner Investigators. ADD holds a Wellcome Trust fellowship (220679/Z/20/Z). KJE is supported by a Fellowship from the Calleva Foundation. The views expressed are those of the authors and not necessarily those of the NHS, the NIHR or the Department of Health.

The study was proposed and sponsored by the University of Oxford and performed in collaboration with the main funder, AstraZeneca. The academic authors led study design, most data collection, analysis and writing. AstraZeneca authors arranged subcontracting of antibody assays and provided input into design, analysis and the report. The academic authors take responsibility for the conduct and reporting of the trial, and all authors agreed to manuscript submission for publication.

## Results

### Enrolment

SARS-CoV-2 vaccine naïve participants were enrolled into groups 1 – 3 between 1 April and 23 August 2021, with follow-up completed by 13 December 2021 (Figure 1). Following reports of thrombosis with thrombocytopenia syndrome (TTS) in recipients of intramuscularly-administered adenovirus-vectored vaccines enrolment was paused on 8 April 2021. Enrolment resumed on 6 May after discussion with the DSMB and protocol amendment to implement additional safety measures (full details are provided in Supplementary Methods).

Enrolment of group 1 was complete and 12 volunteers had been enrolled in each of groups 2 and 3 before recruitment into these groups was terminated early (due to the progress of the intramuscular vaccination campaign in the local area). At this point eight mid dose recipients (from group 3) and four high dose recipients (from group 2) had been randomised to receive a second intranasal vaccination.

Following a further protocol amendment to add groups 4 and 5, previously SARS-CoV-2 vaccinated participants were enrolled into these groups between 27 October and 4 November 2021, with follow-up completed by 24 February 2022.

Baseline characteristics of the participants in each group are reported in Table 1.

**Table 1:** Baseline characteristics of trial participants.

	SARS-CoV-2 vaccine naïve			Previously received IM SARS-CoV-2 vaccination		
	Low dose (Group 1) 5 × 10^9^vp ChAdOx1 nCOV-19 IN	Mid dose (Group 3) 2 × 10^10^vp ChAdOx1 nCOV-19 IN	High dose (Group 2) 5 × 10^10^vp ChAdOx1 nCoV-19 IN	Group 4 Previous ChAdOx1 nCoV-19 IM	Group 5 Previous BNT162b2 IM	All groups
***n=***	6	12	12	6	6	42
***Gender***						
**Female**	2	6	6	4	4	22
**Male**	4	6	6	2	2	20
***Age***						
**Median (IQR)**	34 (32-34)	32 (31-34)	34 (32-36)	52 (49-53)	37 (36-39)	34 (32-37)
***Ethnicity***						
**Asian or Asian British**	1	2	3	1	0	7
**Mixed**	0	1	1	0	0	2
**White - British**	1	4	7	4	6	22
**White - Other**	4	5	1	1	0	11
***Infection and prior vaccination status***						
**Reported COVID-19 infection before enrolment**	0	0	0	2	0	2
**Serology suggesting prior asymptomatic infection (see text)**	0	0	0	1	0	1
**Days since second IM vaccination Median (IQR)**	N/A	N/A	N/A	142 (118-164)	109 (107-115)	N/A

Differences in the target populations and timing of administration for ChAdOx1 nCoV-19 and BNT162b2 in the local area resulted in a degree of imbalance in the age ranges and time since IM vaccination in groups 4 and 5. IQR: interquartile range. Supplementary Table 1 provides a line listing of baseline characteristics, including information on allocation to the subgroups receiving one or two intranasal doses.

Two participants in group 4 reported proven, symptomatic SARS-CoV-2 infection, 24 and 301 days before enrolment. A third participant in group 4 had serological evidence of possible previous SARS-CoV-2 infection. All other participants denied previous symptomatic infection and were seronegative for anti-nucleocapsid IgG at enrolment.

### Safety and clinical follow-up

**Figure 2:**
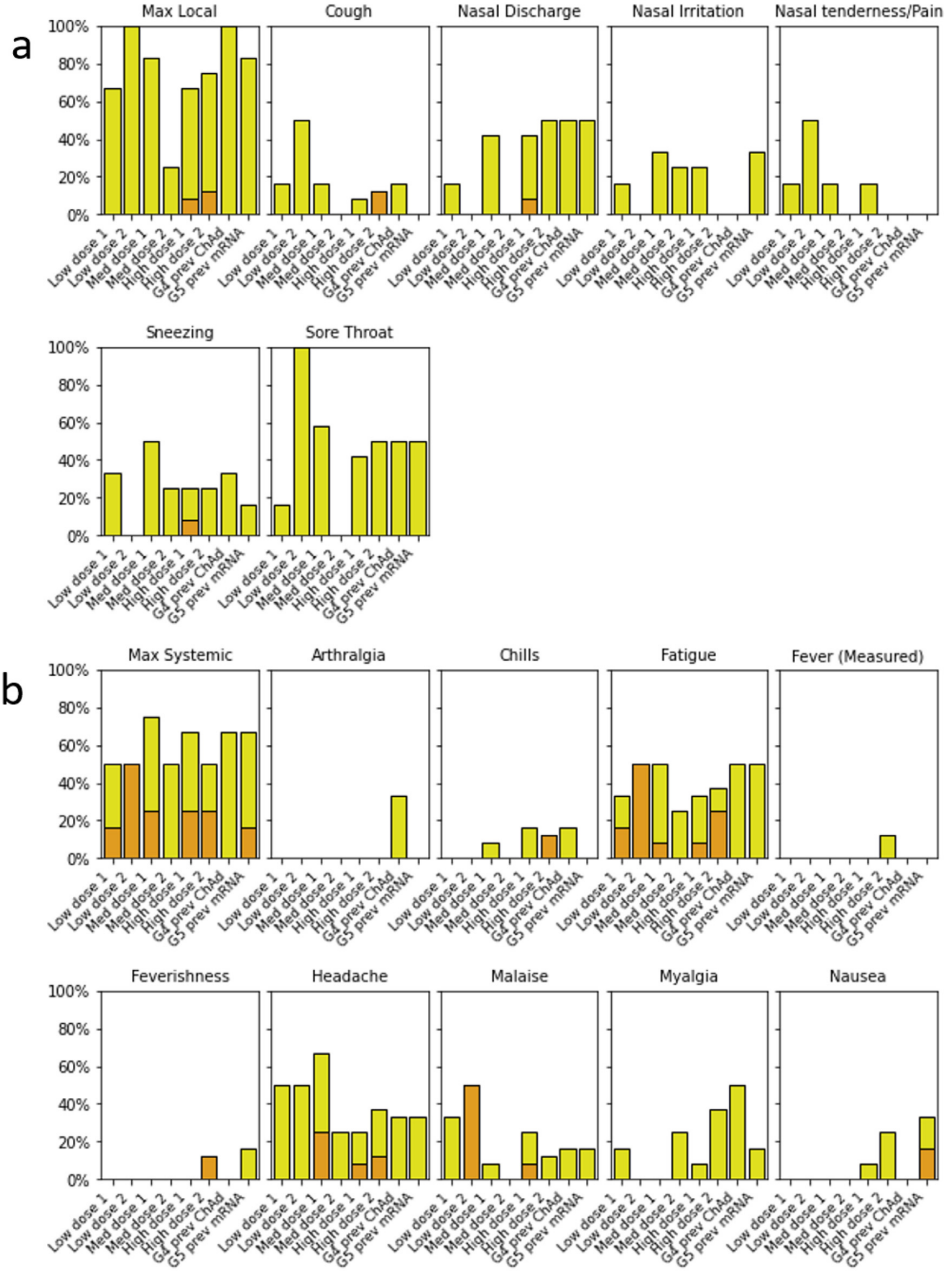
Solicited adverse events following vaccination with intranasal ChAdOx1 nCoV-19. For each of the individual solicited local (panel a) and systemic (panel b) reactions, the maximum severity reported by each volunteer over the seven days after vaccination is shown, broken down by study group and, for groups 1-3, vaccination number (dose 1 = first IN dose, dose 2 = second IN dose). In addition, to provide a global view of reactogenicity, the highest-graded of all local and all systemic reactions is shown for each volunteer. Yellow shading represents grade 1 (mild) events, orange shading represents grade 2 (moderate) events. Denominators were as shown in Figure 1.

Solicited local and systemic reactions in all groups were predominantly mild (grade 1), following both first and second IN vaccinations. Occasional moderate (grade 2) reactions were reported (Figure 2 and Supplementary Figure 1). The most frequent solicited adverse reactions were sore throat (52%), nasal discharge (45%), headache (48%) and fatigue (48%). There was no obvious relationship between the frequency or severity of solicited adverse events and dose level, first versus second IN vaccination, or previous receipt of IM COVID-19 vaccines.

Supplementary Tables 2–3 provide a complete listing of recorded unsolicited adverse events. Supplementary Table 4 provides additional information regarding three non-serious adverse events of note: grade 3 (severe) chest pain without identified cause; grade 3 diplopia due to decompensation of a pre-existing esodeviation (an adverse event of special interest but assessed as unlikely to be related to intranasal vaccination); and grade 1 (mild) transient anosmia 10 days after vaccination, for which the investigators’ assessment was that the most likely cause was intercurrent upper respiratory tract infection, although relatedness to intranasal vaccination could not be excluded.

Supplementary Table 5 provides a complete listing of laboratory adverse events, none of which were assessed to be of clinical significance.

No serious adverse events occurred during the trial.

One participant reported pregnancy at day 112 after vaccination, and remains well and under follow up at the time of writing.

One protocol deviation was reported to MHRA and deemed to be a serious breach of GCP, on grounds of potential risk to volunteers: clinical haematology and biochemistry assays for 16 volunteers were not processed per protocol at day 14 after vaccination. There was no resulting harm.

Although the study was not designed to assess efficacy of IN vaccination against infection, instances of SARS-CoV-2 infection were recorded both because they denoted failure of vaccination to protect an individual from infection, and because they might confound the measurement of vaccine-induced anti-S responses. Seven individuals reported SARS-CoV-2 infection after IN vaccination. Details of these cases are shown in Supplementary Table 6. Of these seven, six showed anti-nucleocapsid seroconversion at day 112 (the exception being an individual infected close to the end of follow-up, at day 102). Anti-nucleocapsid IgG seroconversion was not observed in any of the 32 participants who were anti-nucleocapsid seronegative at baseline and denied subsequent symptomatic infection.

### Mucosal antibody responses

The main immunological objective of the study (and protocol-specified secondary objective) was to assess anti-S mucosal antibody responses to vaccination.

We used a previously-described technique to sample NMLF,^39^ and then measured antibodies binding to SARS-CoV-2 spike protein in both NMLF and serum. NMLF and serum from ten individuals with documented histories of SARS-CoV-2 infection were assayed similarly.

We report three metrics of anti-S IgA and IgG responses: firstly, the absolute values from the antibody binding assays, uncorrected for sample quality (henceforth ‘absolute values’); secondly, the values normalized for the total IgA content of the NMLF sample (henceforth ‘total IgA normalized’, or TIN); and thirdly, the fold-change in the TIN values from an individual's TIN result on that assay at enrolment (henceforth FCTIN). Complete data for each of these metrics, and for total IgA itself, are presented in Supplementary Figure 5–7.

**Figure 3:**
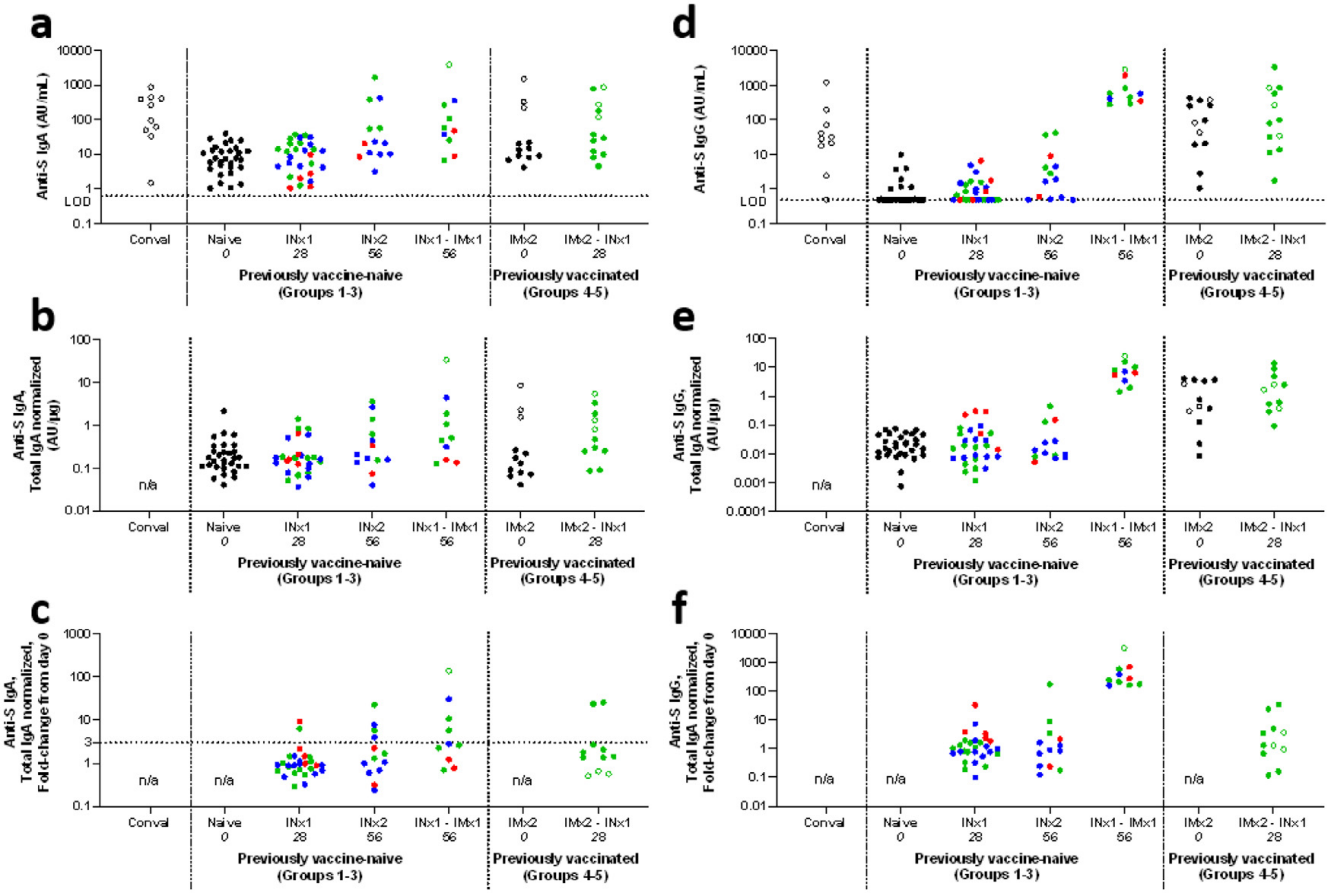
Mucosal antibody responses. Summary of anti-S IgA (panels a-c) and IgG (panels d-f) responses in nasal mucosal samples, measured by electrochemiluminescence assay. Panels a and d show absolute responses (with horizontal dotted lines labelled LOD indicating assay limits of detection), panels b and e show responses normalized for total IgA (TIN), panels c and f show fold change in TIN values (FCTIN, with horizontal dotted lines indicating the arbitrary cut-off of FCTIN>3 used to define responses to vaccination in Supplementary Table 7). Each point represents a sample from a single individual at a given timepoint, and is the mean of results from technical duplicate assays. Colour represents the dose of IN vaccine administered: black represent no IN vaccination; red represents low dose (group 1); blue represents medium dose (group 3); and green represents high dose (groups 2, 4 and 5). Open symbols represent samples from individuals with evidence of preceding SARS-CoV-2 infection. To facilitate visualisation, selected timepoints are shown, and data is combined from the previously vaccine-naïve groups (groups 1-3) and from the previously vaccinated groups (groups 4-5), as indicated in X-axis label. ‘Conval’ represents samples from 10 convalescent individuals with documented SARS-CoV-2 infection (for further details, see Methods). Dotted vertical lines separate data from convalescent samples, groups 1-3, and groups 4-5. The study day on which each sample was collected is indicated in italics in X-axis labels, and corresponds to the main immunological analysis timepoint for each vaccine regime (see Figure 1). For groups 1-3, data is presented for naïve subjects (at enrolment, day 0), after a single IN vaccination (‘INx1’, day 28), and for individuals receiving two IN vaccinations (‘INx2’, day 56) or a non-study IM vaccine after IN vaccination (‘INx1 – IMx1’, day 56). For groups 4-5, data is presented at enrolment (‘IMx2’, day 0), and after IN vaccination (‘IMx2 – INx1’, day 28). Unavailable data is indicated by ‘n/a’. AU/mL indicates arbitrary units per mL. For full antibody kinetics including all timepoints, and with separate presentation of each individual group, see Supplementary Figures 5-6.

In contrast to samples collected after SARS-CoV-2 infection, there was little evidence of mucosal anti-S IgA or IgG responses after a single intranasal vaccination of the vaccine-naïve participants at any dose level (groups 1–3, Figure 3 and Supplementary Table 7). Responses (defined as FCTIN>3) were apparent in 4/13 evaluable participants who received a second IN dose (Supplementary Table 7). Responses after two IN doses only rarely and modestly exceeded median absolute values in convalescent samples (by 9.4-fold for the highest anti-S IgA response, and by 1.4-fold for the highest anti-S IgG response).

Mucosal responses were more consistent among participants in groups 1 – 3 who had received a single IN vaccination (on study day 0) followed by a single non-study IM mRNA vaccination (‘IN-IM’ vaccination). In samples collected on study day 56, after a median interval since IM vaccination of 25 days (range 10 – 34 days), anti-S responses (defined as FCTIN>3) were detectable for IgA in 5/11 participants, and for IgG in 11/11 (Supplementary Table 7). The magnitude of mucosal anti-S IgA levels in these ‘IN-IM’ samples was comparable to convalescent samples (a single participant had anti-S IgA >10-fold above the median of convalescent samples). The magnitude of anti-S IgG levels in ‘IN-IM’ samples typically exceeded those in convalescent samples (14-fold higher median absolute value, Figure 3a).

Among participants in groups 4 and 5, who had received two non-study IM vaccinations a median of 116 days before enrolment (range 105 – 294 days), baseline mucosal anti-S IgA responses appeared indistinguishable from those in vaccine-naïve individuals, with the exception of the three individuals in group 4 whose baseline serology was suggestive of prior SARS-CoV-2 exposure (Figure 3). In contrast, mucosal anti-S IgG responses were detectable in the same baseline samples, with magnitude similar to that in the convalescent samples. Following IN vaccination of these participants, boosting of mucosal anti-S IgA and IgG was detectable in a minority of participants (Figure 3 and Supplementary Table 7).

### Systemic immune responses

**Figure 4:**
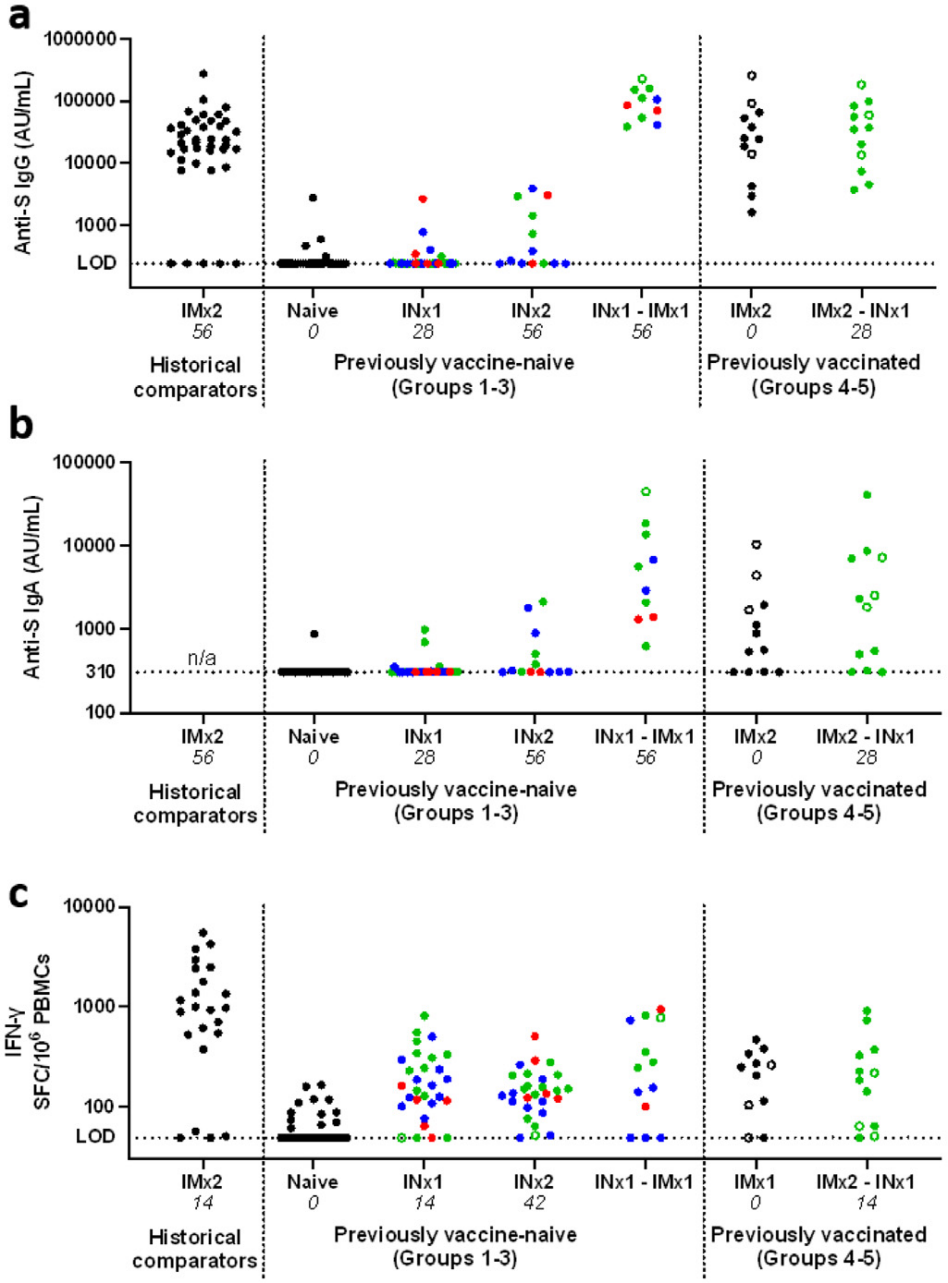
Systemic antibody and cellular responses. Panels a-b show summaries of anti-S IgG (panel a) and IgA (panel b) responses in serum samples, measured by electrochemiluminescence assay. Results from 39 recipients of two IM doses of 5 × 10^10^ VP of ChAdOx1 nCoV-19 are shown as a comparator data set (these individuals had received two doses with a 28 day interval, and samples were collected after a further 28 days). Each point represents a sample from a single individual at a given timepoint, and is the mean of results from technical duplicate assays. Colour represents the dose of IN vaccine administered: black represent no IN vaccination; red represents low dose (group 1); blue represents medium dose (group 3); and green represents high dose (groups 2, 4 and 5). Open symbols represent samples from individuals with evidence of preceding SARS-CoV-2 infection. To facilitate visualisation, selected timepoints are shown, and data is combined from the previously vaccine-naïve groups (groups 1-3) and from the previously vaccinated groups (groups 4-5), as indicated in X-axis label. The study day on which each sample was collected is indicated in italics in X-axis labels, and corresponds to the main immunological analysis timepoint for each vaccine regime (see Figure 1). For groups 1-3, data is presented for naïve subjects (at enrolment, day 0), after a single IN vaccination (‘INx1’, day 28), and for individuals receiving two IN vaccinations (‘INx2’, day 56) or a non-study IM vaccine after IN vaccination (‘INx1 – IMx1’, day 56). For groups 4-5, data is presented at enrolment (‘IMx2’, day 0), and after IN vaccination (‘IMx2 – INx1’, day 28). Unavailable data is indicated by ‘n/a’. AU/mL indicates arbitrary units per mL. Panel c shows peripheral blood mononuclear cell IFN-γ ELISpot results for each group at days 0 and 14 similarly, with results from 23 recipients of two IM doses. For full systemic response kinetics, including all timepoints and with separate presentation of each individual group, see Supplementary Figures 8-9.

A minority of participants had detectable serum anti-S IgG and/or IgA responses 28 days after either a first or second IN vaccination (Figure 4a-b, Supplementary Figure 8 and Supplementary Table 7). These responses were weaker than those seen in participants who received a non-study IM vaccine after IN vaccination. They were also weaker than typical responses to two intramuscular vaccinations, either in samples collected 28 days after a second dose of ChAdOx1 nCoV-19 in another study,^40^ or in baseline samples from group 4–5 participants in the current study (who had received 2x IM vaccines at least 105 days before enrolment).

Most participants had detectable systemic antigen-specific T-cell responses, as measured by peripheral blood mononuclear cell (PBMC) interferon-γ (IFN-γ) ELISpot (Figure 4c and Supplementary Figure 9). Fourteen days after a single IN dose a median of 161 antigen-specific IFN-γ spot forming cells per million PBMCs were detectable, a sixth of the median response at the same timepoint after a single IM dose of ChAdOx1 nCoV-19.

## Discussion

The results reported here show an acceptable safety and tolerability profile of intranasal ChAdOx1 nCoV-19, but relatively weak and inconsistent measured immune responses.

Our main goal when designing the study was pragmatic: to guide a decision on whether to perform a further and larger study of IN ChAdOx1 nCoV-19, using the only formulation/device combination which we felt offered a prospect of rapid deployment during the peak period of the COVID-19 pandemic. We believe a candidate IN vaccine may need to fulfil one of the following criteria in a large proportion of volunteers in a small clinical study to warrant late-stage clinical development: mucosal antibody responses exceeding those induced by SARS-CoV-2 infection; *or* systemic immune responses (ideally neutralizing antibodies) equivalent to those induced by an efficacious licensed IM SARS-CoV-2 vaccine; *or* protection in a SARS-CoV-2 controlled human infection model (CHIM).

Our mucosal and systemic immunological data shows that IN ChAdOx1 nCoV-19 did not meet either the first or the second of these criteria (those relating to mucosal & systemic responses) when administered to vaccine-naïve participants, and did not achieve a clear boosting effect upon these parameters when administered to previously vaccinated participants. Nonetheless, some intranasally-vaccinated volunteers attained mucosal anti-S IgA levels comparable to convalescent patients. This contrasts with a lack of mucosal IgA induction in baseline-seronegative individuals by intramuscular ChAdOx1 nCoV-19 (Kelly, EJ, unpublished). Along with a recent report of detectable nasopharyngeal IgA responses in a minority of recipients of an influenza-vectored SARS-CoV-2 vaccine,^2^ this is one of the first demonstrations of such immunogenicity by a mucosal SARS-CoV-2 vaccine.

We cannot rule out the possibility that IN vaccination could achieve protection in a CHIM study or field efficacy study. Infection of 7/42 participants within 16 weeks of follow-up is however discouraging for the prospect of robust and durable protection by this product delivered using the device used in this study, even though the infecting viruses are likely to have been antigenically distinct from the Wuhan-strain-based vaccine antigen (Supplementary Table 6).^32^ No infections were seen in participants who had received two doses of IN vaccination, but both mucosal and systemic antibody responses measured in INx2 recipients were typically weaker than those in the INx1 – IMx1 and IMx2 – INx1 groups, within which infections were recorded.

The study has a number of limitations. The participant numbers in each group were small, and ability to compare the immunogenicity of IN and IM vaccination is limited by lack of a within-study IM vaccination group. As the study was performed in the context of high levels of community SARS-CoV-2 transmission and a rapidly progressing rollout of intramuscular vaccination, it was necessary to permit volunteers to receive non-study IM vaccination from 28 days after their final IN vaccination. This limited the period over which immunological responses attributable purely to IN vaccination could be followed, but did allow the collection of additional data regarding immune responses in recipients of IM vaccination after an IN ‘prime’.

The study did not incorporate a placebo group, and all assessments were unblinded. This was consistent with our assessment that the study's objectives were unlikely to be compromised by any bias resulting from lack of blinding, is common practice for Phase I vaccine trials at our centre, and avoided exposing placebo recipients to risk of infection due to delay in intramuscular vaccination.

Our immunological analysis was focused upon our pragmatically-defined objectives, as outlined above. We considered measuring virus neutralization by antibody in NMLF but, in view of the poor binding antibody responses, felt that this data would be unlikely to alter our judgment that immunogenicity was insufficient to motivate further development. We have not attempted to characterise mucosal cellular immune responses, partly because we felt it would be challenging to interpret measured responses to inform the go / no-go decision regarding further development. More detailed characterisation of the immune responses to mucosal vaccination remains desirable.

In previous clinical trials of IN and oral replication-defective adenovirus-vectored vaccination against influenza and respiratory syncytial virus, immunogenicity has been variable^30^^,^^41^ (also NCT03232567 and NCT00755703), with little assessment of the induced mucosal responses. In contrast, in non-human primate (NHP) studies of IN adenovirus-vectored SARS-CoV-2 vaccines, robust mucosal antibody responses and systemic responses have both been observed before challenge.^26^^,^^27^

There are a number of possible reasons for the discrepancy between our results and pre-clinical data, each of which suggests possibilities for future enhancement of the performance of IN adenovirus-vectored vaccines.

It is possible that the ChAdOx1 vector, which is derived from a simian adenovirus serotype, may have poor infectivity for human respiratory epithelium, resulting in low levels of expression of the encoded antigen. Previous *in vitro* studies of ChAdOx1 provide a degree of support for this possibility.^42^ Other studies suggest that immunogenicity of mucosally-delivered adenovirus vectors may be limited by low expression of host receptors for adenovirus entry on the apical surfaces of mucosal epithelium and professional antigen presenting cells, and that this problem may be overcome by engineering vectors to achieve broader tropism.^43^ Mucosal adjuvants might stimulate stronger immune responses to a given level of antigen expression, but require care regarding potential for adverse reactions.^25^^,^^44^

The maximum dose we could administer in this study was limited to 5 × 10^10^ VP by the concentration of the available vaccine (c. 1 × 10^11^ VP/mL). In our NHP study of IN vaccination, we administered a dose which was 5- to 20-fold higher, per kilogram of body weight.^26^^,^^27^ The lack of dose-limiting reactogenicity suggests scope for further dose escalation and for other measures to enhance epithelial transduction by the adenovirus, which could include administration of vaccine at higher concentrations, or the use of excipients such as viscosity modifiers.

We used the same delivery device as was used in the NHP study of IN ChAdOx1 nCoV-19, but anatomical differences and sedation of NHPs during IN vaccination may have resulted in different patterns of vaccine deposition. We did not characterise biodistribution of the vaccine in either the NHP study or this clinical study. Another device may achieve improved upper airway residence. Alternatively, delivery of other adenovirus-vectored vaccines to the lower airways by nebulization has been reported to achieve good immunogenicity.^4^^,^^45^^,^^46^ A study of nebulized ChAdOx1 nCoV-19 is ongoing (NCT05007275), but nebulization may be less practical than IN delivery for mass vaccination.

Despite an acceptable safety profile, the immunogenicity of IN ChAdOx1 nCoV-19 in the current study was insufficient to warrant further clinical development of the current formulation / device combination. There are a number of possibilities to improve the immunogenicity of IN adenovirus-vectored vaccines, and results of other clinical trials are awaited. Development of safe, immunogenic and protective ‘platform technologies’ for needle-free vaccination remains a priority both for the response to COVID-19 and more widely.

## Contributors

Conceptualisation: TV, JAG, AVSH, ADD

Funding acquisition: TV, AVSH, ADD

Project administration: AJR

Supervision: MM, AJR, EJK, JAG, IP, TL, KJE, ADD

Investigation: All

Data curation and verification: MM, AJR, JA, DJ, IT, KJE, ADD

Formal analysis: MM, AJR, DJ, KJE, ADD

Visualisation: MM, AJR, DJ, ADD

Writing - original draft: MM, AJR, KJE, ADD

Writing - review & editing: All

All authors read and approved the final version of the manuscript.

MM and DJ verified the clinical data. AJR, JA and KJE or AJR and IT verified the immunological data.

## Data sharing statement

Anonymised participant data and additional documentation relating to the trial (such as informed consent forms) is available upon request directed to the corresponding author, from the time of publication of this report. Proposals will be reviewed and approved by the sponsor, investigator, and collaborators on the basis of scientific merit. After approval of a proposal, data can be shared through a secure online platform after signing a data access agreement. All data will be made available for a minimum of 5 years from the end of the trial.

## Declaration of interests

Oxford University has entered into a partnership with AstraZeneca to develop ChAdOx1 nCoV-19. AJR and KE may receive royalties arising from the University of Oxford/AstraZeneca COVID-19 vaccine. TL is named as an inventor on a patent application covering this SARS-CoV-2 vaccine and was previously a consultant to Vaccitech on an unrelated project. AVSH is a cofounder of and former consultant to Vaccitech is named as an inventor on a patent covering design and use of ChAdOx1-vectored vaccines (PCT/GB2012/000467), and may receive royalties arising for the University of Oxford/AstraZeneca COVID-19 vaccine. DW, EJK, TV, and JAG are current employees of AstraZeneca and hold or may hold AstraZeneca stock. ADD reports grants and personal fees from AstraZeneca outside of the submitted work, is a named inventor on patent applications relating the chimpanzee adenovirus platform technology and manufacturing, and may receive royalties arising from the University of Oxford/AstraZeneca COVID-19 vaccine. All other authors declare no competing interests.

## Appendix Supplementary materials

Image, application 1

Image, application 2
