# Supplementary material for: Tolerability and immunogenicity of an intranasally-administered adenovirus-vectored COVID-19 vaccine: An open-label partially-randomised ascending dose phase I trial
Source: eBioMedicine. 2022 Oct 10;85:104298. doi: 10.1016/j.ebiom.2022.104298 (PMC9550199; doi:10.1016/j.ebiom.2022.104298)
Supplement: Supplementary file 1 [file mmc1.docx]

# Supplementary Methods

## Antibody binding assay

Multiplex electrochemiluminescence serology assays were performed by Meso Scale Discovery Inc. (MSD).

SAM strips were stored at ≤–70⁰C prior to the elution of nasal mucosal lining fluid. Once thawed, the SAM was removed from the collection tube using forceps. The nasal mucosal lining fluid (NMLF) was eluted by incubating the SAM with 330 µL of Diluent 100 (MSD, catalogue number R50AA) for 5–10 minutes at room temperature. Samples were centrifuged at 16,000xg for 15 mins at room temperature and collected via Corning Spin-X centrifuge tube filters without membrane (Corning 9301) into Corning 2 mL polypropylene microcentrifuge tubes (Corning 3213). Samples were stored at ≤–70⁰C prior to analysis.

Serum and nasal mucosal lining fluid samples were evaluated for the presence of anti-SARS-CoV-2 spike antibodies using SARS-CoV-2 Plate 2 Kit (MSD, N05380A) with IgG (MSD, K15383U) and IgA (MSD, K15385U) detection antibodies. The assay was qualified by MSD, which included determination of the upper and the lower limits of quantification for the assay (ULOQ and LLOQ). Participant samples were pre-diluted 1:10 and 1:100-fold using Diluent 100 solution.

Assay plates were blocked with 150 µL/well of blocking solution A (MSD, R93AA) for 30 minutes at room temperature with shaking. Plates were washed three times with 1x wash buffer (MSD, R61AA) prior to the addition of 50 µL/well of reference standard (MSD, C00ADK), serology controls (MSD, C4381), or diluted samples. Plates were sealed and incubated for 2 hours at room temperature with shaking. Plates were washed three times with 1x wash buffer prior to the addition of 50 µL/well of 1x detection antibody solution (MSD, D21ADF [IgG] and D21ADE [IgA]) for 1 hour at room temperature with shaking. Plates were washed with 1x wash buffer for a final three times and analyzed immediately following the addition of 150 µL/well of MSD GOLD Read Buffer B (MSD, R60AM). Each sample was analysed in duplicate wells.

The sample concentration was determined by back-fitting the electrochemiluminescence signal to the MSD reference standard curve (MSD, C00ADK) and reported in arbitrary units per milliliter (AU/mL). For each sample, the data reported was for the least diluted wells that returned results between the upper and lower limits of quantification.

Total IgA in nasal mucosal lining fluid samples was measured using a human/NHP IgA kit (MSD, K150JJD) and reported as micrograms of IgA per mL of the eluate from the SAM strip. For reference and comparison with samples from within the study, we obtained NMLF samples from ten individuals with recently documented SARS-CoV-2 infection (henceforth ‘convalescent samples’). These samples were obtained from a commercial supplier (BioIVT LLC) and had been collected using SAM strips, as above, under a protocol which had received ethical approval from an institutional review board. The donors were six males and four females, with median age 46 years (range 23 – 81 years), and samples were collected a median of 29 days after the individual had tested positive (range 15 – 59 days). Information regarding the severity of these infections was not available. These samples were assayed in the same way as those from the current study.

Additionally, we obtained historical comparator data on systemic responses to intramuscular (IM) ChAdOx1 nCoV-19 vaccination among participants in the COV002 study (clinicaltrials.gov reference NCT04400838) (1). Individuals in group 5d in that study had received two intramuscular doses of 5x10^10^ VP 28 days apart. Anti-S IgG data at day 56 (28 days after second IM vaccination), measured with the same method used for samples from the current study, was available for 39 individuals from this group. Median age of these participants was 39 (range 19-55), and 20 of the 39 were female.

Serum anti-nucleocapsid IgG seroconversion during the course of the study was defined as either a >10-fold rise from baseline, or a rise above a cut-off previously established using the same assay of serum anti-N IgG (MSD). This had 99% specificity and 85% sensitivity for discrimination of samples collected pre-pandemic *versus* samples collected from recently-infected individuals (2).

## *Ex vivo* interferon-γ ELISPOT

*Ex vivo* interferon-γ ELISpot was performed, as previously described (3), using freshly isolated peripheral blood mononuclear cells (PBMCs) at days 0, 14, 28 and 42. The lower limit of detection for the ELISPOT assay was 48 spot-forming cells (SFC) per million PBMC.

For comparison with responses induced by intramuscular vaccination, we used ELISPOT data from the same COV002 participants used as historical comparators for systemic antibody responses: day 0 and day 14 ELISPOT data were available for 23 individuals.

# Supplementary Figures

## Supplementary Figure 1: Timecourse of local solicited adverse events after first intranasal vaccination

Figure shows the same data as are shown in Figure 2 but broken down by day of occurrence. ‘Not completed’ (pale grey shading) denotes incomplete e-diary data entry by a participant.
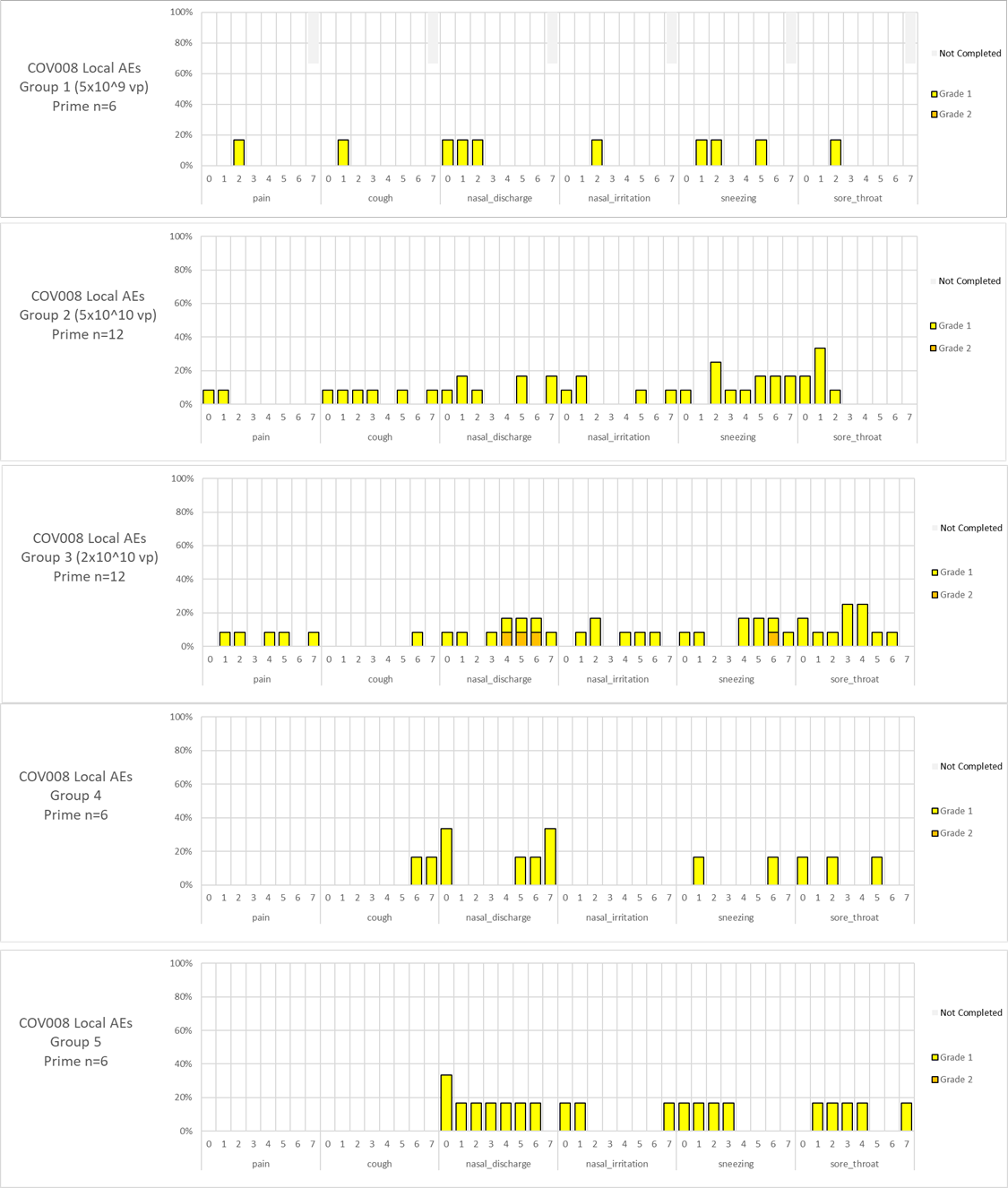


## Supplementary Figure 2: Timecourse of systemic solicited adverse events after first intranasal vaccination

Figure shows the same data as are shown in Figure 2 but broken down by day of occurrence. ‘Not completed’ (pale grey shading) denotes incomplete e-diary data entry by a participant.


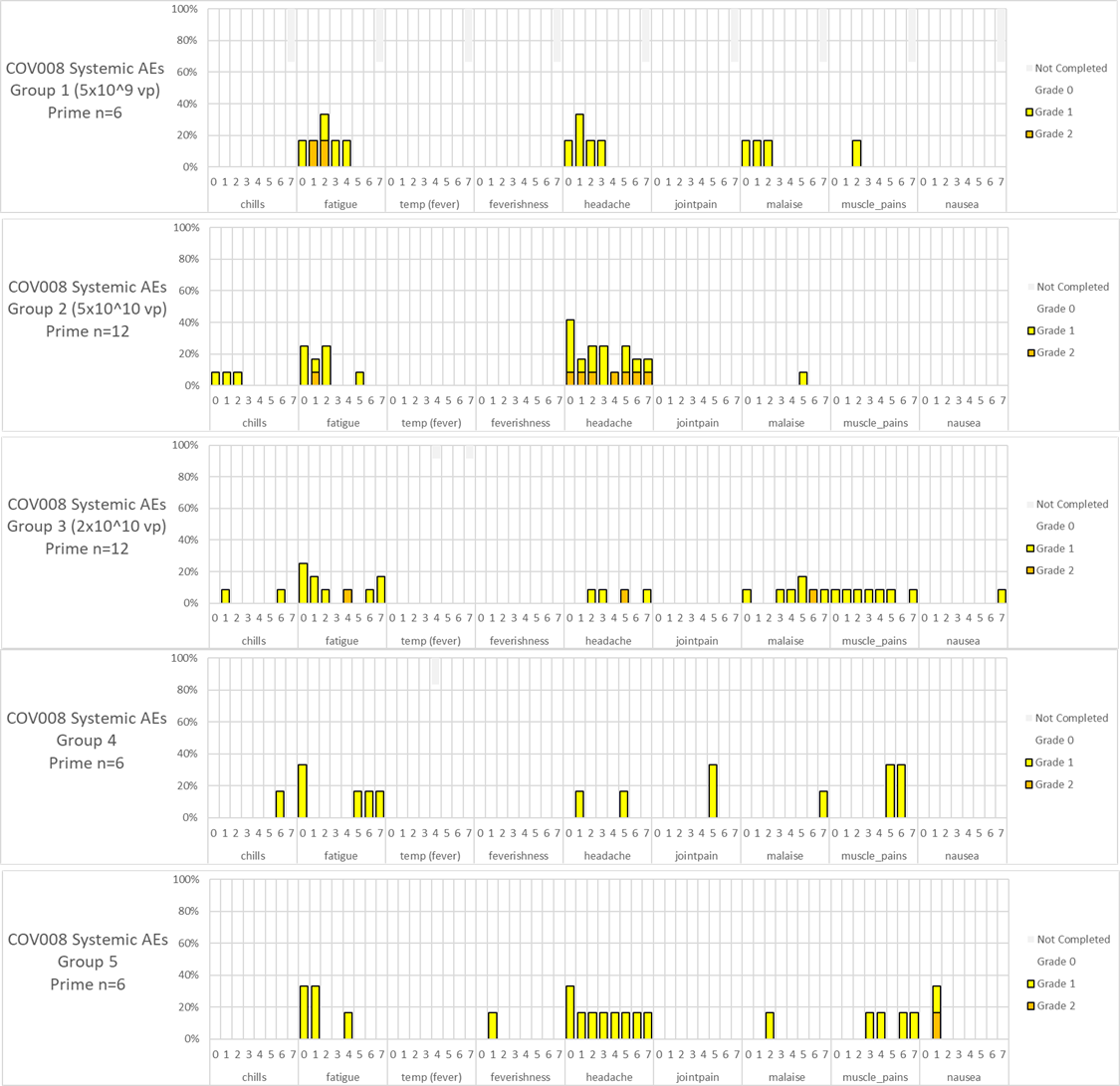


## Supplementary Figure 3: Timecourse of local solicited adverse events after second intranasal vaccination

Figure shows the same data as are shown in Figure 2 but broken down by day of occurrence.


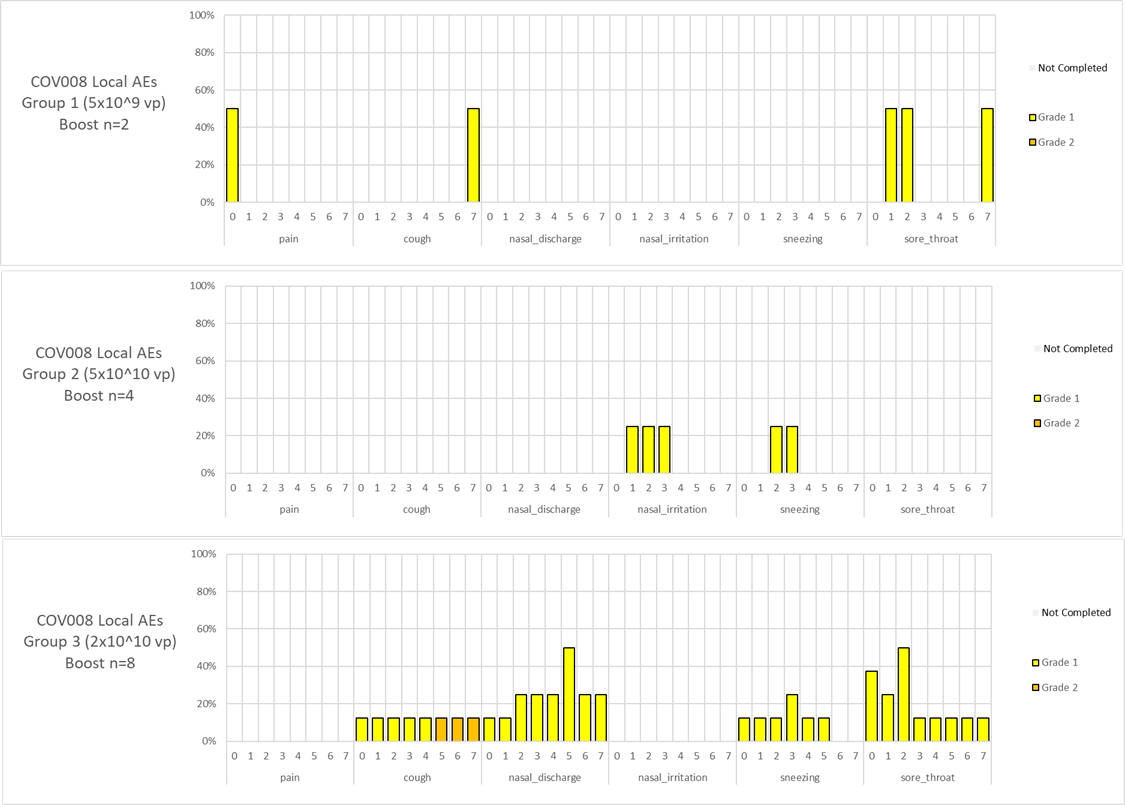


## Supplementary Figure 4: Timecourse of systemic solicited adverse events after second intranasal vaccination

Figure shows the same data as are shown in Figure 2 but broken down by day of occurrence. ‘Not completed’ (pale grey shading) denotes incomplete e-diary data entry by a participant.


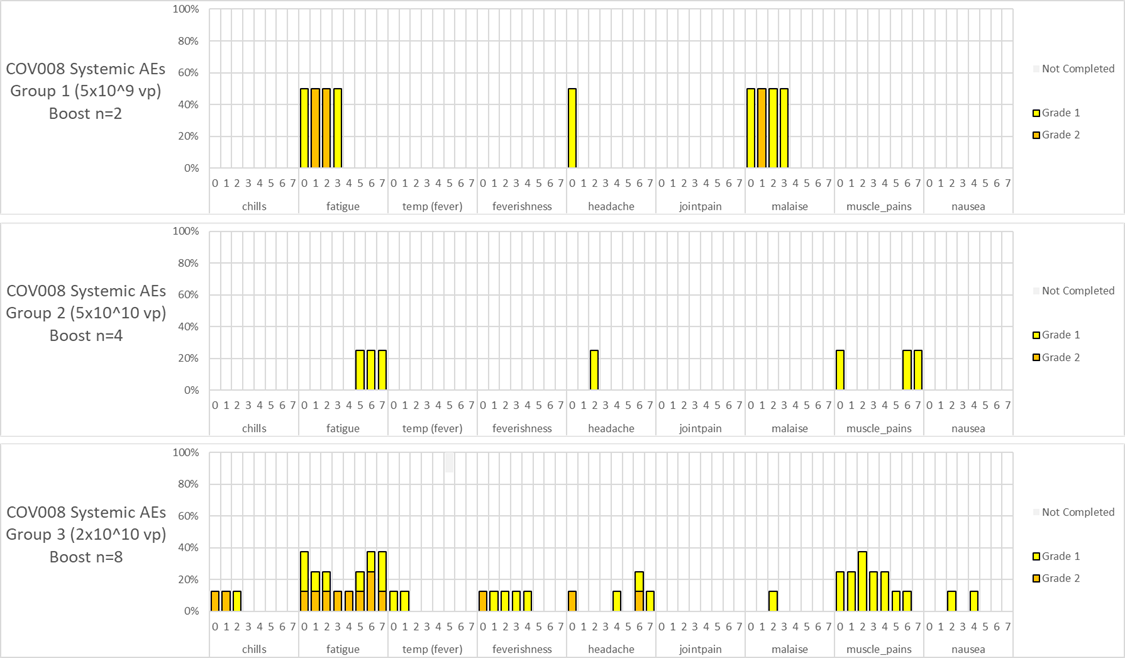


## Supplementary Figure 5: Time kinetic of nasal mucosal anti-S IgA responses

Anti-S IgA responses were measured using nasal samples. The data in row 1 show absolute anti-S IgA responses in arbitrary units (AU) per mL. The data in row 2 shows responses normalised for total IgA in the same nasal sample (TIN). The data in row 3 shows fold change in TIN values (FCTIN).

In columns 1-3, each column represents a study treatment group, with vaccine naïve participants receiving a low dose (red), mid dose (blue) or high dose (green). Open circles and dashed lines represent participants who received one IN dose on day 0. Solid triangles and lines represent participants who received two IN doses, on days 0 and 28. Columns 4 and 5 show participants previously vaccinated with two-doses of ChAdOx1 nCoV-19 (ChAd, purple) or BNT162b2 (mRNA, brown) respectively, all of whom received a single IN dose at day 0.

Grey lines and symbols represent timepoints after the participant received IM vaccination against COVID-19 outside the study (first IM vaccinations for columns 1-3, and boosters for columns 4-5). Solid black symbols represent samples collected after SARS-CoV-2 infection, including one participant in group 4 with baseline serology suggestive of undocumented infection before enrolment, as described in Methods.

## Supplementary Figure 6: Time kinetic of nasal mucosal anti-S IgG responses

Anti-S IgG responses were measured using nasal samples. The data in row 1 show absolute anti-S IgG responses in arbitrary units (AU) per mL. The data in row 2 shows responses normalised for total IgA in the same nasal sample (TIN). The data in row 3 shows fold change in TIN values (FCTIN). LOD (dotted horizontal black line); limit of detection in row 1.

In columns 1-3, each column represents a study treatment group, with vaccine naïve participants receiving a low dose (red), mid dose (blue) or high dose (green). Open circles and dashed lines represent participants who received one IN dose on day 0. Solid triangles and lines represent participants who received two IN doses, on days 0 and 28. Columns 4 and 5 show participants previously vaccinated with two-doses of ChAdOx1 nCoV-19 (ChAd, purple) or BNT162b2 (mRNA, brown) respectively, all of whom received a single IN dose at day 0.

Grey lines and symbols represent timepoints after the participant received IM vaccination against COVID-19 outside the study (first IM vaccinations for columns 1-3, boosters for columns 4-5). Solid black symbols represent samples collected after SARS-CoV-2 infection, including one participant in group 4 with baseline serology suggestive of undocumented infection before enrolment, as described in Methods.

## Supplementary Figure 7: Total IgA concentration in SAM strip eluates

NMLF samples collected on SAM strips were each eluted in 330 μL of diluent, and total IgA concentration in the eluate was measured using an electrochemiluminescence-based assay. The median concentration was 82 μg/mL, indicated by line. The interquartile range was 49-144 μg/mL. One influence on the total IgA concentration in the eluate would be the volume of NMLF collected on the SAM strip. This is unknown and expected to vary between strips, but the elution is estimated to involve c. 10-fold dilution of a typical NMLF sample.

## Supplementary Figure 8: Time kinetic of serum anti-S IgG and IgA responses

Anti-S IgA (top row) and anti-S IgG (bottom row) responses were measured using serum samples. Data is shown as absolute anti-S IgA and IgG responses in arbitrary units (AU) per mL. LOD (dotted horizontal black line); limit of detection.

In columns 1-3, each column represents a study treatment group, with vaccine naïve participants receiving a low dose (red), mid dose (blue) or high dose (green). Open circles and dashed lines represent participants who received one IN dose on day 0. Solid triangles and lines represent participants who received two IN doses, on days 0 and 28. Columns 4 and 5 show participants previously vaccinated with two-doses of ChAdOx1 nCoV-19 (ChAd, purple) or BNT162b2 (mRNA, brown) respectively, all of whom received a single IN dose at day 0.

Grey lines and symbols represent timepoints after the participant received IM vaccination against COVID-19 outside the study. Solid black symbols represent samples collected after SARS-CoV-2 infection, including one participant in group 4 with baseline serology suggestive of undocumented infection before enrolment, as described in Methods.

## Supplementary Figure 9: Time kinetic of serum IFN-γ ELISpot responses

Anti-S IFN-γ ELISpot responses were measured using freshly isolated PBMCs at selected timepoints. Data is shown as spot forming cells (SPCs) per 10^6^ PMBCs. LOD (dotted horizontal black line); limit of detection.

Each of panels 1-3 represents a study treatment group, with vaccine naïve participants receiving a low dose (red), mid dose (blue) or high dose (green). Open circles and dashed lines represent participants who received one IN dose on day 0. Solid triangles and lines represent participants who received two IN doses, on days 0 and 28. Grey lines and symbols represent timepoints after the participant received IM vaccination against COVID-19 outside the study. Panels 4 and 5 show participants previously vaccinated with two-doses of ChAdOx1 nCoV-19 (ChAd, purple) or BNT162b2 (mRNA, brown) respectively, all of whom received a single IN dose at day 0.

Solid black symbols represent samples collected after SARS-CoV-2 infection, including one participant in group 4 with baseline serology suggestive of undocumented infection before enrolment, as described in Methods.

# Supplementary Tables

## Supplementary Table 1: Baseline information

Listing of volunteers. The volunteer marked with an asterisk did not receive the allocated second dose.

| Group | Allocated to single or two IN doses? | Age at screening | Gender | Ethnicity | Days since preceding vaccine dose (groups 4/5) |
| --- | --- | --- | --- | --- | --- |
| 1 (Low) | 1 | 32 | Male | Other |  |
| 1 | 1 | 34 | Female | White - British |  |
| 1 | 1 | 35 | Male | White - Other |  |
| 1 | 2* | 26 | Male | White - Other |  |
| 1 | 2 | 33 | Male | White - Other |  |
| 1 | 2 | 34 | Female | Asian / Asian British | |
| 2 (High) | 1 | 40 | Female | White - Other |  |
| 2 | 1 | 36 | Female | White - British |  |
| 2 | 1 | 34 | Male | White - British |  |
| 2 | 1 | 30 | Male | Asian or Asian British | |
| 2 | 1 | 34 | Female | Asian or Asian British | |
| 2 | 1 | 32 | Female | White - British |  |
| 2 | 1 | 33 | Male | White - British |  |
| 2 | 1 | 36 | Male | White - British |  |
| 2 | 2 | 33 | Female | White - British |  |
| 2 | 2 | 37 | Male | Asian or Asian British | |
| 2 | 2 | 31 | Female | Other |  |
| 2 | 2 | 30 | Male | White - British |  |
| 3 (Mid) | 1 | 32 | Female | White - British |  |
| 3 | 1 | 30 | Female | White - Irish |  |
| 3 | 1 | 31 | Male |  |  |
| 3 | 1 | 31 | Female | White - British |  |
| 3 | 2 | 33 | Male | White - Other |  |
| 3 | 2 | 33 | Male | White - Other |  |
| 3 | 2 | 36 | Female |  |  |
| 3 | 2 | 31 | Male | Mixed - Other |  |
| 3 | 2 | 39 | Male | White - British |  |
| 3 | 2 | 37 | Female | White - Other |  |
| 3 | 2 | 31 | Male | White - British |  |
| 3 | 2 | 32 | Female | White - Other |  |
| 4 | 1 | 41 | Male | White - British | -128 |
| 4 | 1 | 55 | Female | White - British | -166 |
| 4 | 1 | 52 | Female | White - British | -115 |
| 4 | 1 | 52 | Female | White - British | -156 |
| 4 | 1 | 48 | Female | Other | -114 |
| 4 | 1 | 53 | Male | White - Other | -271 |
| 5 | 1 | 36 | Male | White - British | -106 |
| 5 | 1 | 41 | Female | White - British | -294 |
| 5 | 1 | 36 | Female | White - British | -105 |
| 5 | 1 | 37 | Female | White - British | -110 |
| 5 | 1 | 39 | Male | White - British | -117 |
| 5 | 1 | 34 | Female | White - British | -108 |

## Supplementary Table 2: Unsolicited adverse events within 28 days of first study vaccination

Adverse events are listed with MEDDRA system organ class (SOC) and preferred terms (PT). Table indicates number of volunteers reporting each adverse event, with totals indicating the number of volunteers in each group reporting any unsolicited adverse event. All AEs were mild-to-moderate (grades 1-2) in severity, with the exception of two grade 3 AEs described in Supplementary Table 3.

|  |  |  | **Low** | **Mid** | **High** | **Prev ChAd** | **Prev mRNA** | **Total** |
| --- | --- | --- | --- | --- | --- | --- | --- | --- |
|  |  |  | **(n=6)** | **(n=12)** | **(n=12)** | **(n=6)** | **(n=6)** | **(n=42)** |
| **Possibly, Probably or Definitely Related** | | | **2** | **7** | **6** | **3** | **3** | **21** |
|  | **Ear and labyrinth disorders** | | **—** | **1** | **—** | **—** | **—** | **1** |
|  | | Ear discomfort | — | 1 | — | — | — | 1 |
|  | |  |  |  |  |  |  |  |
|  | **Eye disorders** | | **1** | **—** | **—** | **—** | **—** | **1** |
|  | | Conjunctival haemorrhage | 1 | — | — | — | — | 1 |
|  | |  |  |  |  |  |  |  |
|  | **Gastrointestinal disorders** | | **—** | **1** | **—** | **—** | **—** | **1** |
|  | | Haematochezia | — | 1 | — | — | — | 1 |
|  | |  |  |  |  |  |  |  |
|  | **General disorders and administration site conditions** | | **1** | **2** | **—** | **1** | **1** | **5** |
|  | | Chest pain | — | — | — | 1 | — | 1 |
|  | | Exercise tolerance decreased | — | 1 | — | — | — | 1 |
|  | | Fatigue | — | 1 | — | — | — | 1 |
|  | | Influenza like illness | — | 1 | — | — | — | 1 |
|  | | Malaise | — | — | — | — | 1 | 1 |
|  | | Pyrexia | 1 | — | — | — | — | 1 |
|  | |  |  |  |  |  |  |  |
|  | **Infections and infestations** | | **—** | **1** | **—** | **2** | **1** | **4** |
|  | | Nasopharyngitis | — | — | — | — | 1 | 1 |
|  | | Rhinitis | — | 1 | — | 1 | — | 2 |
|  | | Tonsillitis | — | — | — | 1 | — | 1 |
|  | |  |  |  |  |  |  |  |
|  | **Nervous system disorders** | | **1** | **1** | **3** | **—** | **—** | **5** |
|  | | Anosmia | 1 | — | — | — | — | 1 |
|  | | Dizziness | — | — | 1 | — | — | 1 |
|  | | Headache | — | — | 2 | — | — | 2 |
|  | | Sinus headache | — | 1 | — | — | — | 1 |
|  | |  |  |  |  |  |  |  |
|  | **Respiratory, thoracic and mediastinal disorders** | | **1** | **3** | **3** | **2** | **3** | **12** |
|  | | Cough | — | — | — | 1 | 1 | 2 |
|  | | Dry throat | — | 1 | — | — | — | 1 |
|  | | Epistaxis | — | — | — | — | 1 | 1 |
|  | | Nasal congestion | 1 | — | 1 | 1 | 2 | 5 |
|  | | Oropharyngeal pain | — | 1 | 2 | — | 2 | 5 |
|  | | Rhinorrhoea | 1 | 1 | 1 | — | 1 | 4 |
|  | | Sinus congestion | — | 1 | — | — | — | 1 |

|  | |  |  | |  | |  | |  | |  | |  |
| --- | --- | --- | --- | --- | --- | --- | --- | --- | --- | --- | --- | --- | --- |
|  | |  |  | |  | |  | |  | |  | |  |
| **Unrelated or Unlikely Related** | | | **2** | | **5** | | **3** | | **2** | | **2** | | **14** |
|  | **Eye disorders** | | **—** | | **1** | | **—** | | **—** | | **—** | | **1** |
|  | | Photophobia | — | | 1 | | — | | — | | — | | 1 |
|  | |  |  | |  | |  | |  | |  | |  |
|  | **Gastrointestinal disorders** | | **—** | | **—** | | **—** | | **1** | | **1** | | **2** |
|  | | Abdominal distension | — | | — | | — | | — | | 1 | | 1 |
|  | | Abdominal pain | — | | — | | — | | — | | 1 | | 1 |
|  | | Diarrhoea | — | | — | | — | | 1 | | — | | 1 |
|  | |  |  | |  | |  | |  | |  | |  |
|  | **General disorders and administration site conditions** | | **1** | | **1** | | **1** | | **1** | | **—** | | **4** |
|  | | Fatigue | — | | 1 | | 1 | | 1 | | — | | 3 |
|  | | Vaccination site pain | 1 | | — | | — | | — | | — | | 1 |
|  | |  |  | |  | |  | |  | |  | |  |
|  | **Infections and infestations** | | **—** | | **1** | | **—** | | **—** | | **—** | | **1** |
|  | | Pulpitis dental | — | | 1 | | — | | — | | — | | 1 |
|  | |  |  | |  | |  | |  | |  | |  |
|  | **Injury, poisoning and procedural complications** | | **—** | | **—** | | **1** | | **—** | | **—** | | **1** |
|  | | Sunburn | — | | — | | 1 | | — | | — | | 1 |
|  | |  |  | |  | |  | |  | |  | |  |
|  | **Musculoskeletal and connective tissue disorders** | | **—** | | **2** | | **—** | | **—** | | **—** | | **2** |
|  | | Back pain | — | | 1 | | — | | — | | — | | 1 |
|  | | Muscle spasms | — | | 1 | | — | | — | | — | | 1 |
|  | | Pain in extremity | — | | 1 | | — | | — | | — | | 1 |
|  | |  |  | |  | |  | |  | |  | |  |
|  | **Nervous system disorders** | | **1** | | **3** | | **2** | | **1** | | **2** | | **9** |
|  | | Dizziness | — | | 1 | | — | | — | | — | | 1 |
|  | | Headache | 1 | | 2 | | 2 | | 1 | | 2 | | 8 |
|  | | Sinus headache | — | | 1 | | — | | — | | — | | 1 |
|  | |  |  | |  | |  | |  | |  | |  |
|  | **Reproductive system and breast disorders** | | **—** | | **1** | | **—** | | **—** | | **—** | | **1** |
|  | | Dysmenorrhoea | — | | 1 | | — | | — | | — | | 1 |
|  | |  |  | |  | |  | |  | |  | |  |
|  | **Respiratory, thoracic and mediastinal disorders** | | **—** | | **—** | | **1** | | **—** | | **—** | | **1** |
|  | | Cough | — | | — | | 1 | | — | | — | | 1 |
|  | |  |  | |  | |  | |  | |  | |  |
| **Total** | |  | **2** | **9** | | **7** | | **5** | | **4** | | **27** | |

## Supplementary Table 3: Unsolicited adverse events within 28 days of second study vaccination

Adverse events are listed with MEDDRA system organ class (SOC) and preferred terms (PT). Table indicates number of volunteers reporting each adverse event, with totals indicating the number of volunteers in each group reporting any unsolicited adverse event.

|  |  |  | **Low** | **Mid** | **High** | **Total** |
| --- | --- | --- | --- | --- | --- | --- |
|  |  |  | **(n=3)** | **(n=4)** | **(n=8)** | **(n=15)** |
| **Possibly, Probably or Definitely Related** | | | **1** | **2** | **—** | **3** |
|  | **Nervous system disorders** | | **—** | **2** | **—** | **2** |
|  | | Headache | — | 1 | — | 1 |
|  | | Sinus headache | — | 1 | — | 1 |
|  | |  |  |  |  |  |
|  | **Respiratory, thoracic and mediastinal disorders** | | **1** | **1** | **—** | **2** |
|  | | Cough | 1 | 1 | — | 2 |
|  | | Nasal congestion | — | 1 | — | 1 |
|  | | Oropharyngeal pain | 1 | 1 | — | 2 |
|  | | Rhinorrhoea | 1 | — | — | 1 |
|  | |  |  |  |  |  |
| **Unrelated or Unlikely Related** | |  | **—** | **2** | **2** | **4** |
|  | **General disorders and administration site conditions** | | **—** | **1** | **1** | **2** |
|  | | Fatigue | — | 1 | — | 1 |
|  | | Hangover | — | — | 1 | 1 |
|  | |  |  |  |  |  |
|  | **Musculoskeletal and connective tissue disorders** | | **—** | **1** | **—** | **1** |
|  | | Neck pain | — | 1 | — | 1 |
|  | |  |  |  |  |  |
|  | **Nervous system disorders** | | **—** | **2** | **2** | **4** |
|  | | Headache | — | 2 | 2 | 4 |
|  | |  |  |  |  |  |
|  | **Skin and subcutaneous tissue disorders** | | **—** | **—** | **1** | **1** |
|  | | Rash | — | — | 1 | 1 |
|  | |  |  |  |  |  |
| **Total** |  |  | **1** | **3** | **2** | **6** |

## Supplementary Table 4: Details of selected adverse events of note

Table contains details of the three events in the trial which were either of grade 3 (severe) severity, or constituted an adverse event of special interest.

| **Volunteer details** | **Adverse event** | **Time of onset** | **Grade** | **Serious?** | **Narrative** | **Assessed relatedness to intranasal vaccination** |
| --- | --- | --- | --- | --- | --- | --- |
| 32 year old male, group 1 | Anosmia | Study day 10 | 1 | No | Reported newly reduced sense of smell associated with nasal congestion, nasal discharge and feverishness (self-measured temperature: 37.5). All symptoms had resolved by D14 visit without intervention and was well when reviewed.  A subsequent amendment defined anosmia as an adverse event of special interest (AESI), but this was not an AESI under the definition at time of occurrence. | Possible: temporally and mechanistically plausible, but intercurrent upper respiratory tract infection assessed as more likely cause. |
| 33 year old male, group 3 | Diplopia | Study day 29 | 3 | No | Diplopia developed within 24 hours of receiving a non-study IM COVID-19 vaccine (mRNA-1273, Moderna). The participant had a history of previous strabismus surgery, with ongoing bilateral lateral rectus weakness and neuropsychiatric sequelae following a past head injury. Orthoptic assessment (including Hess chart, 8 days after symptom onset), found no change from a previous assessment (undertaken prior to study enrolment), and the most likely diagnosis was felt to be a decompensated esodeviation due to the systemic response to mRNA-1273. Classified as AESI. | Unlikely: investigators and DSMB in agreement that symptom likely to be attributable to mRNA-1273 and pre-existing condition. |
| 55 year old female, group 4 | Chest pain | Study day 5 | 3 | No | Participant attended emergency department with chest pain. Serum troponin and CT pulmonary angiography were both normal. The pain resolved without sequelae on the same day, and no cause was diagnosed. | Possible: temporally plausible, though no obvious mechanism of causation by intranasal vaccination. |

## Supplementary Table 5: Laboratory adverse events within 56 days of first intranasal vaccination

|  | **Group 1- Low (n=6)** | | | | | **Group 3- Mid (n=12)** | | | | | | **Group 2- High (n=12)** | | | | | | **Group 4- Previous ChAd (n=6)** | | | | **Group 5- Previous mRNA (n=6)** | | | |
| --- | --- | --- | --- | --- | --- | --- | --- | --- | --- | --- | --- | --- | --- | --- | --- | --- | --- | --- | --- | --- | --- | --- | --- | --- | --- |
| **Timepoint (day)** | **7** | **28** | **35** | **42** | **56** | **7** | **14** | **28** | **35** | **42** | **56** | **7** | **14** | **28** | **35** | **42** | **56** | **7** | **14** | **28** | **56** | **7** | **14** | **28** | **56** |
| **Total assessed** | **6** | **6** | **2** | **2** | **5** | **12** | **11** | **12** | **8** | **8** | **12** | **12** | **11** | **12** | **4** | **4** | **12** | **6** | **6** | **6** | **6** | **6** | **6** | **6** | **6** |
| **ALT** |  |  |  |  |  |  |  |  |  |  |  |  |  |  |  |  |  |  |  |  |  |  |  |  |  |
| Grade 1 |  |  |  |  |  |  |  |  |  |  | 1 |  |  |  |  |  |  | 1 | 1 | 1 | 1 |  |  | 1 | 1 |
| **Bilirubin** |  |  |  |  |  |  |  |  |  |  |  |  |  |  |  |  |  |  |  |  |  |  |  |  |  |
| Grade 1 | 2 |  | 1 |  |  |  |  |  |  |  |  |  |  |  |  |  |  |  |  |  |  |  |  |  |  |
| Grade 2 |  | 1 |  |  | 1 |  |  |  |  |  |  |  |  |  |  |  |  |  |  |  |  | 1 |  |  | 1 |
| Grade 3 |  |  |  |  |  |  |  |  |  |  |  |  |  |  |  |  |  |  |  |  |  |  | 1 | 1 |  |
| **Haemoglobin** |  |  |  |  |  |  |  |  |  |  |  |  |  |  |  |  |  |  |  |  |  |  |  |  |  |
| Grade 1 (low) |  |  |  |  |  |  |  |  |  |  |  | 1 | 1 | 1 | 1 | 1 |  |  |  |  |  |  |  |  |  |
| **Lymphocytes** |  |  |  |  |  |  |  |  |  |  |  |  |  |  |  |  |  |  |  |  |  |  |  |  |  |
| Grade 1 (low) |  |  |  |  |  | 2 |  |  |  | 1 | 2 | 1 | 1 | 1 |  |  | 1 |  |  |  |  | 1 | 1 |  | 1 |
| Grade 2 (low) |  |  |  |  |  |  | 1 |  |  |  |  |  |  |  |  |  |  |  |  |  |  |  |  |  |  |
| Grade 3 (low) |  |  |  |  |  |  |  | 1 |  |  |  |  |  |  |  |  |  |  |  |  |  |  |  |  |  |
| **Neutrophils** |  |  |  |  |  |  |  |  |  |  |  |  |  |  |  |  |  |  |  |  |  |  |  |  |  |
| Grade 1 (low) | 1 | 1 | 1 |  |  | 1 |  |  | 1 |  | 1 |  |  | 1 |  |  |  |  |  | 1 |  |  | 2 |  | 1 |
| Grade 2 (low) |  | 1 |  |  | 1 |  |  |  |  |  |  |  |  |  |  |  |  |  |  |  |  |  |  |  |  |
| **Potassium** |  |  |  |  |  |  |  |  |  |  |  |  |  |  |  |  |  |  |  |  |  |  |  |  |  |
| Grade 1 (low) |  | 1 | 1 |  | 1 | 2 |  | 1 |  | 2 | 1 | 3 |  |  |  | 1 | 1 |  |  | 1 |  |  |  |  |  |
| Grade 2 (low) |  |  |  |  |  | 1 |  |  |  |  | 1 |  | 2 | 1 |  |  |  |  |  |  |  |  |  |  |  |
| Grade 3 (low) |  |  |  |  |  | 1 |  |  |  |  | 1 |  |  |  |  |  |  |  |  |  |  |  |  |  |  |
| **Urea** |  |  |  |  |  |  |  |  |  |  |  |  |  |  |  |  |  |  |  |  |  |  |  |  |  |
| Grade 1 |  |  |  |  |  |  | 1 |  |  | 2 | 1 |  |  |  |  |  |  |  |  |  |  |  |  |  |  |
| Grade 2 |  |  |  |  |  |  |  |  |  |  |  |  | 1 |  |  |  | 1 |  |  |  |  |  |  |  |  |
| **WBC** |  |  |  |  |  |  |  |  |  |  |  |  |  |  |  |  |  |  |  |  |  |  |  |  |  |
| Grade 1 (low) |  | 1 |  |  | 1 |  |  |  |  |  |  |  |  | 1 |  |  | 1 |  |  |  |  |  | 1 |  | 1 |
| Grade 1 (high) |  |  |  |  |  |  |  |  | 1 |  |  |  | 1 |  | 1 |  | 1 |  |  |  |  |  |  |  |  |

Notes: *Bilirubin:* one volunteer with a past medical history of Gilbert’s disease was enrolled in group 1. Bilirubin was high at baseline and continued to fluctuate but did not exceed the original level. In addition, a single volunteer in group 5 with persistently high bilirubin following screening was found to have unconjugated hyperbilirubinaemia without signs of liver disease or haemolysis. This was presumed to be undiagnosed Gilbert’s. *ALT:* None of the mild rises in ALT was associated with a rise in bilirubin or signs of liver disease. *Haemoglobin:* a single volunteer in Group 2 had a marginally low haemoglobin level throughout the trial. This remained stable and was not significantly lower than baseline level. All other abnormalities were temporary and asymptomatic, and resolved without intervention. Key. ALT: Alanine transaminase, WBC: White blood cell count.

## Supplementary Table 6: SARS-CoV-2 infections during study

The vaccination histories and temporal relationships of vaccination to infection of the seven individuals who reported symptomatic SARS-CoV-2 infection during the study are tabulated. None required hospitalisation. The viruses responsible were not genotyped, but the dominant SARS-CoV-2 variant in the local area was Delta in July – November 2021, and Omicron from December 2021 – February 2022 (4).

| **Case** | **Group (IN dose level)** | **Vaccines received prior to infection, study and non-study: identity and timing (study day)** | | | | **Date of symptom onset (study day)** |
| --- | --- | --- | --- | --- | --- | --- |
|  |  | **First** | **Second** | **Third** | **Fourth** |  |
| **A** | 2 (high) | IN ChAdOx1  (day 0) | IM BNT162b2  (day 32) | n/a | n/a | 18/7/21  (day 41) |
| **B** | 2 (high) | IN ChAdOx1  (day 0) | IM BNT162b2  (day 31) | n/a | n/a | 4/9/21  (day 87) |
| **C** | 3 (med) | IN ChAdOx1  (day 0) | IM BNT162b2  (day 30) | n/a | n/a | 15/8/21  (day 80) |
| **D** | 3 (med) | IN ChAdOx1  (day 0) | n/a | n/a | n/a | 18/9/21  (day 26) |
| **E** | 5 (high) | IM BNT162b2  (day -162) | IM BNT162b2  (day -106) | IN ChAdOx1  (day 0) | IM BNT162b2  (day 46) | 16/1/22  (day 74) |
| **F** | 5 (high) | IM BNT162b2  (day -166) | IM BNT162b2  (day -110) | IN ChAdOx1  (day 0) | IM BNT162b2  (day 52) | 12/1/22  (day 72) |
| **G** | 5 (high) | IM BNT162b2  (day -170) | IM BNT162b2  (day -117) | IN ChAdOx1  (day 0) | IM BNT162b2  (day 90) | 13/2/22  (day 102) |

## Supplementary Table 7: Tabulation of mucosal responses to vaccination

Table shows number of individuals with FCTIN >3 / number of individuals evaluable for each analyte and timepoint. Eligibility of individuals for immunological analysis at each timepoint was as defined in Methods and shown in Figure 1.

This analysis should be treated with caution as many assay results were close to the limit of detection. The analysis is intended to be sensitive rather than specific for responses, and so may over-estimate the number of true responses to vaccination. Most of the small number of samples with FCTIN>3 from groups 1-3 at day 28 (i.e. after a single IN dose in vaccine-naïve participants) had weak raw antigen-specific responses with relatively little change between timepoints, and change in total IgA between timepoints was a substantial contributor to FCTIN. Both of these factors may raise the probability of FCTIN>3 in these samples representing a ‘false positive’ response. Responses in day 56 and group 4/5 samples were generally clear-cut, as shown in Figure 3 and Supplementary Figures 5 and 6.

|  |  |  | **Previously SARS-CoV-2 vaccine naïve** | | | | **Previously SARS-CoV-2 vaccinated** | | |
| --- | --- | --- | --- | --- | --- | --- | --- | --- | --- |
| **Regime** | **Study day** | **Analyte** | **Low**  **dose**  **(G1)** | **Mid**  **dose**  **(G3)** | **High**  **dose**  **(G2)** | ***TOTAL*** | **Previous ChAdOx1**  **(G4)** | **Previous BNT162b2**  **(G5)** | ***TOTAL*** |
| **INx1** | 28 | SAM IgA | 1/5 | 0/11 | 1/12 | *2/28* | 0/5 | 2/6 | *2/11* |
| **INx1** | 28 | SAM IgG | 2/5 | 1/11 | 0/12 | *3/28* | 2/5 | 2/6 | *4/11* |
| **INx2** | 56 | SAM IgA | 0/2 | 2/7 | 2/4 | *4/13* | n/a | n/a | *n/a* |
| **INx2** | 56 | SAM IgG | 0/2 | 0/7 | 3/4 | *3/13* | n/a | n/a | *n/a* |
| **INx1 –IMx1** | 56 | SAM IgA | 1/3 | 1/2 | 3/6 | *5/11* | n/a | n/a | *n/a* |
| **INx1 –IMx1** | 56 | SAM IgG | 3/3 | 2/2 | 6/6 | *11/11* | n/a | n/a | *n/a* |

# References relating to supplementary material

1. Ramasamy MN, Minassian AM, Ewer KJ, Flaxman AL, Folegatti PM, Owens DR, et al. Safety and immunogenicity of ChAdOx1 nCoV-19 vaccine administered in a prime-boost regimen in young and old adults (COV002): a single-blind, randomised, controlled, phase 2/3 trial. Lancet. 2021;396(10267):1979-93.

2. Wilkins D, et al. Validation and performance of a multiplex serology assay to quantify antibody responses following SARS-CoV-2 infection or vaccination. Clinical & Translational Immunology 2022:e1385.

3. Folegatti PM, Ewer KJ, Aley PK, Angus B, Becker S, Belij-Rammerstorfer S, et al. Safety and immunogenicity of the ChAdOx1 nCoV-19 vaccine against SARS-CoV-2: a preliminary report of a phase 1/2, single-blind, randomised controlled trial. Lancet. 2020.

4. UK Health Security Agency. SARS-CoV-2 variant data update, England, Version 25 2022 [Available from: <https://assets.publishing.service.gov.uk/government/uploads/system/uploads/attachment_data/file/1063301/routine-variant-data-update-25-data-england-25-march-2022.pdf>.
