# Supplementary material for: Tolerability and immunogenicity of an intranasally-administered adenovirus-vectored COVID-19 vaccine: An open-label partially-randomised ascending dose phase I trial
Source: eBioMedicine. 2022 Oct 10;85:104298. doi: 10.1016/j.ebiom.2022.104298 (PMC9550199; doi:10.1016/j.ebiom.2022.104298)
Supplement: Supplementary file 2 [file mmc2.docx]

| **Trial Title:** | A Phase I study to determine safety, tolerability and immunogenicity of intranasal administration of the COVID vaccine ChAdOx1 nCOV-19 in healthy UK adults |
| --- | --- |
| **Short title:** | A Phase I study of intranasal ChAdOx1 nCOV-19 |
| **Study Reference:** | COV008 |
| **Protocol Version:** | 4.0 |
| **Date:** | 4 August 2021 |
| **Chief Investigator:** | Dr Alexander Douglas |
| **Funder:** | University of Oxford/ AstraZeneca |
| **EudraCT number:** | 2021-001047-27 |
| **REC Reference:** | 21/HRA/0699 |
| **IRAS Reference:** | 296376 |
| **Sponsor:** | University of Oxford |


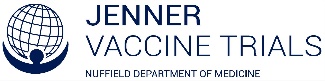


| **Key Trial Contacts** |  |
| --- | --- |
| **Chief Investigator** | Dr Alexander Douglas |
| **Address** | The Jenner Institute  Wellcome Centre for Human Genetics  Roosevelt Drive  Oxford, OX3 7BN  Email: [sandy.douglas@ndm.ox.ac.uk](mailto:sandy.douglas@ndm.ox.ac.uk) |
| **Clinical Trials Unit Investigators** | Prof Adrian Hill  The Jenner Institute, University of Oxford  Old Road Campus Research Building (ORCRB)  Roosevelt Drive Oxford OX3 7DQ  Email: [adrian.hill@ndm.ox.ac.uk](mailto:adrian.hill@ndm.ox.ac.uk)  Prof Sarah Gilbert  The Jenner Institute, University of Oxford  Old Road Campus Research Building (ORCRB)  Roosevelt Drive Oxford OX3 7DQ  Email: [sarah.gilbert@ndm.ox.ac.uk](mailto:sarah.gilbert@ndm.ox.ac.uk)    Prof Teresa Lambe  The Jenner Institute, University of Oxford  Old Road Campus Research Building (ORCRB)  Roosevelt Drive Oxford OX3 7DQ  Email: [teresa.lambe@ndm.ox.ac.uk](mailto:teresa.lambe@ndm.ox.ac.uk) |
| **Trial Sites** | **Centre for Clinical Vaccinology & Tropical Medicine**  University of Oxford  Churchill Hospital, Old Road, Headington, Oxford, OX3 7LE  PI at Site: Prof Adrian Hill  Email: adrian.hill@ndm.ox.ac.uk |
| **Sponsoring Institution** | **University of Oxford**  Clinical Trials and Research Governance  Joint Research Office,  Boundary Brook House  Churchill Drive, Headington  Oxford OX3 7GB  Email: [ctrg@admin.ox.ac.uk](mailto:ctrg@admin.ox.ac.uk) |
| **Funder** | AstraZeneca and University of Oxford |
| **Monitor** | Appledown Clinical Research Limited |
| **DSMB Chair** | Professor Charles Lacey,  Professor of Medicine,  Hull York Medical School,  University of York,  Heslington,  York, YO10 5DD |
|  | Signature:  <insert> |

**Confidentiality Statement**

This document contains confidential information that must not be disclosed to anyone other than the Sponsor, the Investigator Team, HRA, host organisation, and members of the Research Ethics Committee and other regulatory bodies. This information cannot be used for any purpose other than the evaluation or conduct of the clinical investigation without the prior written consent of Dr Alexander Douglas.

**Statement of Compliance**

The trial will be conducted in compliance with the protocol, the principles of Good Clinical Practice, Medicines for Human Use (Clinical Trial) Regulations 2004 (as amended) and all other applicable regulatory requirements.

**Investigator Agreement and Notification of Conflict of Interest**

I approve this protocol for use in the above-named clinical trial and agree to abide by all provisions set forth therein.

According to the Declaration of Helsinki, 2008, I have read this protocol, and declare the following conflict of interest. I am a named inventor on patents relevant to the manufacture of the ChAdOx1 nCoV-19 vaccine and as such may receive royalty income from licensing of the intellectual property by the University of Oxford to AstraZeneca. I have received research funding from AstraZeneca to support development of manufacturing methods for ChAdOx1 nCoV-19. I have a consultancy agreement with AstraZeneca but have not to date received any income through this.

| Chief Investigator  Dr Alexander Douglas | Signature  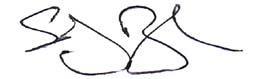 | Date:  <INSERT> |
| --- | --- | --- |

Site: **Centre for Clinical Vaccinology and Tropical Medicine, University of Oxford**

I have read this protocol and agree to abide by all provisions set forth therein.

According to the Declaration of Helsinki, 2008, I have read this protocol, and declare the following conflict of interest. AH is a cofounder of and minor shareholder in an Oxford University spin-off company, Vaccitech Ltd, that has some non-exclusive rights to the vector, ChAdOx1, used in the vaccine to be tested, that may be of commercial value”

| Principal Investigator  Prof. Adrian Hill | Signature  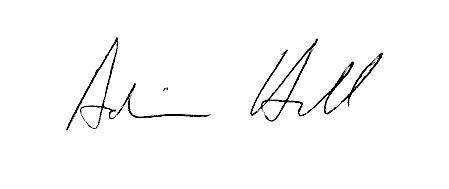 | Date:  <INSERT> |
| --- | --- | --- |

Table of Contents

[1 SYNOPSIS 10](#_Toc83220138)

[2 ABBREVIATIONS 13](#_Toc83220139)

[3 BACKGROUND AND RATIONALE 15](#_Toc83220140)

[3.1 Background 15](#_Toc83220141)

[3.2 Preclinical studies 16](#_Toc83220142)

[3.2.1 Immunogenicity by intramuscular route in mice (Jenner Institute, unpublished) 16](#_Toc83220143)

[3.2.2 Non-human primate efficacy and immunogenicity after intramuscular administration– NIH [7] 16](#_Toc83220144)

[3.2.3 Preclinical intranasal studies (pre-print) [9] 19](#_Toc83220145)

[3.2.3.1 Syrian hamsters 19](#_Toc83220146)

[3.2.3.2 Non-Human Primates (NHPs) 22](#_Toc83220147)

[3.3 Clinical experience 24](#_Toc83220148)

[3.3.1 ChAdOx1 nCOV-19 [10-12] 24](#_Toc83220149)

[3.3.1.1 Single dose and efficacy against asymptomatic infection 24](#_Toc83220150)

[3.3.2 Intranasal adenoviral vectored vaccines 24](#_Toc83220151)

[3.3.2.1 PanAd3 RSV [14, 15] 24](#_Toc83220152)

[3.3.2.2 NasoVAX 28](#_Toc83220153)

[3.3.2.3 Aerosolised ChAdOx1 85a (Jenner Institute, unpublished) 28](#_Toc83220154)

[3.3.2.4 Formulations and devices used in previous studies of intranasal adenovirus-vectored vaccines 28](#_Toc83220155)

[3.4 Antibody Dependant Enhancement and Immunopathology 28](#_Toc83220156)

[3.5 Rationale 29](#_Toc83220157)

[4 OBJECTIVES AND ENDPOINTS 31](#_Toc83220158)

[5 TRIAL DESIGN 32](#_Toc83220159)

[5.1 Study groups 32](#_Toc83220160)

[5.2 Trial volunteers 33](#_Toc83220161)

[5.3 Definition of End of Trial 33](#_Toc83220162)

[5.4 Duration of study 33](#_Toc83220163)

[5.5 Potential Risks for volunteers 33](#_Toc83220164)

[5.6 Known Potential Benefits 35](#_Toc83220165)

[6 RECRUITMENT AND WITHDRAWAL OF TRIAL VOLUNTEERS 36](#_Toc83220166)

[6.1 Identification of Trial Volunteers 36](#_Toc83220167)

[6.2 Informed consent 37](#_Toc83220168)

[6.3 Inclusion and exclusion criteria 38](#_Toc83220169)

[6.3.1 Inclusion Criteria 38](#_Toc83220170)

[6.3.2 Exclusion Criteria 38](#_Toc83220171)

[6.3.3 Effective contraception for female volunteers 40](#_Toc83220172)

[6.3.4 Prevention of ‘Over Volunteering’ 40](#_Toc83220173)

[6.3.5 Withdrawal of Volunteers 40](#_Toc83220174)

[6.4 Pregnancy 41](#_Toc83220175)

[6.5 Participants who are eligible to receive an approved or licensed SARS-CoV-2 vaccine 42](#_Toc83220176)

[7 TRIAL PROCEDURES 43](#_Toc83220177)

[7.1 Schedule of Attendance 43](#_Toc83220178)

[7.2 Observations 43](#_Toc83220179)

[7.3 Blood tests, Nasosorption, nasopharyngeal sampling, saliva and urinalysis 43](#_Toc83220180)

[7.4 Study visits 45](#_Toc83220181)

[7.4.1 Screening visit 45](#_Toc83220182)

[7.4.2 Day 0: Enrolment and vaccination visit 46](#_Toc83220183)

[7.4.2.1 Vaccination 46](#_Toc83220184)

[7.4.2.2 Post-vaccine fever and self-isolation 47](#_Toc83220185)

[7.4.2.3 Sequence of Enrolment and Vaccination of Volunteers 47](#_Toc83220186)

[7.4.3 Subsequent visits: 48](#_Toc83220187)

[7.4.4 Symptomatic volunteers 50](#_Toc83220188)

[7.4.5 Randomisation / allocation 50](#_Toc83220189)

[7.4.5.1 Randomisation to booster vaccination 50](#_Toc83220190)

[7.4.5.2 Allocation to groups 50](#_Toc83220191)

[7.5 Manufacturing and presentation 51](#_Toc83220192)

[7.5.1 Description of ChAdOx1 nCoV-19 51](#_Toc83220193)

[7.6 Supply 51](#_Toc83220194)

[7.7 Storage 51](#_Toc83220195)

[7.8 Administration 51](#_Toc83220196)

[7.9 Rationale for selected dose 52](#_Toc83220197)

[7.10 Minimising environmental contamination with genetically modified organisms (GMO) 52](#_Toc83220198)

[7.11 Compliance with Trial Treatment 53](#_Toc83220199)

[7.12 Accountability of the Trial Treatment 53](#_Toc83220200)

[7.13 Concomitant Medication 53](#_Toc83220201)

[8 ASSESSMENT OF SAFETY 54](#_Toc83220202)

[8.1 Definitions 54](#_Toc83220203)

[8.1.1 Adverse Event (AE) 54](#_Toc83220204)

[8.1.2 Adverse Reaction (AR) 54](#_Toc83220205)

[8.1.3 Serious Adverse Event (SAE) 54](#_Toc83220206)

[8.1.4 Serious Adverse Reaction (SAR) 55](#_Toc83220207)

[8.1.5 Suspected Unexpected Serious Adverse Reaction (SUSAR) 55](#_Toc83220208)

[8.2 Expectedness 55](#_Toc83220209)

[8.3 Foreseeable Adverse Reactions: 55](#_Toc83220210)

[8.4 Adverse Events of Special Interest (AESI) 55](#_Toc83220211)

[8.5 Causality 56](#_Toc83220212)

[8.6 Reporting Procedures for All Adverse Events 57](#_Toc83220213)

[8.7 Assessment of severity 58](#_Toc83220214)

[8.8 Reporting Procedures for Serious AEs 59](#_Toc83220215)

[8.8.1 Accident and Emergency (Emergency Department) Attendances 59](#_Toc83220216)

[8.8.2 Grade 4 Blood results 59](#_Toc83220217)

[8.8.3 Cases falling under Hy’s Law will be reported as SAEs. 60](#_Toc83220218)

[8.8.4 Grade 3 thrombocytopenia will be reported as an SAE 60](#_Toc83220219)

[8.9 Reporting Procedures for SUSARS 60](#_Toc83220220)

[8.10 Development Safety Update Report 60](#_Toc83220221)

[8.11 Procedures to be followed in the event of abnormal findings 60](#_Toc83220222)

[8.12 Interim Reviews 61](#_Toc83220223)

[8.13 Data Safety Monitoring Board 61](#_Toc83220224)

[8.14 Safety Group Holding Rules 62](#_Toc83220225)

[8.14.1 Group holding rules 62](#_Toc83220226)

[8.14.2 Individual stopping rules 63](#_Toc83220227)

[9 STATISTICS 65](#_Toc83220228)

[9.1 Description of Statistical Methods 65](#_Toc83220229)

[9.2 Safety & Reactogenicity 65](#_Toc83220230)

[9.3 Immunogenicity 65](#_Toc83220231)

[9.4 Procedure for Accounting for Missing, Unused, and Spurious Data. 65](#_Toc83220232)

[9.5 Inclusion in Analysis 65](#_Toc83220233)

[10 DATA MANAGEMENT 66](#_Toc83220234)

[10.1 Data Handling 66](#_Toc83220235)

[10.2 Record Keeping 66](#_Toc83220236)

[10.3 Source Data and Case Report Forms (CRFs) 67](#_Toc83220237)

[10.4 Data Protection 67](#_Toc83220238)

[10.5 Data Quality 67](#_Toc83220239)

[10.6 Archiving 68](#_Toc83220240)

[11 QUALITY CONTROL AND QUALITY ASSURANCE PROCEDURES 69](#_Toc83220241)

[11.1 Investigator procedures 69](#_Toc83220242)

[11.2 Monitoring 69](#_Toc83220243)

[11.3 Protocol deviation 69](#_Toc83220244)

[11.4 Audit & inspection 69](#_Toc83220245)

[12 SERIOUS BREACHES 70](#_Toc83220246)

[13 ETHICS AND REGULATORY CONSIDERATIONS 71](#_Toc83220247)

[13.1 Declaration of Helsinki 71](#_Toc83220248)

[13.2 Guidelines for Good Clinical Practice 71](#_Toc83220249)

[13.3 Ethical and Regulatory Approvals 71](#_Toc83220250)

[13.4 Volunteer Confidentiality 71](#_Toc83220251)

[14 FINANCING AND INSURANCE 72](#_Toc83220252)

[14.1 Financing 72](#_Toc83220253)

[14.2 Insurance 72](#_Toc83220254)

[14.3 Compensation 72](#_Toc83220255)

[15 Publication Policy 73](#_Toc83220256)

[16 Development of a new product/ process or the generation of intellectual property 74](#_Toc83220257)

[APPENDIX A: AMENDMENT HISTORY 77](#_Toc83220258)

[Appendix B. Toxicity grading scale for Lab AEs 79](#_Toc83220259)

# SYNOPSIS

| **Title** | A Phase I study to determine safety, tolerability and immunogenicity of intranasal administration of the COVID vaccine ChAdOx1 nCOV-19 in healthy UK adults | |
| --- | --- | --- |
| **Trial Identifier** | COV008 | |
| **Trial Registration** | EudraCT: 2021-001047-27  Clinicaltrials.gov: NCT04816019 | |
| **Chief Investigator** | Dr Alexander Douglas | |
| **Clinical Phase** | I | |
| **Design** | Open label, dose escalation study to investigate:   1. Safety, tolerability and immunogenicity of one or two doses of intranasal ChAdOx1 nCOV-19, in vaccine naïve individuals, with randomisation between one and two dose groups. 2. Safety, tolerability and immunogenicity of intranasal ChAdOx1 nCOV-19, given as a booster dose in individuals who have had two intramuscular COVID-19 vaccinations. | |
| **Population** | Group 1a: Healthy adults aged 18-40 years  Groups 1b/2/3 to date of implementation of SA003: Healthy adults aged 30-40 years  Groups 2b/3 from date of implementation of SA003: Healthy adults aged 18-55 years  Groups 4 and 5: Healthy adults aged 30-55 years | |
| **Planned Sample Size** | Up to 54   \| Group \| Vaccination status \| N= \| D0 \| D28 \| \| --- \| --- \| --- \| --- \| --- \| \| 1a \| COVID-19 vaccine naive \| 1 \| 5x10^9^vp ChAdOx1 nCOV-19 IN \| 1:1 randomisation to receive booster dose of 5x10^9^vp ChAdOx1 nCOV-19 IN \| \| 1b \| 5 \| 5x10^9^vp ChAdOx1 nCOV-19 IN \| \| 2a \| 3 \| 5x10^10^vp ChAdOx1 nCOV-19 IN \| 1:1 randomisation to receive booster dose of 5x10^10^vp ChAdOx1 nCOV-19 IN \| \| 2b \| Up to 15 \| 5x10^10^vp ChAdOx1 nCOV-19 IN \| \| 3 \| Up to 18 \| 2x10^10^vp ChAdOx1 nCOV-19 IN \| 1:1 randomisation to receive booster dose of 2x10^10^vp ChAdOx1 nCOV-19 IN \| \| 4 \| Two previous doses of ChAdOX1 nCoV-19 IM \| 6 \| 5x10^10^vp ChAdOx1 nCOV-19 IN \| - \| \| 5 \| Two previous doses of BNT162b2 IM \| 6 \| 5x10^10^vp ChAdOx1 nCOV-19 IN \| - \| | |
| **Visit Schedule:** | All volunteers:   - one screening visit - one vaccination visit (day 0) - follow-up visits: at days 7, 14, 28, 56 and 112   In addition:   - Groups 1a/2a: follow-up visit/call on day 3 - Groups 2b/3/4/5: additional visit on day 1, at the investigators’ discretion - Volunteers randomised to intranasal booster vaccination on day 28: follow up visits on days 35 and 42. - Section 7.1 covers possibility of additional visits on safety grounds.   All days are relative to first vaccination, day 0. | |
| **Planned Trial Duration** | 12 months in total  Approximately 4 months for each individual study participant (measured from the day of first vaccination) | |
|  | **Objective** | **Outcome Measure** |
| **Primary** | Investigation of safety and tolerability of intranasal administration of ChAdOx1 nCOV-19 in healthy adult volunteers | a) Occurrence of solicited local reactogenicity signs and symptoms for 7 days following vaccination |
|  |  | b) Occurrence of solicited systemic reactogenicity signs and symptoms for 7 days following vaccination |
|  |  | c) Occurrence of unsolicited adverse events (AEs) for 28 days following vaccination |
|  |  | d) Change from baseline for safety laboratory measures at D7 (and D35 for 2 dose groups only) |
|  |  | e) SAEs and AESIs collected throughout the trial |
| **Secondary** | To assess the mucosal immune response to intranasal administration of one or two doses of ChAdOx1 nCoV-19 in healthy adult volunteers, including characterisation of dose-response relationship. | Quantification of spike-binding mucosal antibody, collected using SAM strips at D0, D7, D14, D28, D42 (2 dose groups only), D56, D112 |
| **Exploratory** | Cellular and humoral response to vaccination, measured using a variety of exploratory immunological assays. | |
| **Investigational products** | ChAdOx1 nCoV-19, a replication-deficient simian adenoviral vector expressing the spike (S) protein of SARS-CoV-2 | |
| **Formulation** | ChAdOx1 nCoV-19: Liquid | |
| **Route of Administration** | Intranasal (IN) | |
| **Dose per Administration** | 5x10^9^vp, 2x10^10^vp or 5x10^10^vp ChAdOx1 nCOV-19 | |

# ABBREVIATIONS

| **ADE** | Antibody dependant enhancement |
| --- | --- |
| **AdHu** | Human adenovirus |
| **AdHu5** | Human adenovirus serotype 5 |
| **AE** | Adverse event |
| **AESI** | Adverse Events of Special Interest |
| **AID** | Autoimmune Disease |
| **BAL** | Bronchoalveolar lavage |
| **CCVTM** | Centre for Clinical Vaccinology and Tropical Medicine, Oxford |
| **CBF** | Clinical BioManufacturing Facility |
| **CEF** | Chick embryo fibroblast |
| **ChAd63** | Chimpanzee adenovirus 63 |
| **CI** | Confidence interval |
| **COP** | Code of Practice |
| **CRF** | Case Report Form or Clinical Research Facility |
| **CTRG** | Clinical Trials & Research Governance Office, Oxford University |
| **CTL** | Cytotoxic T Lymphocyte |
| **CVST** | Cerebral venous sinus thrombosis |
| **DSUR** | Development Safety Update Report |
| **ELISPOT** | Enzyme-linked immunospot |
| **GCP** | Good Clinical Practice |
| **GDPR** | General Data Protection Regulation |
| **GMO** | Genetically modified organism |
| **GMT** | Geometric Mean Titre |
| **GP** | General Practitioner |
| **HCG** | Human Chorionic Gonadotrophin |
| **HBV** | Hepatitis B virus |
| **HEK** | Human embryonic kidney |
| **HCV** | Hepatitis C virus |
| **HIT** | Heparin induced thrombocytopenia |
| **HIV** | Human immunodeficiency virus |
| **HLA** | Human leukocyte antigen |
| **HRA** | Health Research Authority |
| **HTLV** | Human T-Lymphotrophic Virus |
| **IB** | Investigator Brochure |
| **ICH** | International Conference on Harmonisation |
| **ICMJE** | International Committee of Medical Journal Editors |
| **ICS** | Intracellular Cytokine Staining |
| **ID** | Intradermal |
| **IFNγ** | Interferon gamma |
| **IM** | Intramuscular |
| **IMP** | Investigational Medicinal Product |
| **IMP-D** | Investigational Medicinal Product Dossier |
| **IN** | Intranasal |
| **IV** | Intravenous |
| **MAD** | Mucosal atomisation device |
| **MHRA** | Medicines and Healthcare Products Regulatory Agency |
| **MVA** | Modified vaccinia virus Ankara |
| **NAAT** | Nucleic acid amplification test |
| **NHS** | National Health Service |
| **NIH** | National Institutes of Health |
| **NIHR** | National Institute for Health Research |
| **NHP** | Non-human primate |
| **PBMC** | Peripheral blood mononuclear cell |
| **PCR** | Polymerase chain reaction |
| **PI** | Principal Investigator |
| **QP** | Qualified Person |
| **qPCR** | Quantitative polymerase chain reaction |
| **REC** | Research Ethics Committee |
| **SAM** | Synthetic absorptive matrix |
| **SAE** | Serious adverse event |
| **SC** | Subcutaneous |
| **SmPc** | Summary of Product characteristics |
| **SOP** | Standard Operating Procedure |
| **SUSAR** | Suspected unexpected serious adverse reaction |
| **µg** | microgram |
| **vp** | viral particle |
| **VV** | viral vector |
| **WHO** | World Health Organisation |

# BACKGROUND AND RATIONALE

## Background

In December 2019, a cluster of patients with pneumonia of unknown cause was linked to a seafood wholesale market in Wuhan, China and were later confirmed to be infected with a novel coronavirus, known as 2019-nCoV [1]. The virus was subsequently renamed to SARS-CoV-2 because it is similar to the coronavirus responsible for severe acute respiratory syndrome (SARS-CoV), a lineage B betacoronavirus. SARS-CoV-2 shares more than 79% of its sequence with SARS-CoV, and 50% with the coronavirus responsible for Middle East respiratory syndrome (MERS-CoV), a member of the lineage C betacoronavirus[2]. COVID-19 is the infectious disease caused by SARS-CoV-2. By January 2020 there was increasing evidence of human to human transmission as the number of cases rapidly began to increase in China. Despite unprecedented containment measures adopted by the Chinese government, SARS-CoV-2 rapidly spread across the world. The WHO declared the COVID-19 outbreak a public health emergency of international concern on 30^th^ January 2020. As of 9^th^ Feb 2021, over 106,125,682 cases have been reported to the WHO, with more than 2,320,497deaths worldwide [3].

Coronaviruses (CoVs) are spherical, enveloped, large positive-sense single-stranded RNA genomes. One-fourth of their genome is responsible for coding structural proteins, such as the spike (S) glycoprotein, envelope (E), membrane (M) and nucleocapsid (N) proteins. E, M, and N are mainly responsible for virion assembly whilst the S protein is involved in receptor binding, mediating virus entry into host cells during CoVs infection via different receptors.[4] SARS-CoV-2 belongs to the phylogenetic lineage B of the genus *Betacoronavirus* and it recognises the angiotensin-converting enzyme 2 (ACE2) as the entry receptor [5]. It is the seventh CoV known to cause human infections and the third known to cause severe disease after SARS-CoV and MERS-CoV.

The spike protein is a type I, trimeric, transmembrane glycoprotein located at the surface of the viral envelope of CoVs, which can be divided into two functional subunits: the N-terminal S1 and the C-terminal S2. S1 and S2 are responsible for cellular receptor binding via the receptor binding domain (RBD) and fusion of virus and cell membranes respectively, thereby mediating the entry of SARS-CoV-2 into target cells.[4] The roles of S in receptor binding and membrane fusion make it an ideal target for vaccine and antiviral development, as it is the main target for neutralising antibodies.

ChAdOx1 nCoV-19 vaccine consists of the replication-deficient simian adenovirus vector ChAdOx1, containing the structural surface glycoprotein (Spike protein) antigen of the SARS CoV-2 (nCoV-19), with a leading tissue plasminogen activator (tPA) signal sequence. ChAdOx1 nCoV-19 expresses a codon-optimised coding sequence for the Spike protein from genome sequence accession GenBank: MN908947. The tPA leader sequence has been shown to be beneficial in enhancing immunogenicity of another ChAdOx1 vectored CoV vaccine (ChAdOx1 MERS) [6].

## Preclinical studies

Refer to the Investigator Brochure for most recent pre-clinical data update.

### Immunogenicity by intramuscular route in mice (Jenner Institute, unpublished)

Mice (balb/c and CD-1) were immunised with ChAdOx1 expressing SARS-CoV-2 Spike protein or green fluorescent protein (GFP). Spleens were harvested for assessment of IFY ELISpot responses and serum samples were taken for assessments of S1 and S2 antibody responses on ELISA at 9 or 10 days post vaccination. The results of this study show that a single dose of ChAdOx1 nCoV was immunogenic in mice.

Two mouse strains (BALB/c, N=5 and outbred CD1, N=8) were vaccinated intramuscularly (IM) with ChAdOx1 nCoV-19 or ChAdOx1 GFP, a control vaccine expressing green fluorescent protein. Humoral and cellular immunity were studied 9-14 days later. Total IgG titers were detected against spike protein subunits S1 and S2 in all vaccinated mice. Profiling of the IgG subclasses showed a predominantly Th1 response post vaccination. Virus-specific neutralising antibodies were detected in all mice vaccinated with ChAdOx1 nCoV-19, whereas no neutralisation was detected in serum from mice vaccinated with ChAdOx1 GFP. Splenic T-cell responses measured by IFN-γ ELISpot and intracellular cytokine staining (ICS) were detected against peptides spanning the full length of the spike construct. Again, a strong Th1-type response was detected post vaccination as supported by high levels of IFN-γ and TNF-α, and low levels of IL-4 and IL-10.

### Non-human primate efficacy and immunogenicity after intramuscular administration– NIH [7]

In this study, two groups of rhesus macaques were utilized. Animals were adults, vaccinated group contained six animals, control group contained three animals. Group 1 was vaccinated with ChAdOx1 nCoV-19 at a dose of 2.5 x 10^10^ vp/animal at 28 days before challenge. Group 2 (control) was vaccinated with ChAdOx1 GFP at a dose of 2.5 x 10^10^ vp/animal at 28 days before challenge. The dose is half that which is planned for humans.

Animals were challenged with 2.6 x 10^6^ TCID¬50/animal of SARS-CoV-2 using 4 routes: intranasal (0.5ml per nostril), intratracheal (4ml), oral (1ml), and ocular (0.25ml per eye) of a 4x10^5^ TCID50/ml virus dilution in sterile DMEM.

Animals were examined on 1, 3, 5, and 7 days post challenge and were euthanized at 7 days post challenge.

1. **Humoral response**

Antibodies in serum against SARS-CoV-2 spike protein were measured by ELISA. An increase in ELISA titre and neutralizing antibodies was found when comparing serum obtained before initial vaccination and at day of challenge.

1. **Cytokine response**

Cytokines in serum were analysed after challenge to monitor immune responses. An upregulation in IFN-γ at 1 DPI in ChAdOx1 nCoV-19 was observed in vaccinated animals, but not in control animals. No significant differences were observed between ChAdOx1 nCoV-19 and control animals for TNF-α, IL-2, IL-4, IL-6, and IL-10.

1. **Shedding of SARS-CoV-2 virus**

Viral gRNA load was high in lung tissue of control animals and viral sgRNA was detected in 2 out of 3 control animals (Figure ***1***). In contrast, the viral gRNA load was significantly lower in lung tissue obtained from vaccinated animals as determined via Mann-Whitney’s rank test and below limits of detection in two vaccinated animals. Viral sgRNA was detected in lung tissue obtained from 1 out of 6 vaccinated animals (p<0.0001). Viral gRNA could be detected in other tissues but was low in both groups (Figure ***2***).

1. **Pulmonary pathology**

At 7 days post inoculation, all animals were euthanized, and tissues were collected. None of the vaccinated monkeys developed pulmonary pathology after inoculation with SARS-CoV-2. All lungs were histologically normal and no evidence of viral pneumonia nor immune-enhanced inflammatory disease was observed. In addition, no SARS-CoV-2 antigen was detected by immunohistochemistry in the lungs of any of the vaccinated animals. Two out of 3 control animals developed some degree of viral interstitial pneumonia.


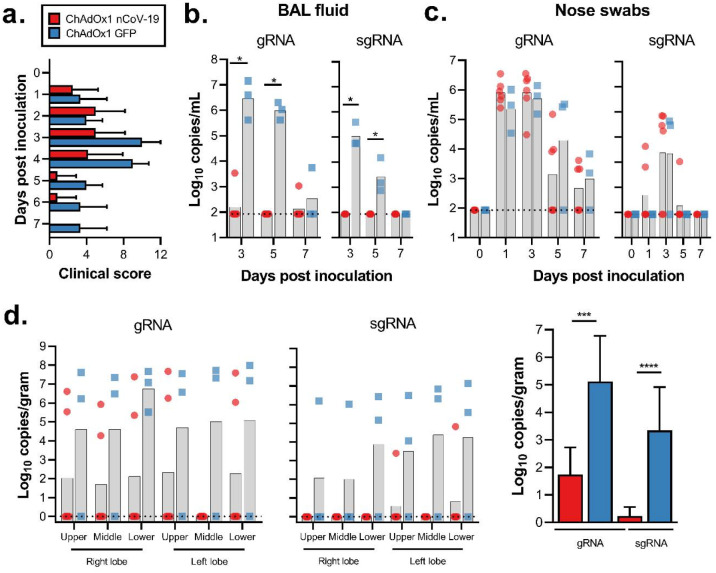


Figure *1*. *(Reproduced from [8]) Clinical signs and viral load in rhesus macaques inoculated with SARS-CoV-2 after vaccination with ChAdOx1 nCoV-19. a. Mean clinical score with standard deviation in NHPs. Any scoring associated with food was removed from final score. b. Viral load in BAL fluid obtained from rhesus macaques, bar at geometric mean. *=p-value<0.0166. c. Viral load in nose swabs obtained from rhesus macaques, bar at geometric mean. d. Viral load in tissues at 7 DPI. Pictured are individual values with geometric mean bars (left panels) and geometric mean of all lung lobes per group (right panel). ***=p-value<0.001; ****=p-value<0.0001. Vaccinated animals = red circles; control animals = blue squares; dotted line = limit of detection.*


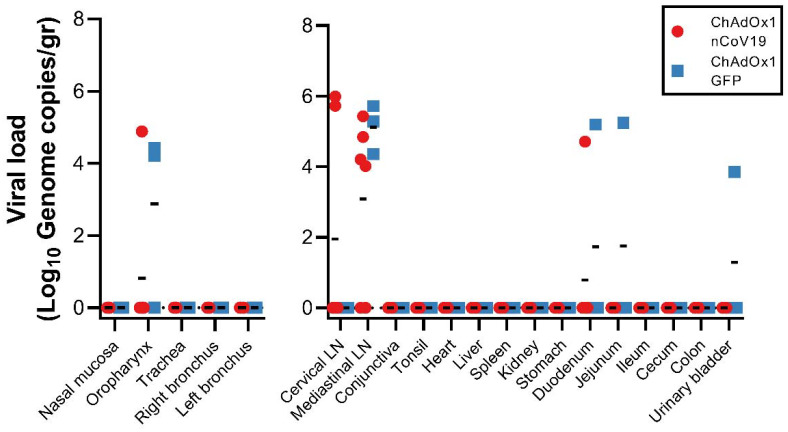


Figure *2*. *(Reproduced from [8]) Viral load in rhesus macaques challenged with SARS-CoV-2. Viral genomic RNA in respiratory tissues excluding lung tissue (left panel) and other tissues (right panel). A two-tailed Mann-Whitney’s rank test was performed to investigate statistical significance. Bonferroni correction was applied, and thus statistical significance was reached at p>0.0125.*

### Preclinical intranasal studies (pre-print) [9]

In further pre-clinical studies, Van Doremalen et al investigated the effect of intranasal ChAdOx1 nCOV-19 administration on lung disease and nasal shedding of virus in rodents (hamsters) and primates (rhesus macaques).

#### Syrian hamsters

3 groups of 10 Syrian hamsters were vaccinated with the following regimes: 1. Intranasal (IN) ChAdOx1 nCOV-19, 2. Intramuscular (IM) ChAdOx1 nCOV-19, 3. ChAdOx1 GFP (control). Animals were inoculated intranasally 28 days post vaccination using isolate SARS-CoV-2/human/USA/RML-7/2020 (containing D614G mutation in the S protein). 6 animals in each group were swabbed daily for 7 days post infection. This experiment was then repeated using horizontal transmission from infected animals rather than direct IN challenge.

1. **Immunogenicity**

Antibody responses were measured on the day of infection. High IgG titres were seen in both vaccination groups, with no significant difference between IN and IM. Neutralising antibody titres, however, were higher in the group that received IN vaccination.

1. **Mucosal shedding**

Analysis of the area under the curve (AUC) showed that total amount of viral RNA and infectious virus shedding was significantly lower in the IN group, compared to the control group (Figure 3.). This difference was not seen between the IM and control groups. Similar results were seen in the horizontal transmission experiment.

1. **Pulmonary pathology**

4 animals in each group were euthanised 5 days post infection. In the control group, all animals were found to have pulmonary lesions (40-70% of the tissue). There was no pathology observed in the lungs of animals from either vaccination group. SARS-CoV-2 N antigen was only found in the lungs of control group animals.

In hamsters infected by horizontal transmission, there was also no pathology observed in the lungs in the IN group. All animals in the control group were found to have lesions in the lung tissue (40-50% of tissue). 3 out of 4 animals in the IM group displayed pulmonary lesions (5-20% of tissue). SARS-CoV-2 N antigen was found in control animals, and in the IM group, although to a lesser extent.


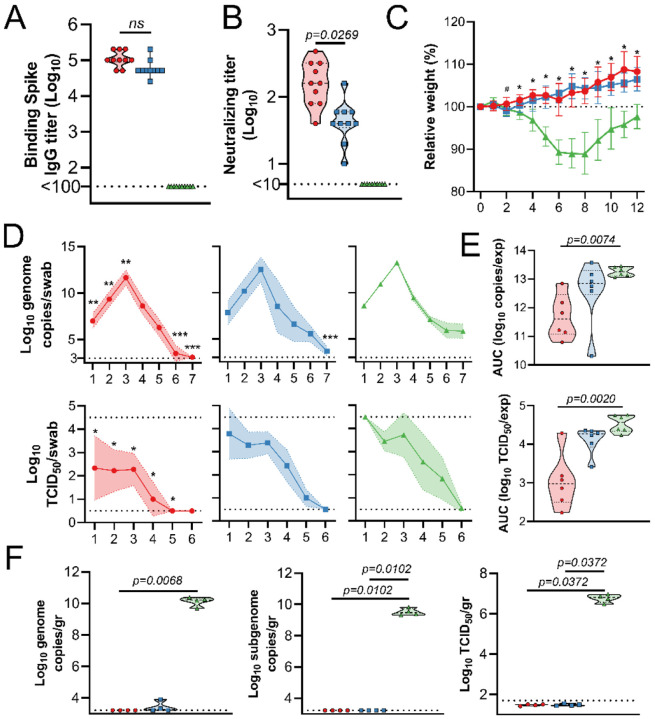


*Figure 3. (Reproduced from [9]) Hamsters were vaccinated via the IN route (red), IM route (blue) or with control vaccine ChAdOx1 GFP via the IM route (green). A. Binding antibody titers against SARS-CoV-2 S protein. B. Virus neutralizing antibody titers. C. Relative weight upon challenge with SARS-CoV-2. A-B. Shown is geometric mean and 95% confidence interval. # = p-value <0.05 between IN and control group; * = p-value <0.05 between vaccinated groups and control group. D. Viral load and viral titer in oropharyngeal swabs. Shown is geometric mean (symbols) and 95% confidence interval (shade). E. Area under the curve analysis of viral load and titer shedding in oropharyngeal swabs. F. Viral load and titer in lung tissue, isolated at 5 DPI. E-F. Dashed line = median; dotted line = quartiles. Statistical analyses done using mixed-effect analyses (C), two-way ANOVA (D), or Kruskal-Wallis test (E-F). * = p value <0.05; ** = p-value < 0.01; *** = p-value < 0.001.*

#### Non-Human Primates (NHPs)

Two groups of 4 rhesus macaques were vaccinated as follows: 1. Intranasal prime-boost ChAdOx1 nCOV-19 (using a mucosal atomisation device) 2. ChAdOx1 GFP (control). Challenge was 28 days post boost vaccination, via both intranasal and intratracheal routes, using 10^6^ TCID_50_ of SARS-CoV-2 (SARS-CoV-2/human/USA/RML-7/2020). Animals were euthanised 7 days after challenge.

1. **Immunogenicity**

At 7 days post prime vaccination, SARS-CoV-2 specific IgG were detected in nasosorption and serum samples, but not in bronchoalveolar lavage (BAL). Following boost, higher titres of IgG were obtained. Specific IgA was detected in BAL following booster vaccination, but not following prime. Neutralising antibodies were detected in all animals. Levels were similar to those detected in convalescent human sera and NHPs vaccinated with IM prime-boost ChAdOx1 nCOV-19.

1. **Mucosal shedding and pulmonary viral load**

Following challenge, nasal swabs were taken to investigate for mucosal virus shedding. Vaccinated animals had fewer positive viral swabs than the unvaccinated controls, with only a single swab from a single animal (‘NHP1’) positive for each of subgenomic RNA and culturable virus. In addition they had a lower viral load. No infectious virus was detected in BAL samples from vaccinated animals, compared to 2/4 animals in the control group (Figure 4). Although small numbers (n=4 vaccinees) precluded statistically robust analysis for immunological correlates of protection, relationships between immunological and virological outcome measures were consistent with the hypothesis that high spike-specific nasal (and serum) antibody levels may be associated with reduced nasal shedding, while high BAL antibody levels may be associated with pulmonary protection.


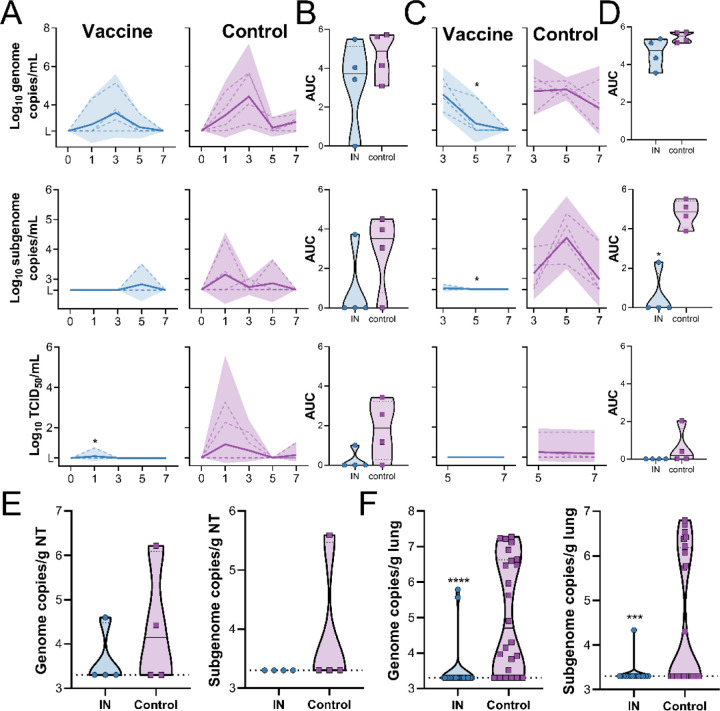


*Figure 4. (Reproduced from [9]). SARS-CoV-2 detection in samples obtained from rhesus macaques upon virus challenge. gRNA, sgRNA and infectious virus in nasal swabs (A) and BAL (C) was determined. Dotted line = individual animals; solid line = geometric mean; shaded area = 95% confidence interval. Area under the curve (AUC) was calculated as an indication of the total amount of virus shed in nasal swabs (B) and BAL (D) and displayed as a truncated violin plot. Solid line = median; dotted line = quartiles. * = p-value <0.05 as determined via two-tailed Mann-Whitney test. Amount of gRNA and sgRNA in nasal turbinate (E) and lung tissue (F). Blue = vaccinated animals; purple = control animals; solid line = median; dotted line = quartiles. *** = p-value <0.001; **** = p-value <0.0001, as determined via two-tailed Mann-Whitney test.*

## Clinical experience

### ChAdOx1 nCOV-19 [10-12]

Further information can be found in the summary of product characteristics.

ChAdOx1 nCOV-19 has been approved for emergency use in the UK by the MHRA, using a regimen of two intramuscular doses, given 4 or 12 weeks apart. It is currently being widely delivered in the UK and there is now extensive clinical experience of its use. Common side effects include pain, feeling feverish, chills, muscle ache, headache, and malaise. These effects appear to be less pronounced in recipients in older age groups.

#### Single dose and efficacy against asymptomatic infection

Analysis of the most recent data from clinical studies (pre-print [13]) shows 76% (59%, 86%) efficacy for a single dose of vaccine (22-90 days post vaccination). Antibody levels are maintained during this period. With a standard dosing regimen, no evidence is seen of efficacy against asymptomatic infection (2.0%, 95%CI (-50.7%, 36.2%, 41 ChAdOx1 nCoV-19 versus 42 control cases)). However, in the low dose-standard dose group efficacy was higher (49.3%, 95%CI (7.4%, 72.2%, 16 ChAdOx1 nCoV-19 versus 31 control cases)), which may relate to a trend to increasing efficacy against asymptomatic infection with increasing prime-boost interval [13].

### Intranasal adenoviral vectored vaccines

#### PanAd3 RSV [14, 15]

Oxford Vaccine Group have completed a phase I study in healthy adults (NCT01805921), comparing intranasal and intramuscular administration of PanAd3- RSV. 21 volunteers received priming with IN PanAd3- RSV (5 x 10^9^ vp: n=4, 5 × 10^10^ vp: n=16). Solicited AEs collected for in the IN group were: nasal pain, discharge and irritation. Volunteers in the IN reported few side effects in the week post vaccination. 5 out of 21 reported short, mild, and self-limiting sore throat reactions within 1 week of vaccination. Adverse events noted from blood sampling and observations were generally mild. A single volunteer in the IN group developed a fever 3 days following vaccination. This was concurrent with an influenza-like illness and rhinovirus was detected on nasal sample polymerase chain reaction (Figure 5).

Nasal samples collected 3 days after IN PanAd3-RSV did not detect any shedding of vaccine virus. No mucosal immunology results are available.

Fewer volunteers made systemic antibody responses to IN PanAd3-RSV prime and these were of a lower magnitude. Volunteers primed with IN PanAd3-RSV, however, made comparable post-boost responses to IM PanAd3-RSV–primed volunteers, irrespective of whether boost was with PanAd3 or MVA.

At baseline, PBMCs from 19 of 33 (58%) volunteers had detectable IFN-g T cell responses to at least one peptide pool. Following prime this had increased to 16 of 18 (89%) and 16 of 19 (85%) after IM and IN PanAd3-RSV, respectively. The magnitude of the response was greater following IM prime than after IN prime (Figure 6). Prime with IM PanAd3-RSV induced a significant rise in circulating PanAd3 neutralizing antibody. Prime with IN PanAd3-RSV did not (Figure 7)***.***


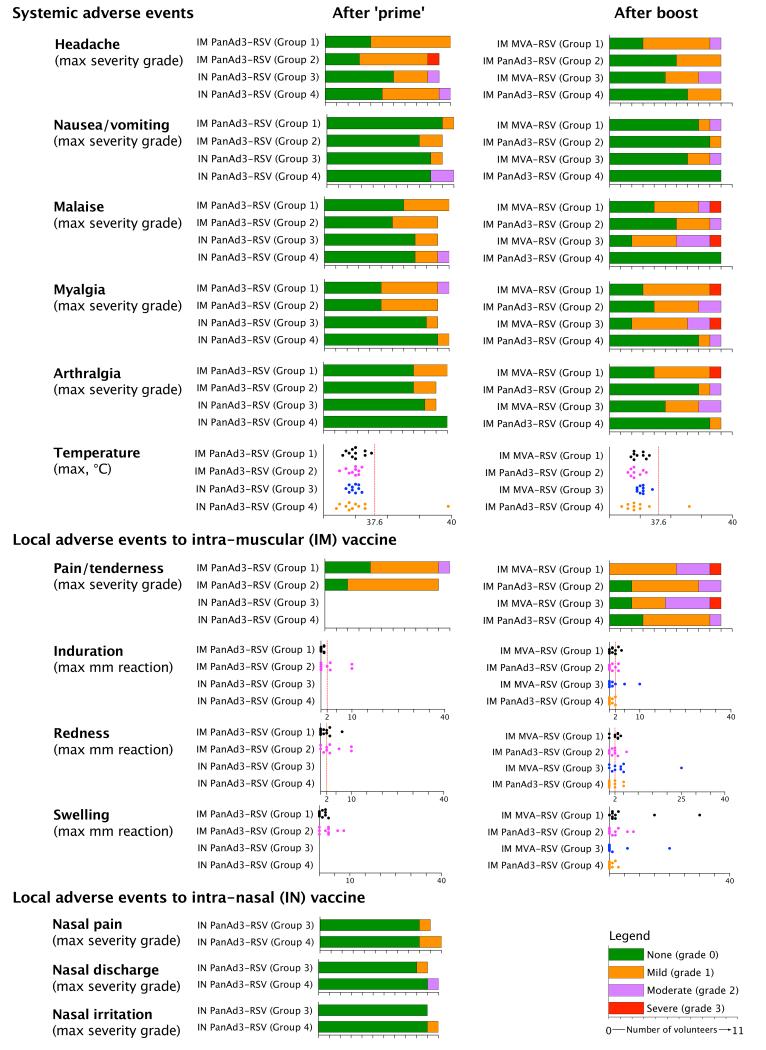

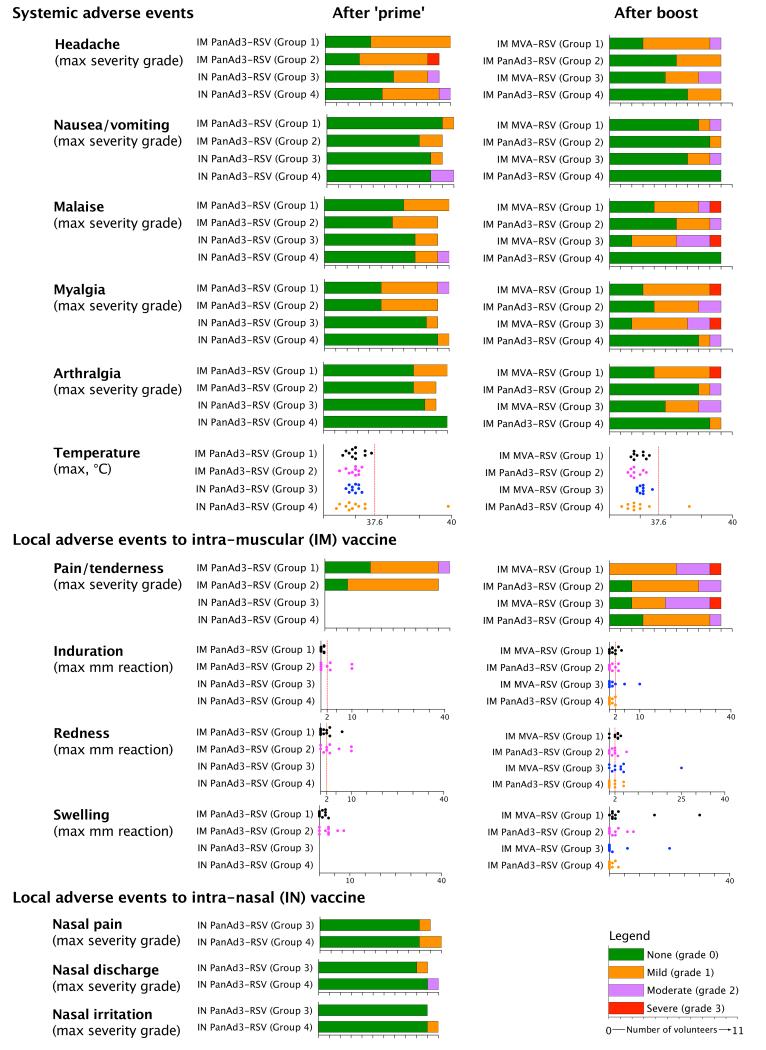

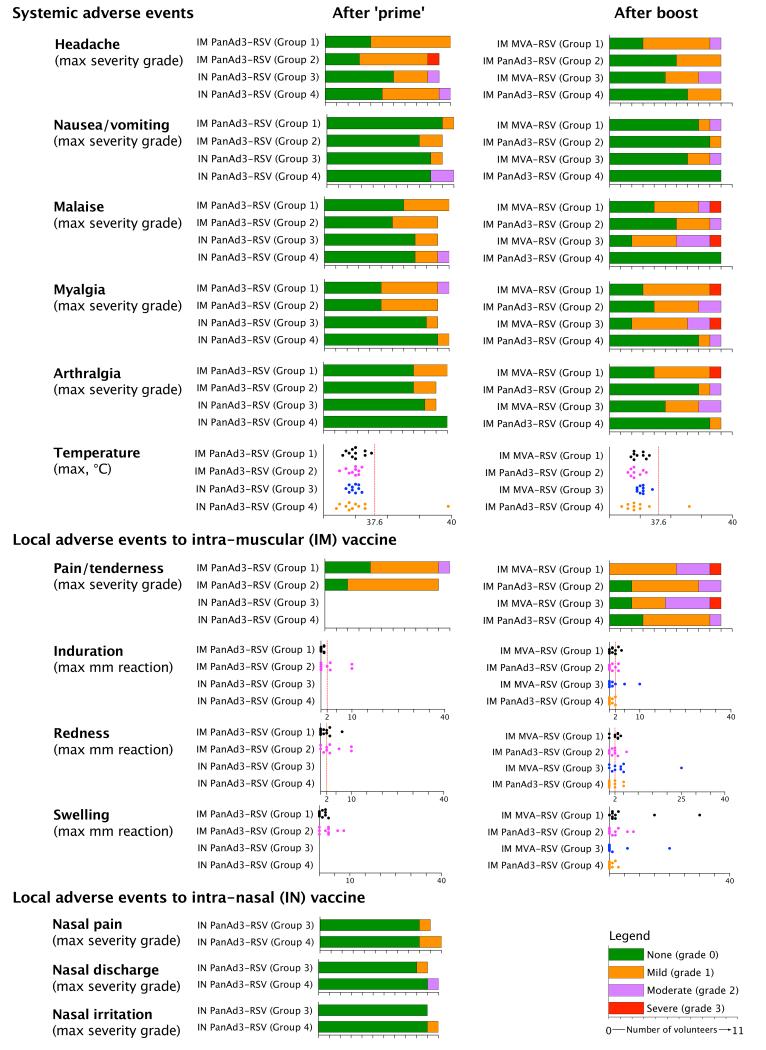


*Figure 5. (Reproduced from [15] )Frequency of the maximum severity solicited adverse event, oral temperature and size of local injection site reactions within one week of vaccination. The number of volunteers is represented across the x-axis without distinction between low-dose and target-dose recipients; n=10 or 11 for events after prime and n=10 for events after boost due to withdrawals. Groups 1 & 2 received intramuscular prime (followed by different IM boost vaccinations, for which data is not shown); groups 3 & 4 received intranasal prime (and different IM boost vaccinations, for which data not shown). Volunteers reported subjective symptoms as none, mild (does not interfere with routine activities), moderate (interferes with routine activities) and severe (unable to perform routine activities). Redness, swelling and induration at the site of injection used the maximal recorded diameter of any reaction for objective severity grading. Fever was graded as none (≤37.6°C), mild (37.6.0-38.0°C), moderate (38.1-39.0°C) and severe (≥39.1°C). Overall 5587/5593 (99.9%) of expected data points for solicited adverse events within one week after vaccination were collected for analysis. The only missing data was for temperature recordings. Sore throat reactions were not a solicited symptom although occurred as an unsolicited event in 5/21 IN primed volunteers.*


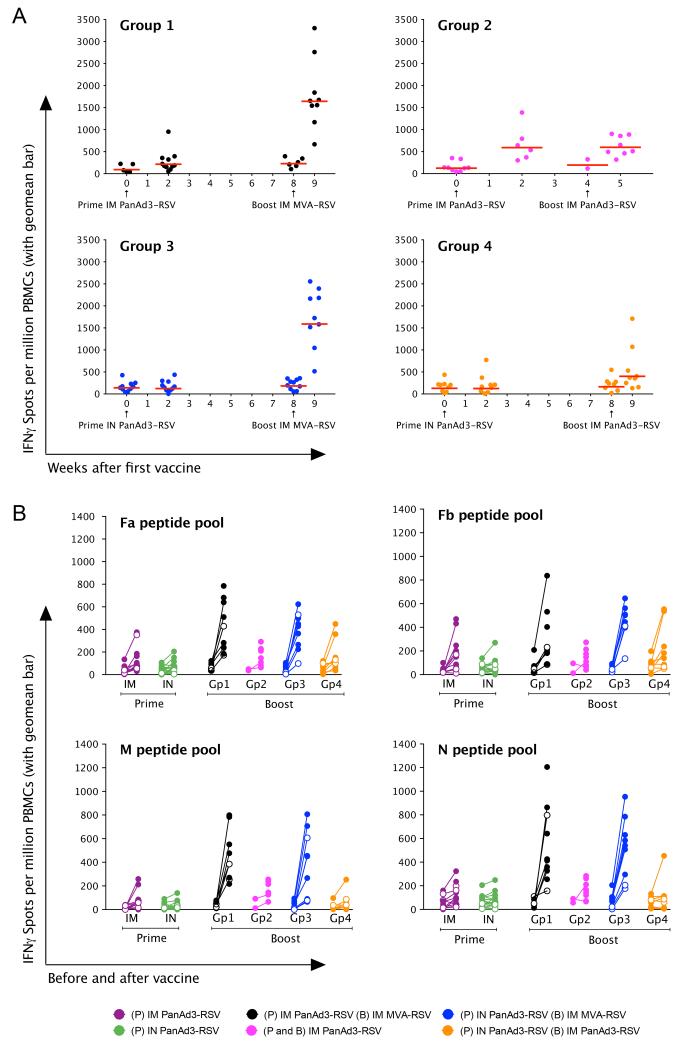


*Figure 6. (Reproduced from* ***[15]****) The >ex-vivo T-cell IFNγ response to vaccination. Groups 1 &2, and groups 3 & 4, differed from each other only in boost vaccinations, all of which were intramuscular.* *Fresh PBMCs were collected for ex-vivo IFNγ ELISpot analysis at baseline, two weeks after prime, before boost and one week after boost. Cells were stimulated overnight by peptide pools Fa, Fb, M and N being representative of the vaccine antigens. The results for each group presented by scatter plot of the summed response for each volunteer [(Fa+Fb+M+N) − (4xDMSO)]. The red line denotes the geometric mean (18%) of samples. There was a disproportionate loss of group 2 pre-boost samples. A further 5 peptide responses from 3 volunteers were rejected with a triplicate variance greater than 10.*


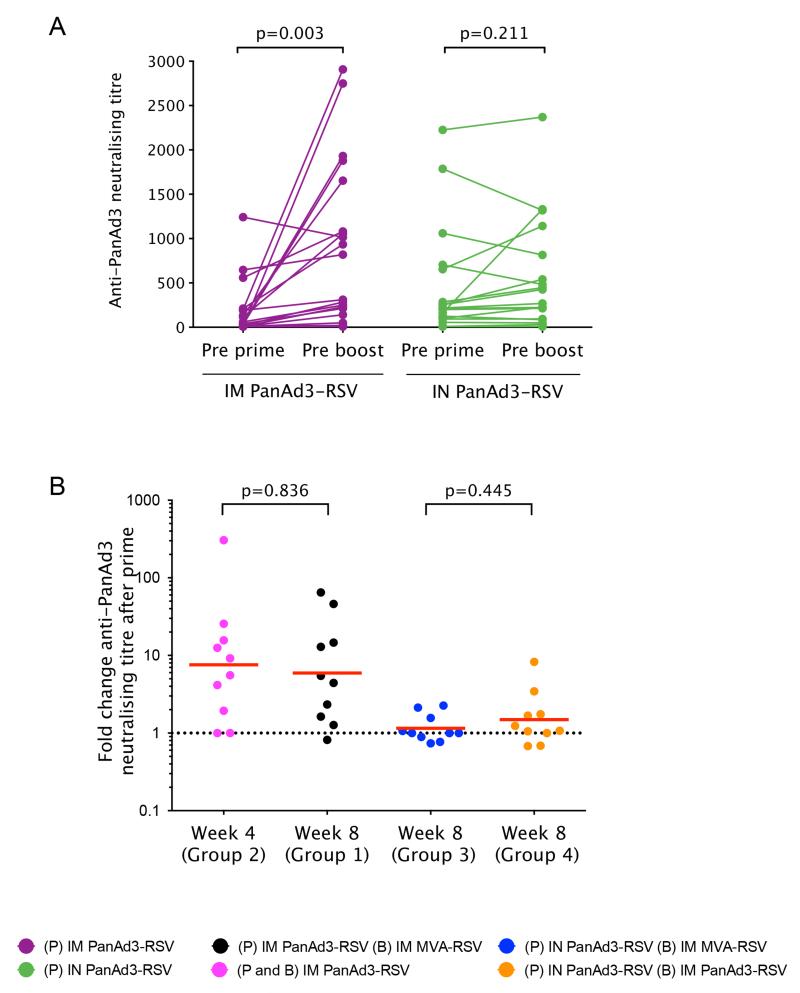


*Figure 7. (Reproduced from [15] ) Vector neutralising antibody (anti-PanAd3) titres before prime and before boost vaccination. Anti-PanAd3 titres were measured for the 40 volunteers who completed the trial. No pre-screening of anti-PanAd3 titres was performed before enrolment and study group allocation. (A) Scatter plot of the anti-PanAd3 titre from before prime (baseline) and before boost vaccine. The lower limit of detection for the assay was a titre of 18, and titres ≤18 were arbitrarily assigned a titre of 9. (B) Fold change in anti-PanAd3 neutralising antibody after IM and IN PanAd3-RSV prime. The red bar denotes the geometric mean*

#### NasoVAX

NasoVAX is a human adenovirus-vectored vaccine (AdHu5). Intranasal delivery has been tested in a phase 2a study in healthy adults (NCT03232567). 4 groups of 15 volunteers were respectively vaccinated with the following doses: 1×10^9^vp, 1×10^10^ vp, 1×10^11^ vp or saline placebo. It was reported that the vaccine was safe and well tolerated, with no SAEs recorded [16-18].

#### Aerosolised ChAdOx1 85a (Jenner Institute, unpublished)

This was a phase I trial in healthy adults (NCT04121494). 3 groups of 3 volunteers had the following doses of ChAdOx1 85A administered via the aerosolised route: 1 x 10^9^ vp, 5x10^9^vp, 1 x 10^10^vp. Following this, a further 20 volunteers received aerosol ChAdOx1 85A at the highest dose (1 x 10^10^vp).

The lower doses of vaccine were well tolerated. At the highest dose, mild-moderate respiratory (cough, dyspnoea, rhinitis, sore throat, chest pain) symptoms were reported by a minority of volunteers. Systemic AEs were mostly mild to moderate. One volunteer receiving the highest dose of vaccine reported grade 3 fever, chills, fatigue and haematoid sputum. This was assessed as likely secondary to bronchoscopy, rather than vaccination. No SAEs were reported.

#### Formulations and devices used in previous studies of intranasal adenovirus-vectored vaccines

All of the above studies have used formulations from the set originally developed by Evans & Volkin[19], with sucrose, sodium chloride, disodium edetate, magnesium chloride, ethanol, histidine and polysorbate 80 as excipients. These formulations were originally developed primarily to ensure adenovirus stability in storage, rather than specifically for the purpose of intranasal delivery, but tolerability has been good as described above. The ChAdOx1 85A study used an identical formulation to that used for ChAdOx1 nCoV-19 (‘A438’). The PanAd3 RSV and Nasovax studies used formulations optimised for stability of those particular adenovirus serotypes, differing from only in the relative proportions of sucrose and sodium chloride, and pH buffer (7.4 with Tris and histidine buffers, vs 6.6 with histidine only for A438). Both intranasal studies (PanAd3 and Nasovax) used the same ‘MAD’ spray device for administration as will be used in this study. This produces droplets 30-100 μm in size (https://www.lmaco.com/products/lma-mad-nasal), similar to the Accuspray device used for Flumist (live attenuated intranasal influenza vaccine).

## Antibody Dependant Enhancement and Immunopathology

Safety concerns around the use of full-length coronavirus Spike glycoproteins and other viral antigens (nucleoprotein) as a vaccine antigen have been raised following historical and limited reports of immunopathology and antibody dependant enhancement (ADE) reported *in vitro* and post SARS-CoV challenge in mice, ferrets and non-human primates immunised with whole SARS-CoV inactivated or full-length S protein-based vaccines, including a study using Modified Vaccinia Ankara as a vector.[20-22] To date, there has been one report of lung immunopathology following MERS-CoV challenge in mice immunised with an inactivated MERS-CoV candidate vaccine.[23] However, in preclinical studies of ChAdOx1 immunisation and MERS-CoV challenge, no ADE was observed in hDPP4 transgenic mice, dromedary camels or non-human primates [24, 25].

The NHP study described above showed no evidence of immune-enhanced inflammation at 7 days post challenge in ChAdOx1 nCoV-19 vaccinated animals who underwent SARS-CoV-2 challenge 4 weeks post immunisation. A separate challenge study conducted after immunisation with a purified inactivated SARS-CoV-2 vaccine also reported no evidence of ADE in vaccinated animals [26]. No evidence of vaccine-related disease enhancement has been seen in any of the clinical trials of this vaccine.

## Rationale

Data from both observational and modelling studies suggest that asymptomatic shedding and transmission of COVID-19 infection is a significant issue in control of the pandemic [27, 28]. It appears that intramuscular vaccination with ChAdOx1 nCoV-19 has limited efficacy against asymptomatic infection. Preclinical data suggest that intranasal vaccination may reduce viral shedding from the nasal mucosa, as well as preventing infection.

ChAdOx1 nCOV-19 is a strong candidate for intranasal vaccination. There is already extensive safety data and proven efficacy against symptomatic infection after intramuscular administration. In addition, there is data from intranasal administration in NHPs (non-GLP). The current formulation can be administered intranasally using a mucosal atomisation device (MAD) attached to a standard syringe after withdrawal of the product from a conventional vial. The mucosal atomisation device is CE marked and will be used within its intended purpose.

A meta-analysis, comparing intramuscular (inactivated) and intranasal (live-attenuated) influenza vaccination showed that although IN vaccination stimulated lower systemic IgG than IM, it induced higher mucosal IgA and equivalent protection [29]. Clinical studies of IN PanAd3 RSV studies showed that IN vaccination generates very low serum antibody responses, but that this does not impair immunogenicity following boost. It is thus doubtful that serum measures of immunogenicity will be predictive of efficacy after IN vaccination. For this reason, we propose a trial with only limited serum immunology studies and prioritisation of the collection of safety data and samples for mucosal immunology studies to enable a subsequent Phase II proof-of-concept efficacy study.

We also propose that participants in groups 1-3 will be randomly assigned to receive either one or two IN doses. This will provide safety and tolerability data on both dosing approaches, while allowing for a comparison of mucosal immune responses and information on whether the second dose boosts such responses. Randomisation will ensure that bias is minimised in the read out of immunological comparisons between those receiving a single dose and two doses.

The initial study age range proposed in March 2021 was 18-40. The rationale for restriction to under 40s was to facilitate recruitment of COVID-19 vaccine-naïve participants who could be followed up for at least 56 days without becoming eligible for NHS vaccination. This was amended to 30-40 by SA001 after concerns about vaccine-induced thrombosis emerged from experience of intramuscular vaccination. By early June 2021, all over 30s had become eligible for vaccination by the NHS. At this point the study age range was amended again, to 18-55, by SA003, in order to facilitate recruitment. 18-29 year olds, some of whom had not yet become eligible for NHS vaccination, were added after discussion with the MHRA vaccines Expert Working Group regarding the acceptability of thrombosis risk after intranasal vaccination in this study. 41-55 year olds were added as, by June, there was no longer a rationale to restrict to under 40s (as all over 30s had become eligible for NHS vaccination, those aged 30-55 are similar in that any remaining vaccine-naïve and hence potentially eligible individuals must not have taken up this opportunity). Our expectation is that the majority of volunteers enrolled following the implementation of SA003 will be from the under 30 age bracket.

As of 10^th^ August 2021, 12 participants have been recruited to group 2 and 11 participants to group 3. An increasing proportion of the UK population have received COVID-19 vaccines, and so there are few remaining vaccine-naïve volunteers eligible for enrolment to these groups, and complete recruitment of the originally targeted numbers (24 in each group) no longer appears possible. Recruitment will therefore be terminated at the investigators’ discretion. There is however increasing recognition of the possibility that booster vaccinations may be required in due course, in particular to improve control of milder COVID-19 infection and asymptomatic transmission. As an additional aim, the study will therefore seek to investigate the use of intranasal ChAdOx1 nCoV-19 as a booster vaccination following intramuscular vaccination. Volunteers who have had either ChAdOx1 nCoV-19 or BNT162b2 (BioNtech, Pfizer), the two most widely available COVID-19 vaccines in the UK, will be included, as groups 4 and 5 respectively. These groups are small (n=6 each) and intended to provide limited pilot data to assist a decision on whether a larger future study would be worthwhile. There are large numbers of individuals potentially eligible for these groups and so we do not feel it is necessary to include the under 30 age group: the expansion of the study to groups 4 and 5 by SA005 is therefore limited to recruitment of participants aged between 30-55 years.

# OBJECTIVES AND ENDPOINTS

Study objectives and endpoints are set out in Table 1.

| Outcome | Objective | Outcome measure |
| --- | --- | --- |
| Primary | Investigation of safety and tolerability of intranasal administration of ChAdOx1 nCOV-19 in healthy adult volunteers | a) Occurrence of solicited local reactogenicity signs and symptoms for 7 days following vaccination  b) Occurrence of solicited systemic reactogenicity signs and symptoms for 7 days following vaccination  c) Occurrence of unsolicited adverse events (AEs) for 28 days following vaccination  d) Change from baseline for safety laboratory measures at D7 (and D35 for 2 dose groups only)  e) SAEs and AESIs collected throughout the trial |
| Secondary * | To assess the mucosal immune response to intranasal administration of one or two doses of ChAdOx1 nCoV-19 in healthy adult volunteers, including characterisation of dose-response relationship. | Quantification of spike-binding mucosal antibody, collected using SAM strips at D0, D7, D14, D28, D42 (2 dose groups only), D56, D112 |
| Exploratory* | Cellular and humoral response to vaccination, measured using a variety of exploratory immunological assays. | |

**Table 1** *Objectives and Endpoints. *Sample analysis for the completion of secondary and exploratory endpoints may be performed under the ethically approved OVC Biobank protocol.*

Overall, the study aims to enable subsequent evaluation of intranasal ChAdOx1 nCoV-19 for safety and efficacy in a larger cohort of participants.

# TRIAL DESIGN

## Study groups

The study will be divided into five groups. Groups 1-3 will consist of vaccine-naïve volunteers and groups 4-5 will consist of volunteers who have received 2 doses of IM COVID-19 vaccine.

Groups 1-3 will receive three vaccine dose levels (low i.e. 5x10^9^vp, high i.e. 5x10^10^ VP, and moderate i.e. 2x10^10^ VP ChAdOx1 nCOV-19 IN). The low and high dose groups will each have safety lead-in subgroups. Groups are outlined in the table below. All volunteers will be consented for receipt of a booster vaccine at approximately 4 weeks post first vaccine. All volunteers will be randomly allocated 1:1 to receive an intranasal booster vaccine as indicated, stratified by first dose level.

Group 4 will consist of volunteers who have received 2 intramuscular doses of ChAdOx1 nCoV-19, at least eight weeks apart, and group 5 will consist of volunteers who have received 2 intramuscular doses of BNT162b, at least three weeks apart. Both of these groups will receive a single standard dose (5x10^10^vp) on intranasal ChAdOx1 nCOV-19.

| Group | Vaccination status | N= | D0 | D28 |
| --- | --- | --- | --- | --- |
| 1a | COVID-19 vaccine naive | 1 | 5x10^9^vp ChAdOx1 nCOV-19 IN | 1:1 randomisation to receive booster dose of 5x10^9^vp ChAdOx1 nCOV-19 IN |
| 1b |  | 5 | 5x10^9^vp ChAdOx1 nCOV-19 IN |  |
| 2a |  | 3 | 5x10^10^vp ChAdOx1 nCOV-19 IN | 1:1 randomisation to receive booster dose of 5x10^10^vp ChAdOx1 nCOV-19 IN |
| 2b |  | Up to 15 | 5x10^10^vp ChAdOx1 nCOV-19 IN |  |
| 3 |  | Up to 18 | 2x10^10^vp ChAdOx1 nCOV-19 IN | 1:1 randomisation to receive booster dose of 2x10^10^vp ChAdOx1 nCOV-19 IN |
| 4 | Two previous doses of ChAdOX1 nCoV-19 IM | 6 | 5x10^10^vp ChAdOx1 nCOV-19 IN | - |
| 5 | Two previous doses of BNT162b2 IM | 6 | 5x10^10^vp ChAdOx1 nCOV-19 IN | - |

Table 2 Trial Groups

## Trial volunteers

Healthy adult volunteers aged 18-55 years will be recruited into the study. Recruitment for groups 4 and 5 will be limited to volunteers aged 30-55 years. Volunteers will be considered enrolled immediately following administration of first vaccination.

N.B. The volunteer enrolled in group 1a, prior to implementation of SA001, was enrolled while volunteers age 18-40 were eligible. Volunteers enrolled in groups 1b and 2a, and groups 2b and 3 up to the date of implementation of SA003, were enrolled while volunteers age 30-40 were eligible.

## Definition of End of Trial

The end of the trial is the date of the last participant’s last visit.

## Duration of study

The total duration of the study will be approximately 4 months for each individual participant, measured from the day of the first vaccination dose. The whole study, from initiation to completion is expected to last approximately 12 months.

## Potential Risks for volunteers

The potential risks are those associated with phlebotomy and vaccination (including disease enhancement and thrombosis/thrombocytopenia).

**Venepuncture**

Localised bruising and discomfort can occur at the site of venepuncture. Infrequently fainting may occur. These will not be documented as AEs if they occur. The maximum total volume of blood drawn over the study period of about 4 months will be approximately 399 ml (blood volumes may vary slightly for volunteers at different investigator sites due to use of different volume vacutainers, following local Trust SOPs). This should not compromise these otherwise healthy volunteers, as they would donate 470mL during a single blood donation for the National Blood and Transplant service over a 3-4 month period. Volunteers will be asked to refrain from blood donation for the duration of their involvement in the trial.

**Allergic reactions**

Allergic reactions from mild to severe may occur in response to any constituent of a medicinal product’s preparation. Anaphylaxis is extremely rare (about 1 in 1,000,000 vaccine doses) but can occur in response to any vaccine or medication.

**Vaccination**

*Local reactions from intranasal vaccination*

Volunteers may experience sneezing, nasal discharge, nasal tenderness or pain, nasal irritation or sore throat post intranasal vaccination.

*Systemic reactions*

Constitutional influenza-like symptoms such as fatigue, headache, malaise, feverishness, and muscle aches can occur with any vaccination and last for 2-3 days. Presyncopal and syncopal episodes may occur at the time of vaccination which rapidly resolve. As with any other vaccine, temporary ascending paralysis (Guillain-Barré syndrome, GBS) or immune mediated reactions that can lead to organ damage may occur, but this should be extremely rare (1 in 100,000-1,000,000 vaccine doses).

A syndrome of thrombosis (including cerebral venous sinus thrombosis [CVST] or in some cases thromboses in the splanchnic veins or other large veins) and thrombocytopenia has been reported in some recipients of this vaccine after intramuscular administration during population-wide rollout. Incidence and risk factors for this remain under investigation; reports suggest an incidence of the order of 1:100,000 recipients. The syndrome has similarities to heparin-induced thrombocytopenia (HIT). Intravenous immunoglobulin and non-heparin anticoagulation are recommended as treatments although their efficacy is as yet uncertain.

The mechanism by which vaccination could induce this syndrome is not known, but induction of high titer antibodies against Platelet Factor 4 has been observed in some individuals with CVST after vaccination. Some proposed mechanisms require interaction of the adenovirus vector with the Platelet Factor 4 (present in serum), or platelets themselves. Such interaction may be less likely after intranasal than parenteral administration. To the Chief Investigator’s knowledge, there is no evidence of physical presence of adenovirus in the affected veins; instead induction of platelet factor 4 auto-antibodies is a systemic phenomenon with diverse local effects, as seen in HIT. The investigators are not aware of grounds to believe that the risk of this syndrome would be higher after intranasal vaccination; on the contrary it is hypothesized that the risk may be lower, if the mechanism involves contact of the vector with serum protein or platelets.

The clinical trial context allows a range of risk mitigation measures to be implemented, including exclusion of participants with relevant risk factors, platelet count monitoring and close clinical monitoring to allow early diagnosis and treatment in the unlikely event a study volunteer were to develop evidence of this condition.

**Disease Enhancement**

Induction of disease enhancement and lung immunopathology in the event of COVID-19 disease following ChAdOx1 nCoV-19 vaccination has not been observed in clinical studies of this vaccine to date, as described in section 3.4. It is therefore considered not to be a significant risk.

## Known Potential Benefits

Recipients of intranasal ChAdOx1 nCoV-19 will not receive any guaranteed benefit. However, it is hoped that the information gained from this study will contribute to the development of a safe and effective vaccine against COVID-19. The only benefit for participants would be in information about their general health status.

# RECRUITMENT AND WITHDRAWAL OF TRIAL VOLUNTEERS

## Identification of Trial Volunteers

Healthy adults in the UK will be recruited by use of an advertisement +/- registration form formally approved by the ethics committee(s) and distributed or posted in the following places:

- In public places, including buses and trains, with the agreement of the owner / proprietor.
- In newspapers or other literature for circulation.
- On radio via announcements.
- On a website or social media site operated by our group or with the agreement of the owner or operator (including on-line recruitment through our web-site).
- By e-mail distribution to a group or list only with the express agreement of the network administrator or with equivalent authorisation.
- By email distribution to individuals who have already expressed an interest in taking part in any clinical trial at the Oxford Vaccine Centre and other trial sites.
- On stalls or stands at exhibitions or fairs.
- Via presentations (e.g. presentations at lectures or invited seminars).
- Direct mail-out: This will involve obtaining names and addresses of adults via the most recent Electoral Roll. The contact details of individuals who have indicated that they do not wish to receive postal mail-shots would be removed prior to the investigators being given this information. The company providing this service is registered under the UK General Data Protection Regulation (UK GDPR) 2016/679. Investigators would not be given dates of birth or ages of individuals but the list supplied would only contain names of those aged between 18-55 years (as per the inclusion criteria).
- Direct mail-out using National Health Service databases: These include the National Health Applications and Infrastructure Services (NHAIS) via a NHAIS data extract or equivalent. Initial contact to potential participants will not be made by the study team. Instead, study invitation material will be sent out on our behalf by an external company, CFH Docmail Ltd, in order to preserve the confidentiality of potential participants. CFH Docmail Ltd is accredited as having exceeded standards under the NHS Digital Data Security and Protection Toolkit (ODS ID – 8HN70).
- Oxford Vaccine Centre databases and other trial sites databases: We may contact individuals from databases of groups within the CCVTM (including the Oxford Vaccine Centre database) and other trial sites of previous trial participants who have expressed an interest in receiving information about all future studies for which they may be eligible.

## Informed consent

All volunteers will sign and date the informed consent form before any study specific procedures are performed. The information sheet will be made available to the volunteer at least 24 hours prior to the screening visit. At the screening visit, a video presentation of the aims of the study and all tests to be carried out may be screened to an audience, or made available for them to access remotely. Individually each volunteer will have the opportunity to question an appropriately trained and delegated researcher before signing the consent. At the screening visit, the volunteer will be fully informed of all aspects of the trial, the potential risks and their obligations. The following general principles will be emphasised:

- Participation in the study is entirely voluntary.
- Refusal to participate involves no penalty or loss of medical benefits.
- The volunteer may withdraw from the study at any time.
- The volunteer is free to ask questions at any time to allow him or her to understand the purpose of the study and the procedures involved.
- The study involves research of an investigational vaccine.
- There is no direct benefit to the volunteer from participating.
- The volunteer’s GP will be contacted to corroborate their medical history. Written or verbal information regarding the volunteer’s medical history will be sought from the GP or other sources. This can either be via the study team accessing patient’s electronic care summaries, GP and other medical records from local systems, by contacting the GP practice, or volunteers bringing their medical care summaries from the GP to the study clinicians. However, volunteers may be enrolled based on medical information obtained during screening only, at the physician’s discretion.
- Samples taken as part of the study may be sent outside of the UK and Europe to laboratories in collaboration with the University of Oxford. These will be anonymised. Volunteers will be asked if they consent to indefinite storage of any leftover samples for use in other ethically approved research, this will be optional.
- The volunteer will be registered on the TOPS database (The Over volunteering Prevention System; [www.tops.org.uk](http://www.tops.org.uk) ).

The aims of the study and all tests to be carried out will be explained. The volunteer will be given the opportunity to ask about details of the trial, and will then have time to consider whether or not to participate. Due to pressure on the study team during the current crisis, appropriately trained nursing staff (under the supervision of a medically qualified investigator) may be delegated to discuss the trial with volunteers and to report the content of that discussion to the investigator prior to signature of the consent form. In addition, all participants will have the opportunity to discuss the study with a medically qualified investigator if they wish. If they do decide to participate, they, and the investigator will sign and date the consent form. The volunteer will then be provided with a copy of the consent form to take away and keep, with the original being stored in the case report form (CRF). Reconsent, if required, will be taken by appropriately trained and delegated members of the team.

## Inclusion and exclusion criteria

This study will be conducted in healthy adults, who meet the following inclusion and exclusion criteria:

### Inclusion Criteria

| The volunteer must satisfy all the following criteria to be eligible for the study: | |
| --- | --- |
|  | Groups 1, 2 and 3 only: Healthy adults aged 18-55 years * |
|  | Groups 4 and 5 only: Healthy adults aged 30-55 years |
|  | Able and willing (in the Investigator’s opinion) to comply with all study requirements. |
|  | Willing to allow the investigators to discuss the volunteer’s medical history with their General Practitioner and access all medical records when relevant to study procedures. |
|  | For females only, willingness to practice continuous effective contraception (see below) during the study and a negative pregnancy test on the day(s) of screening and vaccination. |
|  | Agreement to refrain from blood donation during the course of the study. |
|  | Provide written informed consent. |
|  | Group 4 only: Prior receipt of 2 doses of ChAdOx1 nCoV-19 intramuscularly, with an interval of at least 8 weeks between the intramuscular doses, and with the second dose a minimum of 12 weeks prior to enrolment |
|  | Group 5 only: Prior receipt of 2 doses of BNT162b2 intramuscularly, with an interval of at least 3 weeks between the intramuscular doses, and with the second dose a minimum of 12 weeks prior to enrolment |

*N.B. Healthy adults aged 18-40 years were eligible for inclusion, prior to implementation of SA001. A single participant under the age of 30 was enrolled in group 1a before the implementation of SA001. This participant will be retained for follow-up but will not receive a booster vaccine, in light of the new age restrictions. Between the implementation of SA001 and implementation of SA003, only those aged 30-40 years were eligible.

Table 3. *Inclusion criteria*

### Exclusion Criteria

| The volunteer may not enter the study if any of the following apply: | |
| --- | --- |
|  | Receipt or planned receipt of any vaccine other than the study intervention within 28 days before and after each study vaccination. |
|  | Participation in COVID-19 prophylactic drug trials for the duration of the study.  (Note: Participation in COVID-19 treatment trials is allowed in the event of hospitalisation due to COVID-19. The study team should be informed as soon as possible.) |
|  | Groups 1, 2 and 3 only: Prior receipt of an investigational or licensed vaccine likely to impact on interpretation of the trial data (e.g. Adenovirus vectored vaccines, any coronavirus vaccines*). |
|  | Administration of immunoglobulins and/or any blood products within the three months preceding the planned administration of the vaccine candidate. |
|  | Any confirmed or suspected immunosuppressive or immunodeficient state, including HIV infection; asplenia; recurrent severe infections and use of immunosuppressant medication within the past 6 months, except topical steroids or short-term oral steroids (course lasting <14 days). |
|  | Any autoimmune conditions, except mild psoriasis, well-controlled autoimmune thyroid disease, vitiligo or stable coeliac disease not requiring immunosuppressive or immunomodulatory therapy. |
|  | History of allergic disease or reactions likely to be exacerbated by any component of ChAdOx1 nCoV-19. |
|  | Any history of angioedema. |
|  | Any history of anaphylaxis. |
|  | Pregnancy, lactation or willingness/intention to become pregnant during the study. |
|  | History of cancer (except basal cell carcinoma of the skin and cervical carcinoma in situ). |
|  | History of any organic central nervous system disorder or any functional disorder involving neurological symptoms |
|  | History of serious psychiatric condition likely to affect participation in the study (e.g. ongoing severe depression, history of admission to an in-patient psychiatric facility, recent suicidal ideation, history of suicide attempt, bipolar disorder, personality disorder, alcohol and drug dependency, severe eating disorder, psychosis, use of mood stabilisers or antipsychotic medication). |
|  | Bleeding disorder (e.g. factor deficiency, coagulopathy or platelet disorder), or prior history of significant bleeding or bruising following IM injections or venepuncture, or continuous anticoagulation e.g. with warfarin |
|  | History of confirmed major thrombotic event, (including cerebral venous sinus thrombosis, deep vein thrombosis, pulmonary embolism) or,  History of antiphospholipid syndrome. |
|  | Prior receipt of unfractionated heparin |
|  | History of heparin induced thrombocytopenia |
|  | Any other serious chronic illness requiring hospital specialist supervision. |
|  | Chronic respiratory diseases, including mild asthma (resolved childhood asthma is allowed). |
|  | Chronic cardiovascular disease (including hypertension), gastrointestinal disease, liver disease (except Gilberts Syndrome), renal disease, endocrine disorder (including diabetes) and neurological illness (excluding migraine). |
|  | Nasal pathology (e.g. congenital abnormalities such as an abnormal septum or polyps, previous cauterisation, rhinoplasty or nasal surgery of any kind, recurrent epistaxis). |
|  | Seriously overweight (BMI≥40 Kg/m^2^) or underweight (BMI≤18 Kg/m^2^). |
|  | Suspected or known current alcohol abuse as defined by an alcohol intake of greater than 42 units every week. |
|  | Suspected or known injecting drug abuse in the 5 years preceding enrolment. |
|  | Any clinically significant abnormal finding on screening biochemistry, haematology blood tests or urinalysis. |
|  | Any other significant disease, disorder or finding which may significantly increase the risk to the volunteer because of participation in the study, affect the ability of the volunteer to participate in the study or impair interpretation of the study data. |
|  | Groups 1, 2, and 3 only: Living in the same household as any vulnerable groups at risk of severe COVID-19 disease (as per PHE guidance).* |
|  | Groups 1, 2, and 3 only: Membership of any group identified by JCVI at the time of enrolment as being eligible for priority vaccination (i.e. ahead of the typical member of the individual’s age cohort). * |

Table 4. *Exclusion Criteria *NB volunteers in group 4 and 5 will have previously had COVID-19 vaccine, as detailed in inclusion criteria and therefore membership of high risk groups or household exposures are not relevant.*

### Effective contraception for female volunteers

Female volunteers of childbearing potential are required to use an effective form of contraception whilst enrolled in the trial.

| Acceptable forms of contraception for female volunteers include: | |
| --- | --- |
|  | Established use of oral, injected or implanted hormonal methods of contraception. |
|  | Placement of an intrauterine device (IUD) or intrauterine system (IUS). |
|  | Total abdominal hysterectomy. |
|  | Bilateral tubal Occlusion |
|  | Barrier methods of contraception (condom or occlusive cap with spermicide). |
|  | Male sterilisation, if the vasectomised partner is the sole partner for the subject. |
|  | True abstinence, when this is in line with the preferred and usual lifestyle of the subject. Periodic abstinence (e.g., calendar, ovulation, symptothermal, post-ovulation methods), declaration of abstinence for the duration of exposure to IMP, and withdrawal are not acceptable methods of contraception |

Table 5. *Acceptable forms of female contraception*

### Prevention of ‘Over Volunteering’

Volunteers will be excluded from the study if they are concurrently involved in another trial where an IMP has been administered within 28 days prior to enrolment, or will be administered during the trial period. In order to ensure this, volunteers will be asked to provide their National Insurance or Passport number (if they are not entitled to a NI number) and will be registered on a national database of participants in clinical trials ([*www.tops.org.uk*](http://www.tops.org.uk)). They will not be enrolled if found to be actively registered on another trial until further information on IMP and bleeding schedule is obtained.

### Withdrawal of Volunteers

In accordance with the principles of the current revision of the Declaration of Helsinki and any other applicable regulations, a volunteer has the right to withdraw from the study at any time and for any reason, and is not obliged to give his or her reasons for doing so. The Investigator may withdraw the volunteer at any time in the interests of the volunteer’s health and well-being. In addition, the volunteer may withdraw/be withdrawn for any of the following reasons:

- Administrative decision by the Investigator.
- Ineligibility (either arising during the study or retrospectively, having been overlooked at screening)*.
- Significant protocol deviation.
- Volunteer non-compliance with study requirements.
- An AE, which requires discontinuation of the study involvement or results in inability to continue to comply with study procedures.

The reason for withdrawal will be recorded in the CRF. If withdrawal is due to an AE, appropriate follow-up visits or medical care will be arranged, with the agreement of the volunteer, until the AE has resolved, stabilised or a non-trial related causality has been assigned. The DSMB or DSMB chair may recommend withdrawal of volunteers.

Any volunteer who is withdrawn from the study may be replaced, if that is possible within the specified time frame.

If a volunteer withdraws from the study, data and blood samples collected before their withdrawal will still be used on the analysis. Storage of blood samples will continue unless the participant specifically requests otherwise.

In all cases of subject withdrawal, long-term safety data collection, including some procedures such as safety bloods, will continue as appropriate if subjects have received one or more vaccine doses, unless they decline any further follow-up.

*Volunteers enrolled before implementation of SA001 may be 18-40 years old. Due to the change in eligibility criteria regarding age range (see section 6.3.1), any volunteers under the age of 30 years enrolled before implementation of SA001 did not meet eligibility criteria between implementation of SA001 and implementation of SA003. These volunteers will not be withdrawn from the study. They will not receive further vaccinations but will continue with follow-up visits and procedures detailed in Table 8.

## Pregnancy

Should a volunteer become pregnant during the trial, no further study IMP will be administered. She will be followed up for clinical safety assessment with her ongoing consent and in addition will be followed until pregnancy outcome is determined, following the procedures outline in SOP OVC005. We would not routinely perform venepuncture in a pregnant volunteer unless there is clinical need. Given that no routine bloods will be drawn from pregnant volunteers, follow-up visits may be conducted over the phone/video consultation in order to minimise contact and exposure from SARS-CoV-2 in pregnant volunteers.

## Participants who are eligible to receive an approved or licensed SARS-CoV-2 vaccine

Trial participants who are offered COVID-19 vaccination via the NHS can receive vaccination outside the study, if they choose. As described in section 3.3.2.1, the trial of IN PanAd3-RSV did not suggest concerns regarding induction of anti-vector immunity or reduced immunogenicity from an IM boost following IN priming.

Participants will be requested to inform the study team if they wish to take up an offer of vaccination outside the study, and will be advised not to take another COVID-19 vaccine within 28 days of receiving either a first or second dose of the nasal study vaccine.

We will take a neutral stance if volunteers wish to defer receiving an approved vaccine by up to 56 days after enrolment in the study. We will actively advise against deferral of an approved vaccine, if offered, beyond study day 56 (this 56-day period may extend beyond the 28 days we recommend waiting after a study vaccine for those volunteers receiving a single study vaccine).

Vaccination with an approved or licensed COVID-19 vaccine according to government policy will not be considered a withdrawal from the study. Participants who are offered and accept a licensed COVID-19 vaccine will be asked to continue in the study.

# TRIAL PROCEDURES

This section describes the trial procedures for evaluating study participants and follow-up after administration of study vaccine.

## Schedule of Attendance

Scheduled study visits are listed in Table 8. All volunteers will attend a screening visit and at least one vaccination visit. The number of follow-up visits is dependent on group and whether the volunteer is randomised to receive a booster vaccination. The total number of visits will be between 7 and 10, depending on group.

Additional visits for safety purposes may be added, up to a total visit frequency of 2 per week, for up to 4 weeks after either prime or boost vaccination, if emerging data on post-vaccination thrombosis suggests, in the opinion of medically-qualified investigators and the DSMB, that this would contribute to mitigation of risk of thrombosis.

## Observations

Pulse, blood pressure and temperature will be measured at the time-points indicated in the schedule of procedures and may also be measured as part of a physical examination if indicated at other time-points.

## Blood tests, Nasosorption, nasopharyngeal sampling, saliva and urinalysis

Blood will be drawn for the following laboratory tests and processed at agreed NHS Trust laboratories using NHS standard procedures:

- **Haematology;** Full Blood Count.
- **Biochemistry;** Sodium, Potassium, Urea, Creatinine, Albumin, Liver Function Tests (ALT, ALP, Bilirubin).
- **Diagnostic serology;** HBsAg, HCV antibodies, HIV antibodies (specific consent will be gained prior to testing blood for these blood-borne viruses).
- **Immunology;** Human Leukocyte Antigen (HLA) typing.

Additional safety blood tests may be performed if clinically relevant at the discretion of the medically qualified investigators.

At University of Oxford research laboratories:

**Immunology:** Immunogenicity will be assessed by a variety of immunological assays, in accordance with a trial immunology plan. This may include antibodies to SARS-CoV-Spike and non-Spike antigens by ELISA, ex vivo ELISpot assays for interferon gamma and flow cytometry assays, neutralising and other functional antibody assays and B cell analyses. Other exploratory immunological assays including cytokine analysis and other antibody assays, DNA analysis of genetic polymorphisms potentially relevant to vaccine immunogenicity and gene expression studies amongst others may be performed at the discretion of the Investigators.

**Urinalysis**

- Urine will be tested in the clinic for protein, blood and glucose at screening. For female volunteers only, urine will be tested for beta-human chorionic gonadotrophin (β-HCG) at screening and immediately prior to vaccination.

**Nasosorption**

- Synthetic absorptive matrix (SAM) strips are small pieces of absorbent material which are designed for sampling mucosal fluid and will be used in this study to sample fluid from the nasal mucosa (https://mucosaldiagnostics.com/). Sampling will take place using CE marked SAM strips for their intended purpose of nasal sampling, in line with manufacturer instructions. Briefly the absorbent tip is inserted into the participant’s nostril and the participant presses against the side of that nostril for 60 seconds. The device is then removed from the nostril and returned to the tube and stored for subsequent analysis. This may be carried out in clinic by an appropriately trained and delegated member of clinical staff. Volunteers may also be asked to undertake self-sampling using the SAM strips, either in the clinic or at home. Appropriate instructions will be provided for this. Samples will be used to investigate mucosal immunogenicity, particularly IgA. Given the small quantity of nasal fluid collected, volunteers may in some instances be asked to supply two samples collected the same day, typically one from each nostril.

**Saliva**

- Saliva will be collected and used for exploratory immunology assays.

**Nasopharyngeal sampling**

- At the investigators’ discretion, nasopharyngeal samples may be collected from selected groups as indicated in the visit schedule.
- Nasopharyngeal swabs will be collected using CE-marked devices in accordance with the manufacturer’s instructions.
- For a subset of up to 10 volunteers in group 2b, CE-marked cytology brushes may be used to obtain a larger number of cells for exploratory immunological assays at screening (for these volunteers, at least seven days before vaccination), and on day 1 after vaccination. Brushings would be performed as previously described [30], and in discussion with Dr Tim Hinks, consultant respiratory physician, who has previously used this technique locally in healthy volunteer research. Collection of the pre-vaccination sample at least seven days prior to vaccination will allow recovery of any microtrauma to the nasal epithelium before vaccination.

Collaboration with other specialist laboratories in the UK, Europe and outside Europe for further exploratory tests may occur. This would involve the transfer of serum, urine, plasma, saliva, SAM strips, nasopharyngeal samples, PBMC and/or other study samples to these laboratories, but these would remain anonymised. Informed consent for this will be gained from volunteers.

Immunological assays will be conducted according to local SOPs.

Subjects will be informed that there may be leftover samples of their blood (after all testing for this study is completed), and that such samples may be stored indefinitely for possible future research (exploratory immunology), including genotypic testing of genetic polymorphisms potentially relevant to vaccine immunogenicity. Subjects will be able to decide if they will permit such future use of any leftover samples. With the volunteers’ informed consent, any leftover cells, urine and serum/plasma will be frozen indefinitely for future analysis of COVID-19 and other coronaviruses related diseases or vaccine-related responses. If a subject elects not to permit this, all of that subject’s leftover samples will be discarded after the required period of storage to meet Good Clinical Practice (GCP) and regulatory requirements.

Samples that are to be stored for future research will be transferred to the OVC Biobank (REC 16/SC/0141).

## Study visits

The study visits and procedures will be undertaken by one of the clinical trials team. The procedures to be included in each visit are documented in the schedule of attendances (Table 8). Each visit is assigned a time-point and a window period, within which the visit will be conducted. In order to obtain core study data if a participant is unable to attend a study visit, the visit will be conducted by phone or video consultation and information recorded in the CRF.

### Screening visit

Participants will be required to complete an online questionnaire as an initial confirmation of eligibility. All potential volunteers will have a screening visit, which may take place up to 90 days prior to vaccination. At the screening visit, a video presentation of the aims of the study and all tests to be carried out may be screened to an audience. Individually each volunteer will have the opportunity to question an appropriately trained and delegated researcher before signing the consent. Informed consent will be taken before screening, as described in section 6.2. If consent is obtained, the procedures indicated in the schedule of attendances will be undertaken including a medical history, physical examination, blood tests and height and weight. To avoid unnecessary additional venepuncture, if the appropriate blood test results for screening are available for the same volunteer from a screening visit for another study, these results may be used for assessing eligibility as long as the blood samples were taken up to 90 days prior to vaccination.

To corroborate medical history, general practitioner records will be consulted with the written permission of the subject after screening when possible and practical to do so (see section 6.2). GPs will be notified that the subject has volunteered for the study. During the screening, the volunteers will be asked to provide their National Insurance or passport number so that this can be entered on to a national database which helps prevent volunteers from participating in more than one clinical trial simultaneously or over-volunteering for clinical trials ([www.tops.org.uk](http://www.tops.org.uk)).

Abnormal clinical findings from the urinalysis or blood tests at screening will be assessed by a medically qualified study member. Abnormal blood tests following screening will be assessed according to specific laboratory adverse event grading tables. Any abnormal test result deemed clinically significant may be repeated to ensure it is not a single occurrence. If an abnormal finding is deemed to be clinically significant, the volunteer will be informed and appropriate medical care arranged with the permission of the volunteer.

The eligibility of the volunteer will be reviewed at the end of the screening visit and again when all results from the screening visit have been considered. Decisions to exclude the volunteer from enrolling in the trial or to withdraw a volunteer from the trial will be at the discretion of the Investigator. If eligible, a day 0 visit will be scheduled for the volunteer to receive the vaccine and subsequent follow-up.

### Day 0: Enrolment and vaccination visit

Volunteers will be considered enrolled into the trial at the point of vaccination. Before vaccination/trial intervention, the eligibility of the volunteer will be reviewed. Pulse, blood pressure and temperature will be observed and if necessary, a medical history and physical examination may be undertaken to determine need to withdraw the participant. For groups 4 and 5, the vaccination appointment will take place at least 12 weeks after receipt of their second intramuscular vaccination. Vaccinations will be administered as described below.

#### Vaccination

All vaccines will be administered intranasally according to specific SOPs. Participants will remain at the trial site for observation, in case of immediate adverse events for 60 mins (+/- 30 mins) post vaccination. To monitor for adverse events, a set of observations will be taken during this time at approximately 30 mins and/or 60 mins post vaccination.

In all groups, volunteers will be given an oral thermometer and diary card (paper or electronic), with instructions on use, along with the emergency 24-hour telephone number to contact the on-call study physician if needed. Volunteers will be instructed on how to self-assess the severity of solicited AEs. There will also be space on the diary card to self-document unsolicited AEs, and whether medication was taken to relieve the symptoms. Diary cards will collect information on the timing and severity of the following solicited AEs:

| Local solicited AEs | Systemic solicited AEs |
| --- | --- |
| Nasal tenderness/ pain | Fever |
| Nasal irritation | Feverishness |
| Nasal discharge | Chills |
| Sore throat | Joint pains (arthralgia) |
| Cough | Muscle pains (myalgia) |
| Sneezing | Fatigue |
|  | Headache |
|  | Malaise |
|  | Nausea |
|  | Vomiting |

Table 6 local and systemic solicited AEs

In addition, the following solicited AEs (‘additional solicited AEs’) will be collected at clinic visits:

| Headache |
| --- |
| Spontaneous bruising |

Table 7 Additional solicited AEs for collection at clinic visits

#### Post-vaccine fever and self-isolation

As fever is a common side effect of vaccination, participants will be given the following advice re: post-vaccine fever self-isolation. If a participant develops fever in the first 24-48 hours post-vaccination they and their household members should self-isolate as per government guidance. If the fever does not persist, it is likely to be a vaccine effect. In this case, they and their household, will be able to stop self-isolating once they have been afebrile for 24 hours. If the fever continues, they and their household, will be advised to continue self-isolating and arrange COVID-19 testing as per the government guidance.

#### **Sequence of Enrolment and Vaccination of Volunteers**

- For safety reasons, the first volunteer to receive the IMP at the low dose (5x10^9^vp ChAdOx1 nCOV-19) will be vaccinated ahead of any other participants and the profile of adverse events will be reviewed approximately 72 (+/-24) hours post vaccination.
- Provided there are no safety concerns, as assessed by the chief investigator, another 5 volunteers will be vaccinated at low dose.
- Seven days after the first 6 participants have received the IMP relevant investigators and the chair of DSMB will be asked to provide a decision on whether further vaccinations can go ahead. A full DSMB may also be consulted should safety concerns arise at this point.
- Following this review, 3 volunteers will be vaccinated with the higher dose of the IMP (5x10^10^vp ChAdOx1 nCOV-19) and will be reviewed for adverse events by the chief investigator approximately 72 (+/-24) hours post vaccination.
- If there are no safety concerns, the remaining volunteers in group 2 (5x10^10^vp ChAdOx1 nCOV-19) and group 3 (2x10^10^ vp ChAdOx1 nCOV-19) will be vaccinated concurrently. Please see section 8.5 regarding rationale for choice of dose level for each group.
- Enrolment of groups 4 and 5 may begin immediately upon receipt of ethical and regulatory approval.

Figure 8: *Summary of enrolment sequence*

### Subsequent visits:

Follow-up visits will take place as per the schedule of attendances described in Table 8.

At visits on days 7 and 14 (all volunteers), as well as days 35 and 42 (those randomised to receive a booster only), volunteers will be asked to report additional solicited AEs, listed in Table 7.

Blood sampling, nasopharyngeal & SAM strip sampling and physical examination may take place at these time points, as detailed in the table of procedures. Visits that do not require physical examination or blood sampling may be undertaken by telephone/ video call, as indicated in Table 8**.**

If volunteers experience adverse events (laboratory or clinical), which the investigator (physician), CI and/or DSMB chair determine necessary for further close observation, the volunteer may be admitted to an NHS hospital for observation and further medical management under the care of the Consultant on call.

| Attendance Number^#^ | 1 | 2 | 3a^$^ | 3b^&^ | 4 | 5 | 6 | 7 | 8 | 9 | 10 |
| --- | --- | --- | --- | --- | --- | --- | --- | --- | --- | --- | --- |
| Timeline** (days) | S | 0 | 1 | 3 | 7 | 14 | 28 | 35* | 42* | 56 | 112 |
| Time window (days) | N/A | N/A | 0 | +/-1 | +/-3 | +/-7 | +/-7 | +/-3 | +/-3 | +/-7 | +/-14 |
| Visit conducted by Telephone/Video call |  |  |  | (x) |  | (x) | (x) |  |  | (x) | (x) |
| Informed Consent | x |  |  |  |  |  |  |  |  |  |  |
| Review inclusion and exclusion criteria | x | x |  |  |  |  | x |  |  |  |  |
| Randomisation |  | x |  |  |  |  |  |  |  |  |  |
| Intranasal vaccination |  | x |  |  |  |  | x |  |  |  |  |
| Vital signs^ | x | x |  |  |  |  | x |  |  |  |  |
| Ascertainment of adverse events |  | x | x | x | x | x | x | x | x | x | x |
| Diary cards provided |  | x |  |  |  |  | x* |  |  |  |  |
| Medical History | x | (x) |  | (x) | (x) | (x) | (x) | (x) | (x) | (x) | (x) |
| Physical Examination | x | (x) |  | (x) | (x) | (x) | (x) | (x) | (x) | (x) | (x) |
| Urinalysis | x |  |  |  |  |  |  |  |  |  |  |
| Urinary bHCG (women only) | x | x |  |  |  |  | x* |  |  |  |  |
| SAM strip |  | x |  |  | x | x | x |  | x | x | x |
| Nasopharyngeal swab | (x**^$^**) | (x**^$^**) | (x^$^) |  |  |  |  |  |  |  |  |
| Saliva |  | x |  |  | x | x | x |  | x | x | x |
| Biochemistry, Haematology (mL) | 5 |  |  |  | 5 | 5 | (5) | 5 | 5 | (5) | (5) |
| HBsAg, HCV Ab, HIV serology (mL) | 5 |  |  |  |  |  |  |  |  |  |  |
| Exploratory immunology (mL) |  | Up to 50 | (Up to 50) |  |  | (Up to 50) | (Up to 50) |  | (Up to 50) | (Up to 50) | (Up to 50) |
| HLA typing (mL) |  | 4 |  |  |  |  |  |  |  |  |  |
| Blood volume per visit (ml) | 10 | 54 | 50 | - | 5 | 55 | 55 | 5 | 55 | 55 | 55 |
| Cumulative blood volume (ml)^%^ | 10 | 64 | 114 |  | 119 | 174 | 229 | 234 | 289 | 344 | 399 |

Table 8. Schedule of visits

*Key: * volunteers randomised to receive second intranasal vaccination only; S = screening visit; (x) = optional/ if considered necessary; ^ = Vital signs includes pulse, blood pressure and temperature; ^&^= Groups 1a/2a only; ^$^= visit for nasopharyngeal sampling in selected volunteers in groups 2b/3/4/5 only (see section 7.3); **=Timeline is approximate only. Exact timings of visits relate to the day on enrolment, i.e. each visit must occur at indicated number of days after enrolment ± time window. ^%^=Cumulative blood volume for volunteers if blood taken as per schedule, and excluding any repeat safety blood test that may be necessary. Blood volumes may vary according to local site equipment and practices.* ^#^=*Additional visits may be scheduled for safety monitoring (see section 7.1). An additional 10 ml of blood may be taken at these visits.*

### Symptomatic volunteers

Measurement of vaccine efficacy is not an objective in this small study, but information about diagnoses of COVID-19 will be collected.

Volunteers will be asked to comply with the most up-to-date UK government guidance on testing and isolation for COVID-19. If they fulfil the criteria for self-isolation, they will be asked to contact the trial team, but not to attend the clinic for follow-up. In this case, a follow-up visit may be undertaken by telephone or video call. Any volunteers reporting COVID-19 symptoms (cough, fever, shortness of breath, loss of sense of smell or taste) will be advised to arrange COVID-19 testing via the NHS.

In order to collect information about any symptoms or diagnoses of COVID-19, volunteers will be asked to provide information about positive test results and symptoms of COVID-19 to the investigators. This will be done at follow-up visits or via an electronic diary. Severe illness due to COVID-19 is an AESI and would be investigated as such, as discussed in section 9.4.

### Randomisation / allocation

#### Randomisation to booster vaccination

Volunteers in groups 1-3 will be randomised on an open-label basis, at the enrolment visit, prior to vaccination, in a 1:1 ratio, to receive a booster vaccination or no booster vaccination. Group 1 (1a + 1b, n=6), Group 2 (2a + 2b, n=24), and Group 3 (n=24) will be randomised independently. Randomisation, and documentation of randomisation, will be electronic (using the RedCap randomisation module), with an envelope back-up system.

The volunteer in group 1a, an individual under the age of 30, had received the priming vaccine and been randomised to be boosted (but had not received the boost vaccine) prior to the MHRA’s email of 8 April requiring urgent safety measures including avoidance of further dosing of volunteers under the age of 30. In compliance with that, this individual will not receive the boost vaccine. Randomisation of volunteers in group 1a will continue without replacement with an additional volunteer to be boosted.

#### Allocation to groups

Groups 1 and 2a will be recruited first.

Following this, volunteers will be allocated to groups 2b and 3. 3 volunteers will be allocated to group 3. Thereafter, volunteers will be allocated alternately to groups 2b and 3. Due to the speed of the NHS vaccination roll-out, the number of potentially eligible volunteers has reduced significantly. Recruitment for group 2b and 3 may therefore be closed at the discretion of the investigator.

1. INVESTIGATIONAL PRODUCT AND TRIAL INTERVENTIONS

## Manufacturing and presentation

### Description of ChAdOx1 nCoV-19

ChAdOx1 nCoV-19 vaccine consists of the replication-deficient simian adenovirus vector ChAdOx1, containing the structural surface glycoprotein (Spike protein) antigens of SARS-CoV-2.

## Supply

An appropriate route of IMP supply to the trial, consistent with its urgency, volunteer safety & scientific robustness, has been discussed with MHRA through a Scientific Advice meeting. MHRA-authorised ‘commercial’ product (i.e. the product being supplied for intramuscular use under Regulation 174 or a successor authorisation) will be a gift to the University of Oxford from AstraZeneca and/or the Crown (Department of Health and Social Care and/or NHS). Additional trial and route-specific labelling will be affixed to the outer packaging, in line with an agreement with MHRA, supported by a route-specific IMPD, IB, and clinic SOP. For further detail please refer to the IMPD.

Alternatively, or in addition to the above, a more conventional route of supply may be used. This would entail release to trial, by an AstraZeneca / Medimmune or University of Oxford QP, of vials of non-commercial (clinical trial supply) vaccine, labelled for intranasal use in COV008. This product would be the same as that used in the Phase II/III studies of intramuscular ChAdOx1 nCoV-19 i.e. essentially equivalent to the ‘commercial’ product. For further detail please refer to the route-specific IMPD.

## Storage

The vaccine is stored at 2-8°C in a secure fridge, at the clinical site. All movements of the study vaccines will be documented in accordance with existing standard operating procedure (SOP). Vaccine accountability, storage, shipment and handling will be in accordance with relevant SOPs and forms.

## Administration

The vaccine is in a multi-dose vial which is stored at 2-8 degrees and does not require thawing. If the vaccine is stored outside 2-8°C it must be used within 6 hours. If stored at 2-8°C after the first vial puncture, it can be used within 48 hours.

The vaccine will be administered intranasally using a Mucosal Atomization Device (MAD; <https://medtree.co.uk/mad-mucosal-atomization-device-without-syringe>) according to the manufacturer’s instructions. This device is CE marked for use for nasal drug administration, i.e. the purpose for which it will be used here. All volunteers will be observed in the unit for a minimum of 1 hour after vaccination. During administration of the investigational products, Advanced Life Support drugs and resuscitation equipment will be immediately available for the management of anaphylaxis. Vaccination will be performed and the IMPs handled according to the relevant SOPs.

## Rationale for selected dose

The doses to be administered in this trial have been selected on the basis of clinical experience with intramuscularly administered ChAdOx1 nCOV-19 vaccine, the dose which achieved efficacy after intranasal administration in non-human primates (2.5 x 10^10^ vp), and experience from the IN PanAd3-RSV clinical trials. This is the first trial of this vaccine administered via the IN route, and thus a dose escalation approach will be taken to minimise any potential risks to participants. The first 6 volunteers will receive a lower dose of vaccine (5 x 10^9^ vp). Following satisfactory review of adverse events, the remaining volunteers will receive either:

1. The higher dose (5 x 10^10^ vp). This is the current licensed dose for IM administration of ChAdOx1 nCOV-19, and (given the concentration of the available IMP) will be administered in a volume of approximately 500 μL. This dose was also investigated and found to be well tolerated intranasally in PanAd3-RSV trial [15].
2. The intermediate dose (2x10^10^ vp). This will be administered in a volume of approximately 200 μL (i.e. 100 μL to each nostril). This matches the volume administered for the licensed intranasal vaccine Flumist/ FluenzTetra^TM^, and in vp terms is close to the lower dose found to be effective after intramuscular administration of ChAdOx1 nCOV-19.

The dose response relationship will be characterised in order to select the most appropriate dose for further studies. There is known to be ‘diminishing return’ when volumes of intranasally administered product are increased, due to an increasing amount of the product being swallowed rather than adhering to nasal mucosa. A lower dose may be preferable in view of scarcity of supply of COVID-19 vaccines.

The higher dose, rather than the intermediate dose, will be used for group 2a, to provide confidence in concurrent vaccination of groups 2b and 3. Dose escalation from 5x10^9^ to 5x10^10^ VP in a single step is routine in clinical trials of intramuscularly-administered adenovirus-vectored vaccines and was the strategy used in intranasal evaluation of PanAd3 RSV.

## Minimising environmental contamination with genetically modified organisms (GMO)

The study will be performed in accordance with the current version of the UK Genetically Modified Organisms (Contained Use) Regulations. Approved SOPs will be followed to minimise dissemination of the recombinant vectored vaccine virus into the environment. Each participant will remain in the clinic room for the first 15 minutes (of the 60-minute +/- 30min post vaccination observation period) after dosing to contain any vaccine expelled by sneezing. The devices used to deliver IN vaccine and any tissues used by the volunteers during the observation period will be disposed as GMO waste by autoclaving in accordance with the relevant SOPs.

## Compliance with Trial Treatment

All vaccinations will be administered by the research team and recorded in the CRF. The study medication will be at no time in the possession of the participant and compliance will not, therefore, be an issue.

## Accountability of the Trial Treatment

Accountability of the IMP and control vaccine will be conducted in accordance with the relevant SOPs.

## Concomitant Medication

As set out by the exclusion criteria, volunteers may not enter the study if they have received: any vaccine in the 28 days prior to enrolment or there is planned receipt of any other vaccine within 28 days of each vaccination, any investigational product within 28 days prior to enrolment or if receipt is planned during the study period, or if there is any use of immunosuppressant medication within 6 months prior to enrolment or if receipt is planned at any time during the study period ( except topical steroids and short course of low dose steroids < 14 day).

Participants who become eligible for a SARS-CoV-2 vaccine according to government guidance will be able to receive the vaccine, under advice outlined in section 6.5.

# ASSESSMENT OF SAFETY

Safety will be assessed by the frequency, incidence and nature of AEs and SAEs arising during the study.

## Definitions

### Adverse Event (AE)

An AE is any untoward medical occurrence in a volunteer, which may occur during or after administration of an IMP and does not necessarily have a causal relationship with the intervention. An AE can therefore be any unfavourable and unintended sign (including any clinically significant abnormal laboratory finding or change from baseline), symptom or disease temporally associated with the study intervention, whether or not considered related to the study intervention.

### Adverse Reaction (AR)

An AR is any untoward or unintended response to an IMP. This means that a causal relationship between the IMP and an AE is at least a reasonable possibility, i.e., the relationship cannot be ruled out. All cases judged by the reporting medical Investigator as having a reasonable suspected causal relationship to an IMP (i.e. possibly, probably or definitely related to an IMP) will qualify as AR.

Adverse events that may be related to the IMP are listed in the Investigator’s Brochure.

### Serious Adverse Event (SAE)

An SAE is an AE that results in any of the following outcomes, whether or not considered related to the study intervention.

| Death |
| --- |
| Life-threatening event (i.e., the volunteer was, in the view of the Investigator, at immediate risk of death from the event that occurred). This does not include an AE that, if it occurred in a more severe form, might have caused death. |
| Persistent or significant disability or incapacity (i.e., substantial disruption of one’s ability to carry out normal life functions). |
| Hospitalisation or prolongation of existing hospitalisation, regardless of length of stay, even if it is a precautionary measure for continued observation. Hospitalisation (including inpatient or outpatient hospitalisation for an elective procedure) for a pre-existing condition that has not worsened unexpectedly does not constitute a serious AE. |
| An important medical event (that may not cause death, be life threatening, or require hospitalisation) that may, based upon appropriate medical judgment, jeopardise the volunteer and/or require medical or surgical intervention to prevent one of the outcomes listed above. Examples of such medical events include allergic reaction requiring intensive treatment in an emergency room or clinic, blood dyscrasias, or convulsions that do not result in inpatient hospitalisation. |
| Congenital anomaly or birth defect. |

Table 9 *SAE definitions (See section 9.8 for further details of SAE criteria)*

### Serious Adverse Reaction (SAR)

An AE that is both serious and, in the opinion of the reporting Investigator or Sponsor delegate, believed to be possibly, probably or definitely due to an IMP or any other study treatments, based on the information provided.

Serious adverse events that the reporting investigator believes may be due to an interaction between the IMP and a concomitant medication, will be reported as possibly related to the IMP i.e., as a SAR.

### Suspected Unexpected Serious Adverse Reaction (SUSAR)

A SAR, the nature and severity of which is not consistent with the information about the medicinal product in question set out in the IB.

## Expectedness

No IMP related SAEs are expected in this study. All SARs will therefore be reported as SUSARs.

## Foreseeable Adverse Reactions:

The foreseeable ARs following vaccination with ChAdOx1 nCoV-19 are those listed AEs which will be monitored via diary cards in Table 6 section 7.4.2.1.

## Adverse Events of Special Interest (AESI)

| Severe COVID-19 disease will be defined using clinical criteria and will be an AESI, in particular to monitor the possibility of vaccine-enhanced disease. Detailed clinical parameters will be collected from medical records and aligned with agreed definitions as they emerge. These are likely to include, but are not limited to, oxygen saturation, need for oxygen therapy, respiratory rate, need for ventilatory support, imaging and blood test results, amongst other clinically relevant parameters. Acute respiratory distress, pneumonitis, acute cardiac injury, arrhythmia, septic-shock like syndrome and acute kidney injury related with COVID-19 disease will be monitored from medical records review of hospitalised participants. |
| --- |
| Neurological or neuro-psychiatric events of > Grade 2 severity |
| Eosinophilia as a marker skewed Th2 responses will be routinely monitored in participants attending their COVID-19 testing and follow-up visits. Marked eosinophilia of ≥ 1.5 x10^9^/L will be reported as SAEs. |
| AESI relevant to vaccination in general will also be monitored such as: generalised convulsion, Guillain-Barre Syndrome (GBS), Acute Disseminated Encephalomyelitis (ADEM), Anaphylaxis, Vasculitides in addition to serious solicited AEs will be monitored. |
| Venous thromboembolism and/or thrombocytopenia of >Grade 2 severity. |
| Anosmia |

Table 10. *Adverse events of special interest*

## Causality

For every AE, an assessment of the relationship of the event to the administration of the vaccine will be undertaken by the CI-delegated clinician. An interpretation of the causal relationship of the intervention to the AE in question will be made, based on the type of event; the relationship of the event to the time of vaccine administration; and the known biology of the vaccine therapy (Table 11). Alternative causes of the AE, such as the natural history of pre-existing medical conditions, concomitant therapy, other risk factors and the temporal relationship of the event to vaccination will be considered and investigated. Causality assessment will take place during planned safety reviews, interim analyses (e.g. if a holding or stopping rule is activated) and at the final safety analysis, except for SAEs, which should be assigned by the reporting investigator, immediately, as described in SOP OVC005 Safety Reporting for CTIMPs.

| 0 | **No Relationship** | No temporal relationship to study product ***and***  Alternate aetiology (clinical state, environmental or other interventions); ***and***  Does not follow known pattern of response to study product |
| --- | --- | --- |
| 1 | **Unlikely** | Unlikely temporal relationship to study product ***and***  Alternate aetiology likely (clinical state, environmental or other interventions) ***and***  Does not follow known typical or plausible pattern of response to study product. |
| 2 | **Possible** | Reasonable temporal relationship to study product; ***or***  Event not readily produced by clinical state, environmental or other interventions; ***or***  Similar pattern of response to that seen with other vaccines |
| 3 | **Probable** | Reasonable temporal relationship to study product; ***and***  Event not readily produced by clinical state, environment, or other interventions ***or***  Known pattern of response seen with other vaccines |
| 4 | **Definite** | Reasonable temporal relationship to study product; ***and***  Event not readily produced by clinical state, environment, or other interventions; ***and***  Known pattern of response seen with other vaccines |

Table 11. *Guidelines for assessing the relationship of vaccine administration to an AE.*

## Reporting Procedures for All Adverse Events

All local and systemic AEs occurring in the 28 days following each study vaccination observed by the Investigator or reported by the volunteer, whether or not attributed to study medication, will be recorded in electronic diaries or study database. All AEs that result in a volunteer’s withdrawal from the study will be followed up until a satisfactory resolution occurs (if the volunteer consents to this), or until a non-study related causality is assigned. SAEs and Adverse Events of Special Interest will be collected throughout the entire trial period.

## Assessment of severity

The severity of clinical and laboratory adverse events will be assessed according to scales based on FDA toxicity grading scales for healthy and adolescent volunteers enrolled in preventive vaccine clinical trials, listed in the study specific working instructions and Table 12/ Table 13 below.

| Vital Signs | **Grade 1**  **(mild)** | **Grade 2**  **(moderate)** | **Grade 3**  **(severe)** | **Grade 4**  **Potentially Life threatening** |
| --- | --- | --- | --- | --- |
| Fever (oral) | 38.0°C - 38.4°C | 38.5°C – 38.9°C | 39.0°C - 40°C | > 40°C |
| Tachycardia (bpm)* | 101 - 115 | 116 – 130 | >130 | A&E visit† or hospitalisation for arrhythmia |
| Bradycardia (bpm)** | 50 – 54 | 45 – 49 | <45 | A&E visit† or hospitalisation for arrhythmia |
| Systolic hypertension (mmHg) | 141 - 150 | 151 – 155 | ≥155 | A&E visit† or hospitalization for malignant hypertension |
| Diastolic hypertension (mmHg) | 91 - 95 | 96 – 100 | >100 | A&E visit† or hospitalization for malignant hypertension |
| Systolic hypotension (mmHg)*** | 85 - 89 | 80 – 84 | <80 | A&E visit† or hospitalization for hypotensive shock |
| Respiratory Rate –breaths per minute | 17 - 20 | 21-25 | >25 | Intubation |

Table 12. *Severity grading criteria for physical observations. *Taken after ≥10 minutes at rest **When resting heart rate is between 60 – 100 beats per minute. Use clinical judgement when characterising bradycardia among some healthy subject populations, for example, conditioned athletes. ***Only if symptomatic (e.g. dizzy/ light-headed) † see section 9.8.1 regarding A&E attendances*

| Grade | Description |
| --- | --- |
| Grade 0 | None |
| Grade 1 | Mild: Transient or mild discomfort (< 48 hours); No interference with activity; No medical intervention/therapy required |
| Grade 2 | Moderate: Mild to moderate limitation in activity – some assistance may be needed; no or minimal medical intervention/therapy required |
| Grade 3 | Severe: Marked limitation in activity, some assistance usually required; medical intervention/therapy required. |
| Grade 4 | Potentially Life-threatening: requires assessment in A&E† or hospitalisation |

Table 13. *Severity grading criteria for local and systemic AEs. † see section 9.8.1 regarding A&E attendances*

## Reporting Procedures for Serious AEs

In order to comply with current regulations on SAE reporting to regulatory authorities, the event will be documented accurately and notification deadlines respected. SAEs will be reported on the SAE forms to members of the study team immediately after the Investigators become aware of their occurrence, as described in SOP OVC005 Safety Reporting for CTIMPs. Copies of all reports will be forwarded for review to the Chief Investigator (as the Sponsor’s representative) within 24 hours of the Investigator being aware of the suspected SAE. The DSMB will be notified of SAEs that are deemed possibly, probably or definitely related to study interventions; the chair of DSMB will be notified immediately (within 24 hours) of the Sponsor being aware of their occurrence. SAEs will not normally be reported immediately to the ethical committee(s) unless there is a clinically important increase in occurrence rate, an unexpected outcome, or a new event that is likely to affect safety of trial volunteers, at the discretion of the Chief Investigator and/or DSMB. In addition to the expedited reporting above, the Investigator shall include all SAEs in the annual Development Safety Update Report (DSUR) report.

### Accident and Emergency (Emergency Department) Attendances

A&E attendances should not routinely be reported as SAEs unless they meet the SAE definition described in Table 9.

### Grade 4 Blood results

Grade 4 blood results will be reported as SAEs under the important medical event definition.

### Cases falling under Hy’s Law will be reported as SAEs.

A Hy’s Law Case is defined by FDA Guidance for Industry “Drug-Induced Liver Injury: Premarketing Clinical Evaluation” (2009). Any study subject with:

1. an increase in: Aspartate Aminotransferase (AST) **OR** Alanine Aminotransferase (ALT) ≥ 3x Upper Limit of Normal (ULN)
2. **AND** Total Bilirubin ≥2xULN
3. No other reason can be found to explain the combination of increases, e.g., elevated serum alkaline phosphatase (ALP) indicating cholestasis, viral hepatitis A, B or C, or another drug capable of causing the observed injury.

### Grade 3 thrombocytopenia will be reported as an SAE

Grade 3 thrombocytopenia, i.e. a platelet count of <100x10^9^/L, will be reported as an SAE.

## Reporting Procedures for SUSARS

All SUSARs will be reported by the sponsor delegate to the relevant Competent Authority and to the REC, AstraZeneca, and other parties as applicable. For fatal and life-threatening SUSARS, this will be done no later than 7 calendar days after the Sponsor or delegate is first aware of the reaction. Any additional relevant information will be reported within 8 calendar days of the initial report. All other SUSARs will be reported within 15 calendar days.

Principal Investigators will be informed of all SUSARs for the relevant IMP for all studies with the same Sponsor, whether or not the event occurred in the current trial.

## Development Safety Update Report

A Development Safety Update Report (DSUR) will be prepared annually, within 60 days of the anniversary of the first approval date from the regulatory authority for each IMP. A route-specific DSUR will be submitted by the CI (on behalf of the sponsor) to the Competent Authority, Ethics Committee, HRA (where required), Host NHS Trust and Sponsor.

## Procedures to be followed in the event of abnormal findings

Eligibility for enrolment in the trial in terms of laboratory findings will be assessed by clinically qualified staff. Abnormal clinical findings from medical history, examination or blood tests will be assessed as to their clinical significance throughout the trial. Laboratory AEs will be assessed using specific toxicity grading scales adapted from the FDA Toxicity Grading Scale for Healthy Adult and Adolescent Volunteers Enrolled in Preventive Vaccine Clinical Trials. If a test is deemed clinically significant, it may be repeated, to ensure it is not a single occurrence. If a test remains clinically significant, the volunteer will be informed and appropriate medical care arranged as appropriate and with the permission of the volunteer. Decisions to exclude the volunteer from enrolling in the trial or to withdraw a volunteer from the trial will be at the discretion of the Investigator.

## Interim Reviews

The safety profile will be assessed on an on-going basis by the Investigators. The CI and relevant Investigators (as per the trial delegation log) will also review safety issues and SAEs as they arise.

The process for reviews is detailed in section 7.4.2.3.

The DSMB will review safety data accumulated 7 days after vaccination of the final volunteer in group 1b.

Two to four days before the first participant receives their second vaccination, there will be a safety data review. The DSMB will make recommendations concerning the conduct, continuation or modification of the study.

## Data Safety Monitoring Board

A Data Safety Monitoring Board will be appointed to

1. periodically review and evaluate the accumulated study data for participant safety, study conduct, progress, and efficacy.
2. make recommendations concerning the continuation, modification, or termination of the trial.

There will be a minimum of three appropriately qualified committee members of whom one will be the designated chair. The DSMB will operate in accordance with the trial specific charter, which will be established before recruitment starts.

The chair of the DSMB may be contacted for advice and independent review by the Investigator or trial Sponsor in the following situations:

• Following any SAE deemed to be possibly, probably or definitively related to a study intervention.

• Any other situation where the Investigator or trial Sponsor feels independent advice or review is important.

The DSMB will review SAEs deemed possibly, probably or definitively related to study interventions. The DSMB will be notified within 24 hours of the Investigators’ being aware of their occurrence. The DSMB has the power to place the study on hold if deemed necessary following a study intervention-related SAE.

## Safety Group Holding Rules

Safety group holding rules have been developed considering the fact that this is the first study of a new route of administration.

Solicited AEs are those listed as foreseeable ARs in section 9.3 of the protocol, occurring within the first 7 days after vaccination (day of vaccination and six subsequent days). ‘Unsolicited adverse events’ are adverse events other than the foreseeable ARs occurring within the first 7 days, or any AEs occurring after the first 7 days after vaccination

### Group holding rules

Safety lead-in and dose escalation procedures are outlined in section 7.4.2.3. Group holding rules mentioned below will apply to study group 1 as a whole (1a + 1b), and group 2 as a whole (2a + 2b), because triggering of these rules requires combining data from a number of volunteers.

| Solicited local adverse events: | If two or more of the first 6, or more than 25% of doses of the vaccine at a given time point (e.g. Day 0, Day 28) in a study group are followed by the same Grade 3 solicited local adverse event beginning within 2 days after vaccination (day of vaccination and one subsequent day) and persisting at Grade 3 for >72 hrs |
| --- | --- |
| Solicited systemic adverse events: | If two or more of the first 6, or more than 25% of doses of the vaccine at a given time point (e.g. Day 0, Day 28) in a study group are followed by the same Grade 3 solicited systemic adverse event beginning within 2 days after vaccination (day of vaccination and one subsequent day) and persisting at Grade 3 for >72 hrs |
| Unsolicited adverse events: | If two or more of the first 6, or more than 25% of doses of the vaccine at a given time point (e.g. Day 0, Day 28) in a study group are followed by the same Grade 3 unsolicited adverse event beginning within 2 days after vaccination (day of vaccination and one subsequent day) and persisting at Grade 3 for >72 hrs |
| Laboratory adverse event: | If two or more of the first 6, or more than 25% of doses of the vaccine at a given time point (e.g. Day 0, Day 28) in a study group are followed by the same Grade 3 laboratory adverse event beginning within 3 days after vaccination and persisting at Grade 3 for >72 hrs |
| A serious adverse event considered possibly, probably or definitely related to vaccination occurs | If an SAE occurs in any one individual, which is possibly, probably or definitely related to vaccination this would trigger a holding rule. There are two exemptions from this rule, which would not activate a holding rule. These include:  SAEs reported under the Hy’s Law requirement will not necessarily trigger a holding rule. These cases will also be reviewed by the DSMB  COVID-19 related hospital admissions considered to be at least possibly related to ChAdOx1 nCoV-19 (e.g. if considered to be a clinical presentation of a disease enhancement episode). COVID-19 related SAEs will be regularly reviewed by the DSMB, and a single event will not trigger a holding rule. |

Table 14. *Group holding rules*

If any of the above holding rules are activated, then further vaccinations in any group will not occur until a safety review by the DSMB, study sponsor and the chief investigator has been conducted and it is deemed appropriate to restart dosing. The Regulatory Authority will be informed and a request to restart dosing with pertinent data will be submitted as a substantial amendment. The safety review will consider:

- The relationship of the AE or SAE to the vaccine.
- The relationship of the AE or SAE to the vaccine dose, or other possible causes of the event.
- If appropriate, additional screening or laboratory testing for other volunteers to identify those who may develop similar symptoms and alterations to the current Participant Information Sheet (PIS) are discussed.
- New, relevant safety information from ongoing research programs on the various components of the vaccine.

The local ethics committee and vaccine manufacturers will also be notified if a holding rule is activated or released.

All vaccinated volunteers will be followed for safety until resolution or stabilisation (if determined to be chronic sequelae) of their AEs.

### Individual stopping rules

In addition to the above stated group holding rules, stopping rules for individual volunteers will apply (i.e., indications to withdraw individuals from further vaccinations). Study participants who present with at least one of the following stopping rules will be withdrawn from further vaccination in the study:

| Local reactions: | The volunteer develops a Grade 3 local solicited AE considered possibly, probably or definitely related within 2 days after vaccination (day of vaccination and one subsequent day) and persisting continuously at Grade 3 for > 72hrs. |
| --- | --- |
| Laboratory AEs: | the volunteer develops a Grade 3 laboratory AE considered possibly, probably or definitely related within 7 days after vaccination and persisting continuously at Grade 3 for > 72hrs. |
| Systemic solicited adverse events: | the volunteer develops a Grade 3 systemic solicited AE considered possibly, probably or definitely related within 2 days after vaccination (day of vaccination and one subsequent day) and persisting continuously at Grade 3 for > 72hrs. |
| Unsolicited adverse events: | the volunteer has a Grade 3 adverse event, considered possibly, probably or definitely related to vaccination, persisting continuously at Grade 3 for >72hrs.   - the volunteer has a SAE considered possibly, probably or definitely related to vaccination. - the volunteer has an acute allergic reaction or anaphylactic shock following the administration of vaccine investigational product. - The volunteer develops a venous thromboembolism |

Table 15. *Individual stopping rules*

If a volunteer has an acute respiratory illness (moderate or severe illness with or without fever) or a fever (oral temperature greater than 37.8°C) at the scheduled time of administration of investigational product, the volunteer will not be enrolled and will be withdrawn from the study.

All vaccinated volunteers will be followed for safety until the end of their planned participation in the study or until resolution or stabilisation (if determined to be chronic sequelae) of their AEs, providing they consent to this.

Participants who met individual holding rules or were advised not to receive a booster dose as a result of an AE and have been offered a COVID-19 approved/licensed vaccine will be given an opportunity to discuss potential safety implications if they were to accept the offered vaccine with clinically qualified investigators.

In addition to these pre-defined criteria, the study can be put on hold upon advice of the DSMB, Chief Investigator, Study Sponsor, regulatory authority, Ethical Committee(s), for any single event or combination of multiple events which, in their professional opinion, jeopardise the safety of the volunteers or the reliability of the data.

# STATISTICS

## Description of Statistical Methods

Statistical analysis will be appropriate to the study’s primary aim, i.e. to provide a descriptive and preliminary assessment of the safety of intranasally-administered ChAdOx1 nCoV-19, with limited, highly preliminary immunological information being sought as a subsidiary aim.

Appropriate descriptive statistics and graphical representations will be used to present the safety and immunogenicity data, similar to those used in similar previous studies [31]. No statistical inference testing will be performed.

## Safety & Reactogenicity

Counts and percentages of each local and systemic solicited adverse reaction from diary cards, and all unsolicited AEs and SAEs will be presented for each group.

## Immunogenicity

Mucosal antibody responses to SARS-CoV-2 spike protein, as measured by ELISA or a similar quantitative isotype-specific antigen-binding assay, are a secondary outcome measure. Other immunological analyses will be purely exploratory.

Highly skewed antibody data will be log-transformed prior to analysis. The geometric mean concentration and associated 95% confidence interval will be summarised for each group at each timepoint, by computing the anti-log of the mean difference of the log-transformed data.

Spike-specific T cell responses (ELISpot) will be presented as means and confidence intervals, or medians and interquartile ranges if non-normally distributed at all post vaccination time points.

## Procedure for Accounting for Missing, Unused, and Spurious Data.

All available data will be included in the analysis.

## Inclusion in Analysis

All vaccinated participants will be included in the analysis.

# DATA MANAGEMENT

## Data Handling

The Chief Investigator will be responsible for all data that accrues from the study.

All study data including participant diary will be recorded directly into an Electronic Data Capture (EDC) system (REDCap) or onto a paper source document for later entry into EDC if direct entry is not available or is not practical at site. This includes safety data, laboratory data and outcome data. Any additional information that needs recording but is not relevant for the CRF (such as signed consent forms etc.) will be recorded on a separate paper source document. All documents will be stored safely and securely in confidential conditions.

All adverse event data (both solicited and unsolicited) reported by the volunteer will be entered onto a volunteer’s electronic diary card (eDiary) for a maximum of 28 days following administration of the IMP. The eDiary provides a full audit trail of edits and will be reviewed at each review time-point indicated in the schedule of events. Any adverse event continuing beyond the period of the diary will be copied into the eCRF and followed to resolution, if there is a causal relationship to the IMP, or to the end of the study if there is no causal relationship.

The participants will be identified by a unique trial specific number and code in any database. The name and any other identifying detail will NOT be included in any trial data electronic file.

The EDC system (CRF data) uses a relational database (MySQL/ PostgreSQL) via a secure web interface with data checks applied during data entry to ensure data quality. The database includes a complete suite of features which are compliant with GCP, EU and UK regulations and Sponsor security policies, including a full audit trail, user-based privileges, and integration with the institutional LDAP server. The MySQL and PostgreSQL database and the webserver will both be housed on secure servers maintained by the University of Oxford IT personal. The servers are in a physically secure location in Europe. Backups will be stored in accordance with the IT department schedule daily, weekly, monthly, and are retained for one month, three months, and six months, respectively. The IT servers provide a stable, secure, well-maintained, and high capacity data storage environment. REDCap is widely-used, powerful, reliable, well-supported systems. Access to the study's database will be restricted to the members of the study team by username and password.

## Record Keeping

The Investigators will maintain appropriate medical and research records for this trial, in compliance with GCP and regulatory and institutional requirements for the protection of confidentiality of volunteers. The Chief Investigator, co-Investigators and clinical research nurses will have access to records. The Investigators will permit authorised representatives of the Sponsor(s), as well as ethical and regulatory agencies to examine (and when required by applicable law, to copy) clinical records for the purposes of quality assurance reviews, audits and evaluation of the study safety and progress.

All trial records will be stored for a minimum of 5 years after the end of the trial at a secure archiving facility. If volunteers consent to be contacted for future research, information about their consent form will be recorded, retained and stored securely and separately from the research data. If volunteers consent to have their samples stored and used in future research, information about their consent form will be recorded, retained and stored securely as per Biobanking procedures and SOP.

## Source Data and Case Report Forms (CRFs)

All protocol-required information will be collected in CRFs designed by the Chief Investigator. All source documents will be filed in the CRF. Source documents are original documents, data, and records from which the volunteer’s CRF data are obtained. For this study, these will include, but are not limited to, volunteer consent form, blood results, GP response letters, laboratory records, diaries, medical records and correspondence. In the majority of cases, CRF entries will be considered source data as the CRF is the site of the original recording (i.e. there is no other written or electronic record of data). In this study this will include, but is not limited to medical history, medication records, vital signs, physical examination records, urine assessments, blood results, adverse event data and details of vaccinations. All source data and volunteer CRFs will be stored securely.

Source data verification requirements will be defined in the trial risk assessment and monitoring plan.

## Data Protection

The study protocol, documentation, data and all other information generated will be held in strict confidence. No information concerning the study or the data will be released to any unauthorised third party, without prior written approval of the sponsor.

## Data Quality

Data collection tools will undergo appropriate validation to ensure that data are collected accurately and completely. Datasets provided for analysis will be subject to quality control processes to ensure analysed data is a true reflection of the source data.

Trial data will be managed in compliance with local data management SOPs. If additional, study specific processes are required, an approved Data Management Plan will be implemented.

## Archiving

Study data may be stored electronically on a secure server, and paper notes will be kept in a key-locked filing cabinet at the site. All essential documents will be retained for a minimum of 5 years after the study has finished. The need to store study data for longer in relation to licensing of the vaccine will be subject to ongoing review. For effective vaccines that may be licensed, we may store research data securely at the site at least 15 years after the end of the study, subject to adjustments in clinical trials regulations. Participants’ bank details will be stored for 7 years in line with the site financial policy.

General archiving procedures will be conducted in compliance to SOP OVC020 Archiving.

# QUALITY CONTROL AND QUALITY ASSURANCE PROCEDURES

## Investigator procedures

Approved site-specific standard operating procedures (SOPs) will be used at all clinical and laboratory sites.

## Monitoring

Regular monitoring will be performed according to GCP by the Monitor. Following written SOPs and an approved, risk based monitoring plan, the monitor will verify that the clinical trial is conducted and data are generated, documented and reported in compliance with the protocol, GCP and the applicable regulatory requirements. The site will provide direct access to all trial related source data/documents and reports for the purpose of monitoring by the Monitor, auditing by the Sponsor and inspection by local and regulatory authorities.

## Protocol deviation

Any deviations from the protocol will be documented in a protocol deviation form and filed in the trial master file. Each deviation will be assessed as to its impact on volunteer safety and study conduct. Significant protocol deviations will be listed in the end of study report.

## Audit & inspection

The QA team conducts systems based internal audits to check that trials are being conducted according to local procedures and in compliance with study protocols, departmental SOPs, GCP and applicable regulations.

The Sponsor, trial sites, and ethical committee(s) may carry out audit to ensure compliance with the protocol, GCP and appropriate regulations.

GCP inspections may also be undertaken by the MHRA to ensure compliance with protocol and the Medicines for Human Use (Clinical Trials) Regulations 2004, as amended. The Sponsor will assist in any inspections and will support the response to the MHRA as part of the inspection procedure.

# SERIOUS BREACHES

The Medicines for Human Use (Clinical Trials) Regulations contain a requirement for the notification of "serious breaches" to the MHRA within 7 days of the Sponsor becoming aware of the breach.

A serious breach is defined as “A breach of GCP or the trial protocol which is likely to effect to a significant degree:

(a) the safety or physical or mental integrity of the subjects of the trial; or

(b) the scientific value of the trial”.

In the event that a potential serious breach is suspected the Sponsor will be informed as soon as possible, to allow preliminary assessment of the breach and reporting to the MHRA within the required timelines.

# ETHICS AND REGULATORY CONSIDERATIONS

## Declaration of Helsinki

The Investigators will ensure that this study is conducted according to the principles of the current revision of the Declaration of Helsinki.

## Guidelines for Good Clinical Practice

The Investigator will ensure that this trial is conducted in accordance with relevant regulations and with Good Clinical Practice.

## Ethical and Regulatory Approvals

Following Sponsor approval the protocol, informed consent form, participant information sheet and any proposed advertising material will be submitted to an appropriate Research Ethics Committee (REC), HRA (where required), regulatory authorities (MHRA in the UK), and host institution(s) for written approval. No amendments to this protocol will be made without consultation with, and agreement of, the Sponsor.

The Investigator is responsible for ensuring that changes to an approved trial, during the period for which regulatory and ethical committee(s) approval has already been given, are not initiated without regulatory and ethical committee(s)’ review and approval except to eliminate apparent immediate hazards to the subject (i.e as an Urgent Safety Measure).

## Volunteer Confidentiality

The study will comply with the UK GDPR and Data Protection Act 2018, which require data to be de-identified as soon as it is practical to do so. The processing of personal data of participants will be minimised by making use of a unique participant study number only on all study documents and any electronic database(s), with the exception of informed consent forms and participant ID logs. All documents will be stored securely and only accessible by study staff and authorised personnel. The study staff will safeguard the privacy of participants’ personal data. A separate confidential file containing identifiable information will be stored in a secured location in accordance with the current data protection legislation. This material may be shown to other professional staff, used for educational purposes, or included in a scientific publication.

# FINANCING AND INSURANCE

## Financing

The study is funded by AstraZeneca UK Ltd and the University of Oxford.

## Insurance

The University has a specialist insurance policy in place which would operate in the event of any participant suffering harm as a result of their involvement in the research (Newline Underwriting Management Ltd, at Lloyd’s of London). NHS indemnity operates in respect of the clinical treatment which is provided.

## Compensation

Volunteers will be compensated for their time, the inconvenience of having blood tests and procedures, and their travel expenses. The total amount compensated will be up to £<insert> depending on the exact number of visits, and whether any repeat or additional visits are necessary. They will be compensated £25 for attending the screening visit. For all other trial visits as outlined in Table 8, compensation will be calculated according to the following:

- Travel expenses: £15 per visit
- Inconvenience of blood tests: £10 per blood donation
- Time required for visit: £20 per hour

Should the volunteer decide to withdraw from the trial before it is completed, payment will be pro rata.

# Publication Policy

The Investigators will be involved in reviewing drafts of the manuscripts, abstracts, press releases and any other publications arising from the study. Data from the study may also be used as part of a thesis for a PhD or MD.

# Development of a new product/ process or the generation of intellectual property

Ownership of IP generated by employees of the University vests in the University. The protection and exploitation of any new IP is managed by the University’s technology transfer office, Oxford University Innovations. Investigators in this study may benefit from the royalty sharing policy of the University if new intellectual property is generated from the trial. Several investigators are applicants or co-inventors on previous patent filings or patents related to ChAdOx1 vaccines. The University of Oxford, which is partnered with the Oxford University Hospitals NHS Foundation Trust in the NIHR Oxford Biomedical Research Centre, is committed to the translational progress and commercial development of healthcare products potentially meeting medical and global health needs, and does and will work with commercial partners towards these goals.

References

1. Zhu, N., et al., *A Novel Coronavirus from Patients with Pneumonia in China, 2019.* New England Journal of Medicine, 2020. **382**(8): p. 727-733.

2. Folegatti, P.M., et al., *Safety and immunogenicity of the ChAdOx1 nCoV-19 vaccine against SARS-CoV-2: a preliminary report of a phase 1/2, single-blind, randomised controlled trial.* Lancet, 2020. **396**(10249): p. 467-478.

3. Romero-Steiner, S., et al., *Reduction in functional antibody activity against Streptococcus pneumoniae in vaccinated elderly individuals highly correlates with decreased IgG antibody avidity.* Clinical Infectious Diseases, 1999. **29**(2): p. 281-288.

4. Li, F., *Structure, Function, and Evolution of Coronavirus Spike Proteins.* Annual review of virology, 2016. **3**(1): p. 237-261.

5. Zhou, P., et al., *A pneumonia outbreak associated with a new coronavirus of probable bat origin.* Nature, 2020.

6. Alharbi, N.K., et al., *ChAdOx1 and MVA based vaccine candidates against MERS-CoV elicit neutralising antibodies and cellular immune responses in mice.* Vaccine, 2017. **35**(30): p. 3780-3788.

7. van Doremalen, N., et al., *ChAdOx1 nCoV-19 vaccine prevents SARS-CoV-2 pneumonia in rhesus macaques.* Nature, 2020. **586**(7830): p. 578-582.

8. van Doremalen, N., et al., *ChAdOx1 nCoV-19 vaccination prevents SARS-CoV-2 pneumonia in rhesus macaques.* bioRxiv: The Preprint Server for Biology, 2020.

9. van Doremalen, N., et al., *Intranasal ChAdOx1 nCoV-19/AZD1222 vaccination reduces shedding of SARS-CoV-2 D614G in rhesus macaques.* bioRxiv: The Preprint Server for Biology, 2021.

10. Folegatti, P.M., et al., *Safety and immunogenicity of the ChAdOx1 nCoV-19 vaccine against SARS-CoV-2: a preliminary report of a phase 1/2, single-blind, randomised controlled trial.* Lancet (London, England), 2020. **396**(10249): p. 467-478.

11. Ramasamy, M.N., et al., *Safety and immunogenicity of ChAdOx1 nCoV-19 vaccine administered in a prime-boost regimen in young and old adults (COV002): a single-blind, randomised, controlled, phase 2/3 trial.* The Lancet, 2020. **396**(10267): p. 1979-1993.

12. Voysey, M., et al., *Safety and efficacy of the ChAdOx1 nCoV-19 vaccine (AZD1222) against SARS-CoV-2: an interim analysis of four randomised controlled trials in Brazil, South Africa, and the UK.* Lancet (London, England), 2021. **397**(10269): p. 99-111.

13. Voysey, M., et al., *Single Dose Administration, And The Influence Of The Timing Of The Booster Dose On Immunogenicity and Efficacy Of ChAdOx1 nCoV-19 (AZD1222) Vaccine*. 2021, Social Science Research Network: Rochester, NY.

14. Green, C.A., et al., *Novel genetically-modified chimpanzee adenovirus and MVA-vectored respiratory syncytial virus vaccine safely boosts humoral and cellular immunity in healthy older adults.* The Journal of Infection, 2019. **78**(5): p. 382-392.

15. Green, C.A., et al., *Chimpanzee adenovirus- and MVA-vectored respiratory syncytial virus vaccine is safe and immunogenic in adults.* Science Translational Medicine, 2015. **7**(300): p. 300ra126.

16. Robert-Guroff, M., *Replicating and non-replicating viral vectors for vaccine development.* Curr.Opin.Biotechnol., 2007. **18**(6): p. 546-556.

17. Tasker, S., et al., *2554. Safety and Immunogenicity of NasoVAX, a Novel Intranasal Influenza Vaccine.* Open Forum Infectious Diseases, 2018. **5**(Suppl 1): p. S68.

18. Van Kampen, K.R., et al., *Safety and immunogenicity of adenovirus-vectored nasal and epicutaneous influenza vaccines in humans.* Vaccine, 2005. **23**(8): p. 1029-1036.

19. Evans, R.K., et al., *Development of stable liquid formulations for adenovirus-based vaccines.* J Pharm Sci, 2004. **93**(10): p. 2458-75.

20. Tseng, C.T., et al., *Immunization with SARS coronavirus vaccines leads to pulmonary immunopathology on challenge with the SARS virus.* PLoS One, 2012. **7**(4): p. e35421.

21. Weingartl, H., et al., *Immunization with modified vaccinia virus Ankara-based recombinant vaccine against severe acute respiratory syndrome is associated with enhanced hepatitis in ferrets.* J Virol, 2004. **78**(22): p. 12672-6.

22. Liu, L., et al., *Anti-spike IgG causes severe acute lung injury by skewing macrophage responses during acute SARS-CoV infection.* JCI insight, 2019. **4**(4): p. e123158.

23. Agrawal, A.S., et al., *Immunization with inactivated Middle East Respiratory Syndrome coronavirus vaccine leads to lung immunopathology on challenge with live virus.* Hum Vaccin Immunother, 2016. **12**(9): p. 2351-6.

24. Munster, V.J., et al., *Protective efficacy of a novel simian adenovirus vaccine against lethal MERS-CoV challenge in a transgenic human DPP4 mouse model.* NPJ Vaccines, 2017. **2**: p. 28.

25. Alharbi, N.K., et al., *Humoral Immunogenicity and Efficacy of a Single Dose of ChAdOx1 MERS Vaccine Candidate in Dromedary Camels.* Sci Rep, 2019. **9**(1): p. 16292.

26. Gao, Q., et al., *Development of an inactivated vaccine candidate for SARS-CoV-2.* Science, 2020: p. eabc1932.

27. Oran, D.P. and E.J. Topol, *Prevalence of Asymptomatic SARS-CoV-2 Infection.* Annals of Internal Medicine, 2020. **173**(5): p. 362-367.

28. Johansson, M.A., et al., *SARS-CoV-2 Transmission From People Without COVID-19 Symptoms.* JAMA Network Open, 2021. **4**(1): p. e2035057.

29. Beyer, W.E.P., et al., *Cold-adapted live influenza vaccine versus inactivated vaccine: systemic vaccine reactions, local and systemic antibody response, and vaccine efficacy: A meta-analysis.* Vaccine, 2002. **20**(9): p. 1340-1353.

30. Lai, P.S., et al., *Alternate methods of nasal epithelial cell sampling for airway genomic studies.* Journal of Allergy and Clinical Immunology, 2015. **136**(4): p. 1120-1123.e4.

31. Folegatti, P.M., et al., *Safety and Immunogenicity of a Novel Recombinant Simian Adenovirus ChAdOx2 as a Vectored Vaccine.* Vaccines, 2019. **7**(2).

# APPENDIX A: AMENDMENT HISTORY

| **Amendment No.** | **Protocol Version No.** | **Date issued** | **Author(s) of changes** | **Details of Changes made** |
| --- | --- | --- | --- | --- |
| N/A | 1.0 | 9^th^ March 2021 | Meera Madhavan, Alexander Douglas, Adam Ritchie, Daniel Jenkin, Iona Tarbet | Issued |
| SA001 | 2.0 | 20^th^ April 2021 | Alexander Douglas, Adam Ritchie, Meera Madhavan, Iona Tarbet | - Clarified procedures and guidance around receiving non-study COVID-19 vaccines: interval between IN and any IM COVID vaccination standardised to 28 days in line with other documents. - Confidentiality (14.4) amended to remove erroneous reference to photographs of injection site. - Added day 42 visit - Exclusion criteria (age <30, history of venous thromboembolism or receipt of heparin) and additional risk mitigations re thrombosis added (day 14 Plt, spontaneous bruising as solicited AE, grade 3 thrombocytopenia as SAE, grade 2 thrombocytopenia and venous thromboembolism as AESIs) - Pre-vaccination and day 1 NP swab/brush for group 2b added, at investigators’ discretion - Funding updated to include AstraZeneca - Addition of group 3 (2x10^10^vp IN). - Reduction of boost dose in group 1 to 5x10^9^ VP - Typographical errors corrected in appendix B - Section 6.3.5 addition of information on retention of volunteers already enrolled who are no longer eligible due to age eligibility changes - Section 7.4.5 updated to clarify randomisation and allocation procedures. |
| SA003 | 3.0 | ---- | Alexander Douglas, Adam Ritchie, Iona Tarbet | - Change of eligibility criteria to include adults aged 18-55 years old. - Clarification of investigators’ neutral stance on ongoing participants wishing to defer NHS vaccination by up to 56 days after enrolment. - Neuro-psychiatric events of > Grade 2 severity and anosmia added as AESIs. |
| SA005 | 4.0 | ------- | Meera Madhavan, Iona Tarbet, Alexander Douglas | - Study duration increased from 10 months to 12 months - Groups 2b and 3 size reduced to ‘up to 15’ and ‘up to 18’ respectively. - Added: recruitment of groups 2b/3 may be closed at investigator discretion - Addition of groups 4 and 5, with appropriate inclusion and exclusion criteria and revision of expected study duration. - Addition of history of central nervous system disorders as exclusion criterion. |

# Appendix B. Toxicity grading scale for Lab AEs

| Haematology |  |  | Lab Range | Grade 1 | Grade 2 | Grade 3 | Grade 4 |
| --- | --- | --- | --- | --- | --- | --- | --- |
| Haemoglobin Absolute | **Male** | g/l | 130 - 170 | 115-125 | 100-114 | 85-99 | <85 |
| Haemoglobin Absolute | **Female** |  | 120 - 150 | 105-113 | 90-104 | 80-89 | <80 |
| Haemoglobin Change from Baseline (Decrease) |  |  | n/a | 10-15 | 16-20 | 21-50 | >50 |
| White Blood Cells | **Elevated** | x10^9^/l | 11 | 11.5-15.00 | 15.01-20 | 20.01-25 | >25 |
| White Blood Cells | **Low** | x10^9^/l | 4.0 | 2.5-3.5 | 1.5-2.49 | 1.0-1.49 | <1.0 |
| Platelets | **Low** | x10^9^/l | 150-400 | 125-140 | 100-124 | 25-99 | <25 |
| Neutrophils | **Low** | x10^9^/l | 2.0-7.0 | 1.5-1.99 | 1.0-1.49 | 0.5-0.99 | <0.50 |
| Lymphocytes | **Low** | x10^9^/l | 1.0-4.0 | 0.75-0.99 | 0.5-0.74 | 0.25-0.49 | <0.25 |
| Eosinophils | **Elevated** | x10^9^/l | 0.02 - 0.5 | 0.65-1.5 | 1.51-5.00 | >5.00 | Hypereosinophilia |
|  |  |  |  |  |  |  |  |
| Biochemistry |  |  |  |  |  |  |  |
| Sodium | **Elevated** | mmol/l | 145 | 146-147 | 148-149 | 150-155 | >155 |
| Sodium | **Low** |  | 135 | 132-134 | 130-131 | 125-129 | <125 |
| Potassium | **Elevated** | mmol/l | 5 | 5.1-5.2 | 5.3-5.4 | 5.5-6.5 | >6.5 |
| Potassium | **Low** |  | 3.5 | 3.2-3.3 | 3.1 | 2.5-3.0 | <2.5 |
| Urea | **Elevated** | mmol/l | 2.5 - 7.4 | 8.2-9.3 | 9.4-11.0 | >11.0 | Requires dialysis |
| Creatinine | **Elevated** | µmol/l | 49 - 104 | 1.1-1.5xULN  114-156 | >1.5-3.0xULN  157-312 | >3.0xULN  >312 | Requires dialysis |
| Bilirubin | **Normal LFTs** | µmol/l | 0-21 | 1.1-1.5xULN  23-32 | >1.5-2xULN  33-42 | >2-3xULN  43-63 | >3xULN  ≥64 |
| Bilirubin | **Abnormal LFTs** | µmol/l | 0 - 21 | 1.1-1.25xULN  23-26 | >1.25-1.5xULN  27-32 | >1.5-1.75xULN  33-37 | >1.75xULN  >37 |
| ALT |  | IU/l | 10 - 45 | 1.1-2.5xULN  49-112 | >2.5-5xULN  113-225 | >5-10xULN  226-450 | >10xUPN  >450 |
| Alk Phosphatase | **Elevated** | IU/l | 30 -130 | 1.1-2xULN  143-260 | >2-3xULN  261-390 | >3-10xULN  391-1300 | >10xULN  >1300 |
| Albumin |  | g/l | 32-50 | 28-31 | 25-27 | <25 | - |

Normal lab ranges may vary between sites and should be adapted accordingly
